# Genomic Analysis of the Necrotrophic Fungal Pathogens *Sclerotinia sclerotiorum* and *Botrytis cinerea*

**DOI:** 10.1371/journal.pgen.1002230

**Published:** 2011-08-18

**Authors:** Joelle Amselem, Christina A. Cuomo, Jan A. L. van Kan, Muriel Viaud, Ernesto P. Benito, Arnaud Couloux, Pedro M. Coutinho, Ronald P. de Vries, Paul S. Dyer, Sabine Fillinger, Elisabeth Fournier, Lilian Gout, Matthias Hahn, Linda Kohn, Nicolas Lapalu, Kim M. Plummer, Jean-Marc Pradier, Emmanuel Quévillon, Amir Sharon, Adeline Simon, Arjen ten Have, Bettina Tudzynski, Paul Tudzynski, Patrick Wincker, Marion Andrew, Véronique Anthouard, Ross E. Beever, Rolland Beffa, Isabelle Benoit, Ourdia Bouzid, Baptiste Brault, Zehua Chen, Mathias Choquer, Jérome Collémare, Pascale Cotton, Etienne G. Danchin, Corinne Da Silva, Angélique Gautier, Corinne Giraud, Tatiana Giraud, Celedonio Gonzalez, Sandrine Grossetete, Ulrich Güldener, Bernard Henrissat, Barbara J. Howlett, Chinnappa Kodira, Matthias Kretschmer, Anne Lappartient, Michaela Leroch, Caroline Levis, Evan Mauceli, Cécile Neuvéglise, Birgitt Oeser, Matthew Pearson, Julie Poulain, Nathalie Poussereau, Hadi Quesneville, Christine Rascle, Julia Schumacher, Béatrice Ségurens, Adrienne Sexton, Evelyn Silva, Catherine Sirven, Darren M. Soanes, Nicholas J. Talbot, Matt Templeton, Chandri Yandava, Oded Yarden, Qiandong Zeng, Jeffrey A. Rollins, Marc-Henri Lebrun, Marty Dickman

**Affiliations:** 1 Unité de Recherche Génomique – Info, UR1164, INRA, Versailles, France; 2 Biologie et Gestion des Risques en Agriculture – Champignons Pathogènes des Plantes, UR1290, INRA, Grignon, France; 3 Broad Institute of MIT and Harvard, Cambridge, Massachusetts, United States of America; 4 Laboratory of Phytopathology, Wageningen University, Wageningen, The Netherlands; 5 Departamento de Microbiología y Genética, Centro Hispano-Luso de Investigaciones Agrarias, Universidad de Salamanca, Salamanca, Spain; 6 GENOSCOPE, Centre National de Séquençage, Evry, France; 7 Architecture et Fonction des Macromolécules Biologiques, UMR6098, CNRS – Université de la Méditerranée et Université de Provence, Marseille, France; 8 Microbiology and Kluyver Centre for Genomics of Industrial Fermentations, Utrecht, The Netherlands; 9 CBS-KNAW Fungal Biodiversity Centre, Utrecht, The Netherlands; 10 School of Biology, University of Nottingham, Nottingham, United Kingdom; 11 Biologie et Génétique des Interactions Plante-Parasite, CIRAD – INRA – SupAgro, Montpellier, France; 12 Faculty of Biology, Kaiserslautern University, Kaiserslautern, Germany; 13 Biology Department, University of Toronto, Mississauga, Canada; 14 Botany Department, La Trobe University, Melbourne, Australia; 15 Laboratoire de Génomique Fonctionnelle des Champignons Pathogènes de Plantes, UMR5240, Université de Lyon 1 – CNRS – BAYER S.A.S., Lyon, France; 16 Department of Molecular Biology and Ecology of Plants, Tel Aviv University, Tel Aviv, Israel; 17 Instituto de Investigaciones Biologicas – CONICET, Universidad Nacional de Mar del Plata, Mar del Plata, Argentina; 18 Molekularbiologie und Biotechnologie der Pilze, Institut für Biologie und Biotechnologie der Pflanzen, Münster, Germany; 19 Landcare Research, Auckland, New Zealand; 20 Interactions Biotiques et Santé Plantes, UMR5240, INRA – Université de Nice Sophia-Antipolis – CNRS, Sophia-Antipolis, France; 21 Laboratoire d'Ecologie, Systématique et Evolution, Université Paris-Sud – CNRS – AgroParisTech, Orsay, France; 22 Departamento de Bioquímica y Biología Molecular, Universidad de La Laguna, Tenerife, Spain; 23 Helmholtz Zentrum München, German Research Center for Environmental Health, Institute of Bioinformatics and Systems Biology, Neuherberg, Germany; 24 School of Botany, University of Melbourne, Melbourne, Australia; 25 Biologie Intégrative du Métabolisme Lipidique Microbien, UMR1319, INRA – Micalis – AgroParisTech, Thiverval-Grignon, France; 26 Fundacion Ciencia para la Vida and Facultad de Ciencias Biologicas, Universidad Andres Bello, Santiago, Chile; 27 School of Biosciences, University of Exeter, Exeter, United Kingdom; 28 Plant and Food Research, Mt. Albert Research Centre, Auckland, New Zealand; 29 Department of Plant Pathology and Microbiology, Hebrew University Jerusalem, Rehovot, Israel; 30 Department of Plant Pathology, University of Florida, Gainesville, Florida, United States of America; 31 Institute for Plant Genomics and Biotechnology, Borlaug Genomics and Bioinformatics Center, Department of Plant Pathology and Microbiology, Texas A&M University, College Station, Texas, United States of America; Progentech, United States of America

## Abstract

*Sclerotinia sclerotiorum* and *Botrytis cinerea* are closely related necrotrophic plant pathogenic fungi notable for their wide host ranges and environmental persistence. These attributes have made these species models for understanding the complexity of necrotrophic, broad host-range pathogenicity. Despite their similarities, the two species differ in mating behaviour and the ability to produce asexual spores. We have sequenced the genomes of one strain of *S. sclerotiorum* and two strains of *B. cinerea*. The comparative analysis of these genomes relative to one another and to other sequenced fungal genomes is provided here. Their 38–39 Mb genomes include 11,860–14,270 predicted genes, which share 83% amino acid identity on average between the two species. We have mapped the *S. sclerotiorum* assembly to 16 chromosomes and found large-scale co-linearity with the *B. cinerea* genomes. Seven percent of the *S. sclerotiorum* genome comprises transposable elements compared to <1% of *B. cinerea*. The arsenal of genes associated with necrotrophic processes is similar between the species, including genes involved in plant cell wall degradation and oxalic acid production. Analysis of secondary metabolism gene clusters revealed an expansion in number and diversity of *B. cinerea*–specific secondary metabolites relative to *S. sclerotiorum*. The potential diversity in secondary metabolism might be involved in adaptation to specific ecological niches. Comparative genome analysis revealed the basis of differing sexual mating compatibility systems between *S. sclerotiorum* and *B. cinerea*. The organization of the mating-type loci differs, and their structures provide evidence for the evolution of heterothallism from homothallism. These data shed light on the evolutionary and mechanistic bases of the genetically complex traits of necrotrophic pathogenicity and sexual mating. This resource should facilitate the functional studies designed to better understand what makes these fungi such successful and persistent pathogens of agronomic crops.

## Author Summary

*Sclerotinia sclerotiorum* and *Botrytis cinerea* are notorious plant pathogenic fungi with very wide host ranges. They cause vast economic damage during crop cultivation as well as in harvested produce. These fungi are typical examples of necrotrophs: they first kill host plant cells and then colonize the dead tissue. The genome sequences of the two fungi were determined in order to examine commonalities in structure and content and in order to find unique features that may distinguish them from other pathogenic fungi and from saprotrophic fungi. The genomes show high sequence identity and a similar arrangement of genes. *S. sclerotiorum* and *B. cinerea* differ in their regulation of sexual reproduction, and the genetic basis and its evolution could be explained from the genome sequence. The genome sequence revealed a striking difference in the number and diversity of secondary metabolism gene clusters, which may be involved in the adaptation to different ecological niches. Altogether, there were no unique features in the genomes of *S. sclerotiorum* and *B. cinerea* that could be identified as “silver bullets,” which distinguish these aggressive pathogens from other pathogenic and non-pathogenic fungi. These findings reinforce the quantitative, multigenic nature of necrotrophic pathogenesis.

## Introduction

Phytopathogenic fungi have evolved a wide range of strategies to infect and colonize plants through both convergent and divergent adaptations. This is reflected in the occurrence of species within common evolutionary branches with widely diverse pathogenic lifestyles, ranging from obligate biotrophs to necrotrophs, and from host-specific to broad host range pathogens. Operationally, necrotrophs have been defined as pathogens that derive nutrients from killed host cells, biotrophs as pathogens that derive nutrients from living tissues and hemibiotrophs as pathogens that derive nutrients from a combination of feeding from living and killed host cells, respectively. The mechanisms that drive these adaptations remain largely enigmatic.

Among the few pathogens considered to be exemplary necrotrophs are the white mold fungus *Sclerotinia sclerotiorum* (Lib.) de Bary and the taxonomically closely related grey mold fungus *Botrytis cinerea* Pers. Fr. [teleomorph *Botryotinia fuckeliana* (de Bary) Whetzel]. Both fungi have considerably broader host ranges (>400 and >200 species, respectively) than most plant pathogens and each causes multi-millions of US dollars in pre- and postharvest crop losses world wide [1], [2]. Necrotrophs secrete an array of cell wall-degrading enzymes and toxins, which led to their reputation as relatively less adapted as compared to biotrophic fungi, which manipulate host physiology to obtain their nutrients from living tissues. Biotrophs are widely accepted to intimately interact and co-evolve with their hosts. Recent studies have, however, revealed that interactions between necrotrophs and their host plants are considerably more complex and subtle than previously appreciated. Some necrotrophs secrete effector proteins which are internalised by host cells and interact with the host in a gene-for-gene relationship to initiate disease, albeit in an inverse manner compared to biotrophs [3]. In the case of *S. sclerotiorum* and *B. cinerea*, the active modulation of the host redox status and the subversion of host (programmed) cell death pathways by the pathogen appear to be crucial for disease to develop [4]–[8]. The availability of molecular tools has considerably advanced our understanding of the infection strategies and pathogenic development of *S. sclerotiorum* and *B. cinerea*, yet only very few absolutely critical virulence determinants have been identified by candidate gene approaches [9].

**Figure 1:**
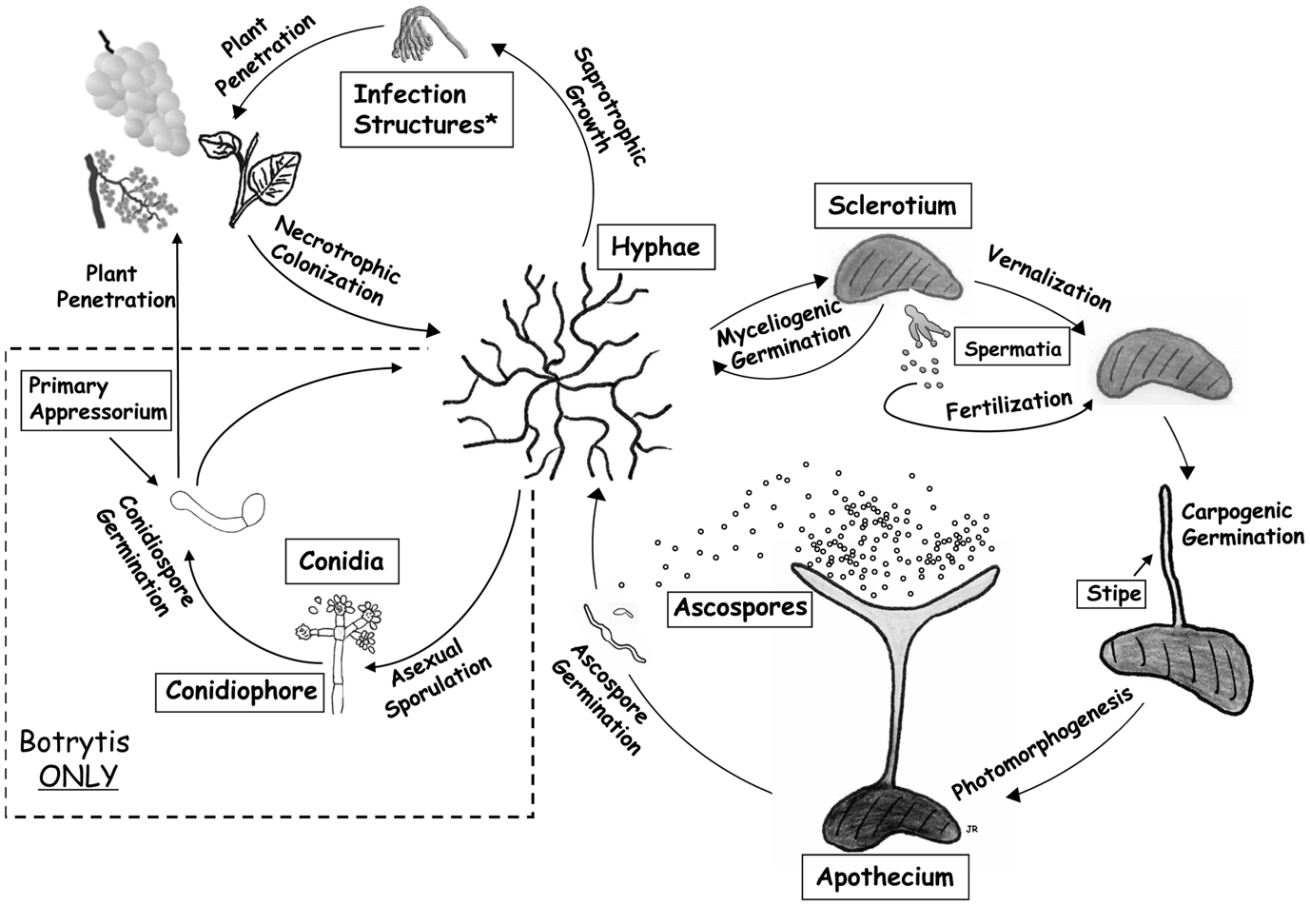
Lifecycle of *S. sclerotiorum* and *B. cinerea*, with different stages of sexual and asexual development.

Their ability to infect different plant species and tissues under a wide range of environmental conditions, as well as their ability to produce sclerotia that survive in the soil for many years, contribute to the persistent and widespread nature of these pathogens (Figure 1). The melanized sclerotium plays a central role in the lifecycle of both fungi by germinating either vegetatively for local colonization or carpogenically to initiate the sexual cycle including the production of apothecia from which ascospores are released (Figure 1). Although *S. sclerotiorum* and *B. cinerea* share many developmental and physiological features, important differences exist in their regulation and potential for sporulation. Dispersal of both species is via air-borne spores. *S. sclerotiorum* exclusively produces ascospores and not conidia (asexual spores). In contrast, *B. cinerea*, although capable of producing ascospores, is dispersed predominantly via conidia. Furthermore the regulation of sexual sporulation is quite different, *S. sclerotiorum* being homothallic (self–fertile) [1] and *B. cinerea* heterothallic (requiring a sexual partner of opposite mating type) [2]. These differences in mitotic and meiotic sporulation impact not only the life histories of these fungi but also their epidemiology and the disease control methods employed towards each.

The characteristics of *S. sclerotiorum* and *B. cinerea* pathogenicity and development stand in stark contrast to their fellow Leotiomycete powdery mildew fungi (e.g. *Blumeria*, *Erisyphe*, *Podosphaera*) which are obligate biotrophs often restricted at the species level to a single host genus. The recent description of genome sequences of two powdery mildew species [10] and two phylogenetically distant, restricted host range necrotrophs (*Phaeosphaeria nodorum* [11], *Pyrenophora teres f. teres* [12]) provides the opportunity to assess whether genomic features can be identified that are common to broad host range necrotrophs such as *B.cinerea* and *S. sclerotiorum*, yet distinct from other plant pathogenic fungi. Here we describe and compare the genome sequence assemblies and annotations for *S. sclerotiorum* and for two strains of *B. cinerea*. The comparative genome analyses of these two phytopathogenic fungi to each other, to a closely related powdery mildew and to distantly related necrotrophs offer insight into common genes underlying development and pathogenesis in *S. sclerotiorum* and *B. cinerea*, as well as genes that condition specific features of their pathogenic success.

## Results/Discussion

### Phylogenetic relationship between *S. sclerotiorum* and *B. cinerea*

*S. sclerotiorum* and *B. cinerea* are now the only fully sequenced species in the order Helotiales and with the obligate biotroph, *Blumeria graminis*, in the class Leotiomycetes of the Pezizomycotina, the largest subphylum of Ascomycota [10], [13], [14]. Within the Pezizomycotina, Leotiomycetes are most closely related to the sister lineage Sordariomycetes, and more distantly to the Eurofotiomycetes and the Dothideomycetes [14]–[16]. A phylogeny based on 82 completed fungal genomes anchors a well-supported and highly divergent Helotiales lineage including *S. sclerotiorum* and *B. cinerea* [17]. The order is, however, far too large and heterogeneous to be characterized by *S. sclerotiorum* and *B. cinerea* alone. Additional species are needed to increase the phylogenetic resolution.

**Figure 2:**
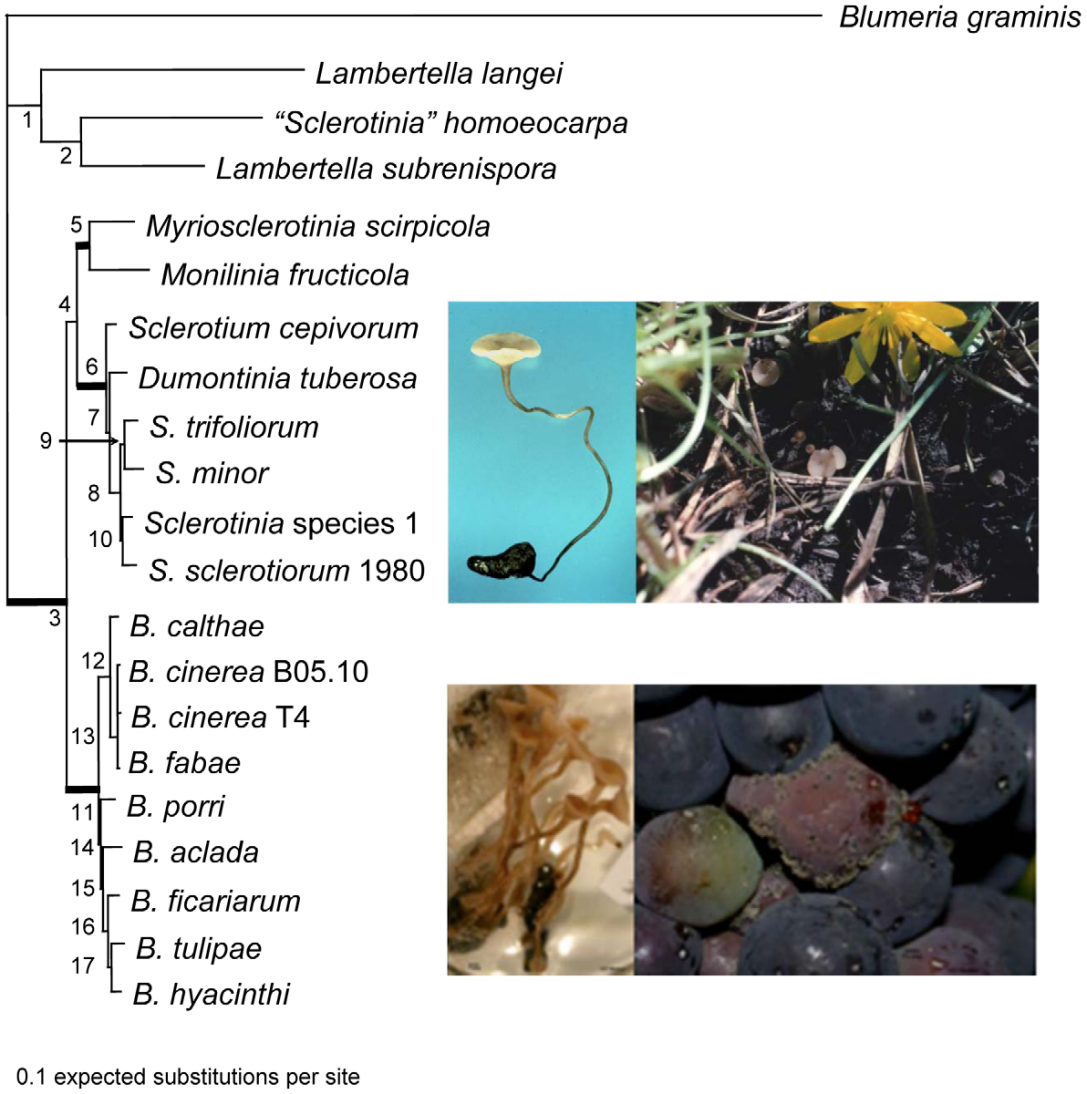
Phylogeny of the Sclerotiniaceae (Ascomycota, Leotiomycetes, Helotiales), the sister group Rutstroemiaceae (represented by *Lambertella* species and “*Sclerotinia*” *homoeocarpa*), and the outgroup, *Blumeria graminis* (Leotiomycetes, Erysiphales). The topology was estimated using Bayesian inference based on the combined sequence data of five genes. The tree was rooted using *B. graminis*. Bolded branches represent well-supported nodes with >90% support from 1000 maximum likelihood bootstrapped pseudoreplicates and >0.95 posterior probabilities. Support values for each node are listed in Table S29. Topologies recovered from single genes phylogenetic analyses were congruent with the concatenated gene tree topology. Top row *Sclerotinia sclerotiorum*, photos by H Lyon (left), LM Kohn (right). Left is apothecium emergent from sclerotium developed *in vitro*; right are apothecia associated with wild host, *Ranunculus ficaria*. Bottom row photos by AS Walker. Left is *Botryotinia fuckeliana*, sexual apothecia emergent from sclerotium developed in vitro; right, conidiophores bearing conidia produced by *Botrytis cinerea* on grapes.

We constructed a five-locus phylogeny rooted with *Blumeria graminis*, that includes two loci not previously used for this taxon sample, G3PDH and HSP60. This analysis confirms that the Sclerotiniaceae, *Sclerotinia* and *Botrytis* are closely related but distinct, monophyletic evolutionary lineages (Figure 2). This analysis also confirms that “*Sclerotinia*” *homoeocarpa*, an important pathogen of turf with morphology and etiology quite distinct from that of *Sclerotinia*, is not a *Sclerotinia* and should be reassigned to a genus in the family Rutstroemiaceae pending a reassessment of related species and generic limits. The Sclerotiniaceae includes obligate and facultative biotrophs, such as *Myriosclerotinia* species, as well as necrotrophs, as exemplified by *Sclerotinia* and *Botrytis*. *Botrytis* is divided in two sub-lineages as previously described [18]; one lineage is associated with both eudicots and monocots and the other with eudicots only. The strongly supported lineage with species of Sclerotinia on one branch, also includes the asexual *Sclerotium cepivorum*, an important pathogen of *Allium*, and a representative of the genus *Dumontinia* associated with wild plants such as *Anemone* (Ranunculaceae). Wang et al. [13] suggest that the ancestors of the lineages representing the Sclerotiniaceae and Rutstroemiaceae were associated with conifers, inferring a radiation of the Sclerotiniaceae and Rutstroemiaceae in association with the emergence and diversification of angiosperms. Co-evolution of *Botrytis* with host species has been investigated but evidence is inconclusive [18]; evidence would be concordant phylogenies between symbiont/pathogen species and host species, as demonstrated in *Monilinia* [19]. Estimates of divergence times in the phylogeny would require a molecular clock model that could be violated if some lineages have undergone accelerated evolution, as in a radiation event. Such estimates are inexact, especially when not calibrated, e.g., by fossil evidence.

### Genome organization

#### General genome features

The genomes of *S. sclerotiorum* strain 1980 and *B. cinerea* strains T4 and B05.10 were sequenced using Sanger technology. High coverage (9.1X and 10X respectively) was generated for *S. sclerotiorum* 1980 and *B. cinerea* T4, and a lower coverage (4.5X) for *B. cinerea* B05.10 (Table 1). All sequences were assembled using Arachne [20] to generate a consensus for each genome. The three genomes are similar in size, ranging from 37.9 Mb to 38.8 Mb (Table 1). The slightly larger genome size for *B. cinerea* B05.10 (38.8 Mb) is likely inflated due to the lower sequence coverage, as small scaffolds may fall into larger scaffold gaps. To assess the accuracy and completeness of the *S. sclerotiorum* assembly, we generated an optical map, and aligned the 34 largest of the 36 assembly scaffolds to the optical map based on shared restriction sites (Figure S1). The map consists of 16 linkage groups which likely correspond to the estimation of 16 chromofsomes from pulsed-field gels of *S. sclerotiorum* chromosomes [21]. Microscopy in several *Botrytis* species, including *B. cinerea*, has estimated 16 chromosomes [22]. The total *S. sclerotiorum* optical map size is estimated at 39.6 Mb. Since 38.0 Mb is covered by scaffolds, approximately 1.6 Mb of sequence is missing from the assembly of *S. sclerotiorum*. Most of the uncovered regions of the map are located in the middle of chromosomes, which may correspond to centromeres (Figure S1). In many filamentous fungi, centromeres consist of highly repetitive sequences which can be refractory to cloning and therefore sequencing [23]. By contrast, most chromosomes are fully covered at their telomeric ends, with 28 of 32 chromosome ends being linked to telomeric repeats. One chromosome (chrR) ends in ribosomal DNA tandem repeats. The assembly of *B. cinerea* T4 was verified by generating a genetic map containing 134 polymorphic microsatellite markers and 62 SNPs, using 68 progeny from a cross (Text S1). The total length of scaffolds appearing in the genetic map (Table S1) covered 31.8 Mbp, representing 80% of the T4 sequence assembly. Two scaffolds were in conflict with the genetic map.

**Table 1:** Assembly and gene statistics.

	*S. sclerotiorum*	*B. cinerea*	
	1980	T4	B05.10
Coverage	9.1X	10X	4.5X
Assembly size (Mb)	38.3	39.5	42.3
Total contig length (Mb)	38.0	37.9	38.8
Scaffolds	36	118	588
Scaffold N50 (kb)	1,630	562	257
Contigs	679	2,281	4,534
Contig N50 (kb)	123	35	16.4
≥Q40 (%)	98.7	98.7	98.0
GC (%)	41.8	43.2	43.1
Predicted protein-coding genes	14,522	16,360	16,448
Dubious genes	2,662	2,090	2,784
High-confidence genes	11,860	14,270	13,664
Median coding sequence length (nt)	813	807	744
Median exon length (nt)	182	208	190
Median intron length	78	62	74
Median intergenic length (nt)	973	778	958
GC Exonic (%)	45.7	46.2	46.2
GC Intronic (%)	39.9	40.0	40.9
tRNAs	191	200	195
Transposable elements	7%	0.7%	0.9%

The average GC contents of *S. sclerotiorum* and *B. cinerea* genome sequences (41.8–43.2%) are comparable to *Blumeria graminis* (44%; [10]), but significantly lower than those from related fungi (50–52%, Pezizomycotina, Figure S2). Exon sequences in *S. sclerotiorum* and *B. cinerea* are 6% higher in GC content than introns (Table 1). GC% does not vary along contigs or chromosomes, and shows no evidence of AT-rich isochores, such as observed in *Saccharomyces cerevisiae* [24] or *Leptosphaeria maculans* [25], nor is it due to a bimodal distribution as observed in *Neurospora crassa* (Figure S2). The evenly distributed low GC content of Leotiomycetes, which distinguishes them from most other Pezizomycetes, may influence chromosome organization and steady state transcript levels [26].

The number of genes predicted in *S. sclerotiorum* strain 1980 (14,522 genes) and *B. cinerea* (16,448 and 16,360 genes for strains B05.10 and T4, respectively) is larger than those of related fungi (average 11,154 genes, Table S2). This discrepancy is mainly due to the large number (*S. sclerotiorum*: 3,461; *B. cinerea* B05.10: 3,975; *B. cinerea* T4: 4,229) of small predicted proteins less than 100 amino acids in length. Based on these observations, we revised the annotation process to flag small proteins without evidence as dubious. In order to be annotated, small proteins were required to show evidence of expression (ESTs and microarray signals), function (containing known domains) or evolutionary conservation (existence of orthologs or paralogs) (Figure S3). This resulted in a number of high confidence predicted proteins (*S. sclerotiorum*: 11,860; *B. cinerea* B05.10: 13,664; *B. cinerea* T4: 14,270), comparable to that of other fungal genomes (Figure S4 and Table S2).

#### Large synteny blocks are shared by *S. sclerotiorum* and *B. cinerea*

**Figure 3:**
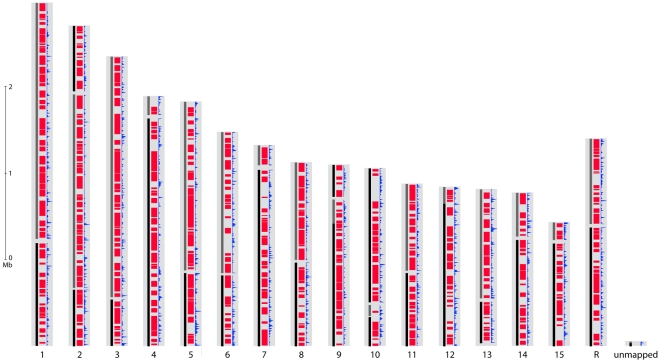
Genome organization of *S. sclerotiorum*. For each putative chromosome of the optical map, alignment of supercontigs is shown in alternating color blocks of black and grey. Syntenic regions with *B. cinerea* T4 are shown in red. Frequency of repetitive sequences is shown in blue.

The gene sets of *S. sclerotiorum* and *B. cinerea* are highly similar with a total of 8,609 Bidirectional Best BLAST Hits (BDBHs) between *S. sclerotiorum* and *B. cinerea* B05.10 and 8,601 between *S. sclerotiorum* and *B. cinerea* T4. The protein sequences deduced from these pairs of BDBH genes have a median identity of 84.0%. A total of 19% of the *S. sclerotiorum* genome can be aligned at the nucleotide level to both *B. cinerea* genomes and 46% at the protein level. By contrast, nearly all of the two *B. cinerea* genomes can be aligned to each other, and these genomes share an average of 99.2% identity at the nucleotide level. To build syntenic regions between genomes, we used DAGchainer [27] to identify blocks of four or more orthologous BDBH gene pairs. Syntenic regions include 7,752 *S. sclerotiorum* genes with orthologs in *B. cinerea*; these regions also cover 3,618 unpaired genes from *S. sclerotiorum* that are enriched for dubious genes (2-fold more than the full genomes). Across all syntenic regions, the *S. sclerotiorum* genome shares 27.7 Mb with the *B. cinerea* T4 genome (Figure 3, Figure S5) and a similar amount with the B05.10 genome. Syntenic regions are distributed evenly across *S. sclerotiorum* chromosomes, including subtelomeric regions; some synteny breakpoints are marked by an increased density of repetitive elements in *S. sclerotiorum* (Figure 3). Further larger scale analysis of synteny requires anchoring the *B. cinerea* sequence onto chromosomes, using a genetic or physical map.

We also examined each of the genomes for segmental duplication blocks. The *S. sclerotiorum* genome contains 8 duplicated blocks ranging from 4 to 12 paired syntenic genes; only 2 duplicated blocks were identified in the *B. cinerea* B05.10 genome, and none in the *B. cinerea* T4 genome (Table S3). Of the 8 blocks in *S. sclerotiorum*, 4 contain genes encoding proteins similar to the heterokaryon incompatibility proteins HET-E-1 of *Podospora anserina* [28]. Most HET-E-1 homologs in *S. sclerotiorum* fall into a single gene family; in this family *S. sclerotiorum* has twice as many HET-E-1 domain containing genes as that of *P. anserina*, and three fold more than other fungi including *B. cinerea*. While duplicated blocks in B05.10 did not contain HET-like proteins, a different family of proteins with a HET domain (PF06985) is expanded in *B. cinerea*, with a total number of 65 or 79 in B05.10 or T4, respectively, compared to 41 in *S. sclerotiorum*. The recent expansion of two HET-domain containing families in both fungi suggests that these may have been involved in speciation.

#### Expansion of transposons in the *S. sclerotiorum* genofome

**Figure 4:**
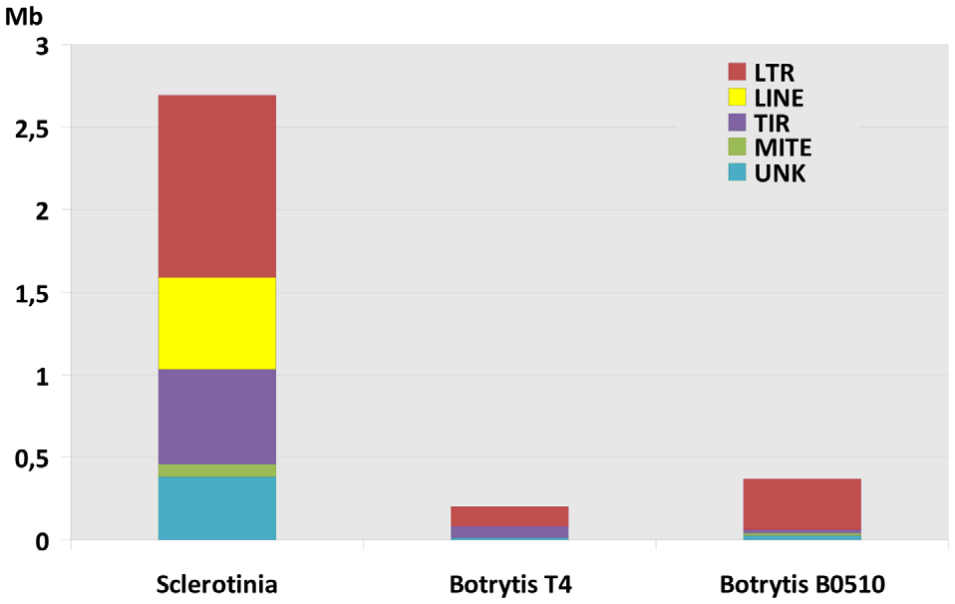
Transposable element content of the genomes of *S. sclerotiorum* and *B.cinerea*. Distribution of transposable elements in the genomes of *S. sclerotiorum* and *B. cinerea* (T4 and B05.10 isolates) according to the major clades: LTR retro-transposons, Line retro-transposons, TIR DNA transposons, MITE. UNK refers to unclassified transposable elements.

Repetitive sequences were first identified by self-alignment of each genome using cross match, revealing 7.7% of the *S. sclerotiorum* genome as repeats, as compared to 4.4% for *B. cinerea* B05.10 and 3.3% for *B. cinerea* T4 (Tables S4,S5). This *de novo* search identified 500 evenly distributed copies of a 300 bp element derived from the IGS region of the rDNA specific to *S. sclerotiorum*. rDNA derived repeats (>150 copies) have recently been described in the *L. maculans* genome [25], however such elements are more frequently found in plants [29]. Using the REPET pipeline [30], [31] and extensive manual annotation, transposable elements (TEs) were identified in *S. sclerotiorum* and *B. cinerea* genomes, grouped into families and annotated as either class I (LTR, LINE), class II (MITE, TIR) or unknown TEs (Table S6). They occupy 7% of the *S. sclerotiorum* genome and 0.6 to 0.9% of the *B. cinerea* genomes (T4, B05.10) respectively (Figure 4). Therefore, *S. sclerotiorum* has a 10-fold higher TE content than *B. cinerea* that is associated with an increase in the number of TE families (4-fold, N: 41) and the total number of TE copies (12-fold, N: 4143, Table S6). Moreover, *S. sclerotiorum* contains five families of high copy number LINE elements, which are not present in *B. cinerea* genomes (Table S6).

Despite the higher TE content of *S. sclerotiorum*, its genome size is similar to that of *B. cinerea*. This strongly contrasts with the related Leotiomycete *B. graminis*, which has a genome size of 130 Mb and a TE content of 64% [10]. We hypothesize that *S. sclerotiorum* has mechanisms to reduce the effects of TE expansion on genome organization. One such mechanism involves a reduction in TE length, as observed for *S. sclerotiorum* Gypsy and Copia retro-elements (Table S7) that are mainly composed of solo-LTRs (N: 2209; average ratio solo-LTRs/full-length copies: 42) scattered in the genome. Similar TEs in *B. cinerea* have a 20-fold lower proportion of solo-LTRs/full-length copies (average ratio 2; Table S7). Solo-LTRs result from recombination between LTRs of ancestral TE copies [32]. Since they represent only 1/10th of the length of intact retro-elements (average 5000 bp), the reduction in size from full-length retro-elements to solo-LTRs could have prevented an increase in genome size of 12 Mb (30% of the actual genome size), which would be expected if the *S. sclerotiorum* genome included 2,209 additional full-length retro-elements. The potential deleterious effect on the genome of such a large number of TEs appears to have been limited by truncating the majority of elements in the expanded retroelement families.

TEs in *S. sclerotiorum* are less diverse than those in *B. cinerea*. This is illustrated by the Mariner ScTIR1 family, which has a lower genetic diversity (θ: 0.12) including a subset of quasi-identical copies, as compared to the BcTIR1 family (θ: 0.24). Furthermore, ScTIR1 has 5 subfamilies consisting of 3 to 15 copies with identical sequences that are dispersed in the genome. Phylogenetic analysis suggests that a significant proportion of *S. sclerotiorum* TEs result from recent transposition events (Figure S6). These analyses highlight that the *S. sclerotiorum* genome experienced a profound, recent remodeling associated with a dramatic expansion of TEs. This recent evolution of the *S. sclerotiorum* genome is responsible for important differences in genome organization compared to *B. cinerea*. Indeed, loss of synteny between the two genomes is frequently associated with the presence of repeats at synteny break points (Figure 3). The TE expansion in *S. sclerotiorum* might also impact on its genome organization and functioning (gene inactivation, modification or regulation of expression).

### Gene content

#### Gene conservation and evolution

We compared *S. sclerotiorum* and *B. cinerea* to a set of seven other genomes which include a biotrophic Leotiomycete (*B. graminis*), two non-related necrotrophs (*Phaeosphaeria nodorum* and *P. teres*), two hemi-biotrophic model plant pathogens (*Magnaporthe oryzae* and *Gibberella zeae*) and two saprobes (*N. crassa* and *Aspergillus niger*). About 8,200 genes from *S. sclerotiorum* and *B. cinerea* have homologs in other fungi, of which about 3,500 are conserved in all compared fungal genomes (Table S8). Both *S. sclerotiorum* and *B. cinerea* harbor fewer multigene families as compared to *G. zeae*, *A. niger* or *P. nodorum* but similar numbers as *M. oryzae* (Figure S7). Around 10% of the *S. sclerotiorum* and *B. cinerea* genes (1,454–1,600) are clade-specific, *i.e.* they are shared exclusively among the two species and have no orthologs in other fungi (Table S8). Nearly 90% of these clade-specific genes encode proteins without known PFAM domains; the subset of genes with functional domains is enriched in GO terms associated with transcription factors and cytochrome P450s (Table S9). After excluding dubious genes, the number of species-specific (either to *S. sclerotiorum* or to *B. cinerea*) genes was comparable to that in other fungal genomes (approximately 2,600). Many of these genes encode small proteins without functional domains. The species-specific genes harboring functional domains are enriched in genes encoding cytochrome P450s, transcription factors, and proteins with domains involved in chromatin remodeling (Table S9).

To more closely examine patterns of gain and loss of larger functional classes in *S. sclerotiorum* and *B. cinerea*, we examined PFAM domain content across these ten genomes. Two families of glycosyl hydrolase, GH71 and GH28, were the most enriched in *S. sclerotiorum* and *B. cinerea* (Table S10). These genomes are also enriched for domains of several enzymes, including a putative amidoligase enzyme, a GMC oxidoreductase, and a GDP_fucose protein O-fucosyltransferase. The other Leotiomycete included in these comparisons, *B. graminis*, does not contain similar expansions, and calculating enrichment for all Leotiomycetes identified no additional domains. As *B. graminis* contains only 5,495 predicted proteins, other less reduced Leotiomycetes may provide better points of comparison when sequenced. Extending the comparison to search for domains enriched in all necrotrophic fungi (including *P. nodorum* and *P. teres*) identified only one additional enriched domain, that for the Heterokaryon incompatibility protein. By contrast, conserved genes depleted in *S. sclerotiorum* and *B. cinerea* include a larger and more diverse set than the enriched. These include several families addressed further in this paper, the glycosyl hydrolases GH2 and GH43, the subtilase family (which is most prominent in *M. oryzae* and *N. crassa*), zinc finger domains associated with transcription factors, and a terpene synthase domain (Table S11).

#### Genes in primary metabolism

Conserved primary metabolism genes for amino acid synthesis and for mitochondrial function are present in both genomes, few features deviate from other fungi. *S. sclerotiorum* and *B. cinerea* contain five genes encoding alternative type II NADH dehydrogenases, while two or three such genes are found in most fungi, and four in *N. crassa* [33]. Components of the oxidative phosphorylation system are thus present in *S. sclerotiorum* and *B. cinerea* in a higher number than in most fungi. These genes may contribute to plasticity, as suggested for *N. crassa* [34], increasing fungal versatility in modulating energy production and the cellular redox status in response to environmental stimuli. This may be relevant for *S. sclerotiorum* and *B. cinerea* in view of their capacity of redox modulation by production of oxalic acid and the induction of ROS during infection (see section: ROS generation and tolerance).

#### Genes involved in signaling pathways

All types of genes that are involved in signalling pathways in filamentous fungi (G protein-coupled receptors, MAP kinases, heterotrimeric G proteins, cAMP signalling components and Ca^2+^-related signalling) are found in *S. sclerotiorum* and *B. cinerea* (Table S12). Few differences with other fungi were observed. One GPCR gene in *S. sclerotiorum* (SS1G_07511) and *B. cinerea* (BofuT4_P129750.1, BC1G_05052) is absent in *N. crassa*, but present in *Aspergillus nidulans* (GprC) and *M. oryzae* (MGG_08803). *S. sclerotiorum* and *B. cinerea* each contain two phospholipase C genes [35], while in *M. oryzae* and *N. crassa* there are four. *S. sclerotiorum* and *B. cinerea* each have two calmodulin genes while in *M. oryzae* and *N. crassa* there is only one. Moreover, *S. sclerotiorum* and *B. cinerea* contain an additional calmodulin-like gene (SS1G_05131, BofuT4_P159960.1, BC1G_11227), similar to those in plants, but which is not present in other fungal genomes. Another exception that stands out to the high degree of conservation is that of the *S. sclerotiorum* low MW Tyr phosphatase (SS1G_04959.1), which is highly similar to other LMW phosphatases, however it lacks the conserved C(X)5R(S/T) motif considered to be a cross-kingdom consensus for this class of phosphatases. The *B. cinerea* homolog (BC1G_14690.1) does contain the consensus motif. Whether this unique *S. sclerotiorum* phosphatase is functional has yet to be determined.

The group of genes encoding sensor histidine kinases (HKs) shows a high diversity in the number of members and domain structures. In general, filamentous fungi have a larger complement of HKs than yeast (*S. cerevisiae* has one, *Schizosaccharomyces pombe* has three). *N. crassa* has eleven HK-encoding genes while both *S. sclerotiorum* and *B. cinerea* possess twenty HK-encoding genes. Similar numbers of HK-encoding genes are found in *Cochliobolus heterostrophus* (21) and *Gibberella moniliformis* (16) [36]. Interestingly, group VIII of fungal HKs has been expanded in *S. sclerotiorum* and *B. cinerea*; it has only one representative in *A. nidulans* and *M. oryzae*, two in *N. crassa* but three (PHY1–PHY3) in *S. sclerotiorum* and *B. cinerea*. Members of this group contain domains common to plant phytochromes and represent putative red/far-red light sensors. Whether PHY1–PHY3 have a role in the life cycle of *S. sclerotiorum* and *B. cinerea* remains to be studied.

Even though the components of different signal transduction pathways are conserved among fungi, their activation by an external stimulus, their interconnections with other signalling components and their outputs vary significantly. For instance, the cAMP cascade regulates penetration and pathogenicity in appressoria-forming fungi [37]. In *S. sclerotiorum*, the cAMP cascade regulates infection cushion development and is essential for pathogenicity [38], while the same pathway plays a minor role in virulence of *B. cinerea* [39], [40]. Besides the cAMP cascade, MAPK modules, small GTPases and the Ca^2+^-calmodulin pathway have been studied in *B. cinerea* to obtain insight in their role in pathogenicity (reviewed by [9]).

#### Genes involved in programmed cell death in the pathogen

Programmed cell death (PCD), often referred to as apoptosis, is an important cellular process in eukaryotic organisms [41]. PCD in plants is important in conferring resistance to biotrophic plant pathogens during a hypersensitive response [42]. Conversely, the ability of necrotrophic pathogens to induce PCD in plants is crucial for their ability to infect their host [4], [43], [44]. However, the occurrence of PCD in fungal pathogens may also be important for their ability to infect plants, as demonstrated in *M. oryzae* [45], [46]. We explored the genomes of *S. sclerotiorum* and *B. cinerea* for genes involved in the execution or regulation of apoptosis. Both fungi contain homologs of apoptosis-associated genes (Table S13), and the encoded proteins share features with proteins in *S. cerevisiae*. However, in several cases they also share domains with human orthologs, which are not always present in the yeast counterpart. For example, *S. cerevisiae* Nma111p is a homolog of the human pro-apoptotic protein Omi/HrtA2, which contains an IAP-binding domain, a single PDZ domain, a trimerization motif and a mitochondrial localization signal. *S. cerevisiae* Nma111p has none of these features except for two short PDZ domains. The *S. sclerotiorum* and *B. cinerea* homologs are highly similar to each other and show greater sequence similarity to the *S. cerevisiae* than to the human protein. However, alike the human Omi/HtrA2, they have an IAP binding domain and a short, single PDZ domain [47]. This and similar findings are typical of the situation in many filamentous ascomycetes, suggesting that apoptotic networks in filamentous fungi might represent an evolutionary intermediate between budding yeasts and higher eukaryotes.

The phylogeny of apoptotic genes in fungi reveals close relatedness to higher eukaryotes, however some important components of the animal apoptosis network are absent in fungi. In particular there are no homologs of Bcl-2 or death receptors, both crucial apoptosis regulatory proteins in animals. A third important group are the ‘Inhibitor of Apoptosis Proteins’ (IAPs). There are eight IAP members in human, all of which contain 1 to 3 BIR (Baculovirus IAP Repeat) domains that are necessary for their anti-apoptotic activity. A single protein with two BIR domains is found in *S. cerevisiae* (Bir1p) and homologs of Bir1p are found in most fungi (Figure S8), including *S. sclerotiorum* and *B. cinerea*. Cross-kingdom analysis shows that BIR-domain containing proteins are only present in fungi and metazoans (not shown), suggesting that this domain first appeared in their common ancestor. The *B. cinerea* homolog (BcBir1; BC1G_14521.1) is anti-apoptotic, and its activity requires the two BIR domains [48]. Recent studies indicate that the BcBir1-mediated anti-apoptotic response is important for virulence of *B. cinerea* [49]. Functional analysis of candidate apoptosis-associated genes in *S. sclerotiorum* and *B. cinerea* genomes will elucidate their role in pathogenesis.

#### *B. cinerea* is heterogeneous for the presence of mobile intein elements

Inteins (*in*ternal pro*tein*s) are in-frame protein insertions embedded within other proteins, which are transcribed and translated with their host gene [50]. They are excised from protein precursors in a post-translational autocatalytic event, which forms a *de novo* peptide bond between the flanking extein (*ex*ternal pro*tein*) sequences. In addition to protein splicing domains, most inteins have a homing endonuclease domain (HEG), which facilitates their specific ‘homing’ into intein-less homologs by gene conversion. PRP8 inteins do not frequently occur in fungal genomes, but have been identified in various orders, i.e. Eurotiales, Onygenales, Sordariales, Chytridiales and Mucorales [50]. The genome sequence of *B. cinerea* strain B05.10 contains an intein element incorporated in the *Prp8* gene (BC1G_06754/06755). The BciPRP8 intein element is 2,514 bp in length and encodes the N- and C-terminal splicing domains and the HEG domain. There is no intein in the *Prp8* gene of *B. cinerea* strain T4, and the ‘empty’ *Prp8* allele of T4 is identical to ‘empty’ alleles of other *B. cinerea* isolates. Heterogeneity for the presence or absence of an intein within a species is unique in filamentous fungi and was exploited to show the occurrence of gene conversion (‘homing’) at 100% efficiency, during meiosis in crosses between *B. cinerea* isolates carrying an intein and isolates with an empty *Prp8* allele [51]. *S. sclerotiorum* (strain 1980 and 14 other strains) does not contain inteins, however analysis of the intein-extein junction region revealed that the ancestral *S. sclerotiorum Prp8* gene contained an intein which was lost by precise deletion, leaving a footprint of previous occupation (Bokor et al., in preparation).

### Genes involved in development

#### Genes involved in mating and fruiting body development

Genome sequencing of *S. sclerotiorum* and *B. cinerea* offers opportunities to gain insights into novel aspects of fungal mating and multicellular development (Figure 1). In particular, both species produce macroscopic, stipitate apothecia (sexual fruiting bodies), which arise from within the tissues of melanized, sclerotial stroma. This form of fungal sexual development has not previously been subjected to genome analysis. Also, sexual behaviour differs fundamentally between the species. Whereas *S. sclerotiorum* is homothallic (self fertile) and only occasionally outcrosses [52], *B. cinerea* is considered a heterothallic (obligate outcrossing) species with two mating types, MAT1-1 and MAT1-2 [53]. Genome analysis might explain the basis of such sexual differences.

**Figure 5:**
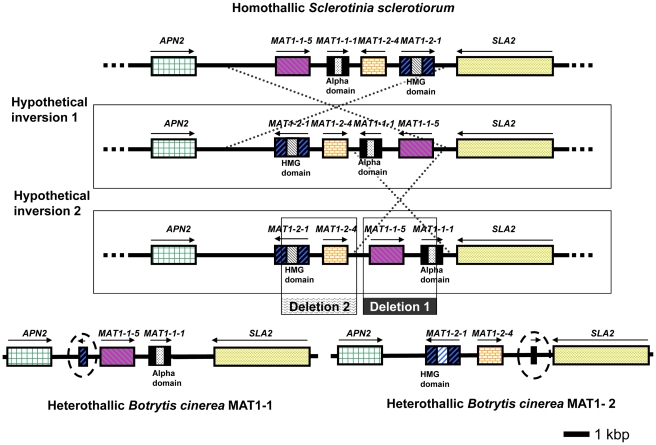
Configuration of the MAT loci in *S. sclerotiorum* and *B. cinerea* (strains B05.10 and T4). *S. sclerotiorum* is homothallic whereas *B. cinerea* strains are heterothallic. Strain B05.10 is of MAT1-1 identity whereas strain T4 is of MAT1-2 identity. Orthologous genes are displayed in the same colour and pattern. The entire *MAT* locus is contained between the genes *APN2* (on the left, green) and *SLA2* (on the right, yellow stippled). The *MAT* locus of *S. sclerotiorum* is displayed on the top line, whereas the *MAT* loci of both *B. cinerea* strains are displayed on the bottom line. The truncated fragments of the *MAT1-1-2* gene in the MAT1-1 isolate and of the *MAT1-1-1* gene in the MAT1-2 isolate are highlighted with a dotted circle. Possible ancestral loci are displayed in the middle. Gene names are indicated above the gene model, the presence of a conserved alpha domain or HMG domain is indicated below the gene model. Two hypothetical inversions are shown, which might convert one configuration into the other. Two separate deletions are shown which may have resulted in the evolution of the MAT1-1 or MAT1-2 locus in *B. cinerea*.

Previous investigations of Pezizomycete fungi have established that differences in breeding systems are normally determined by the presence of different arrangements of mating-type (*MAT*) genes at one or more *MAT* loci [54]. *MAT* genes encode transcription factors required for sexual development [54]. Analysis of the present genomes indeed revealed differences in configuration of *MAT* loci between the two species (Figure 5). *S. sclerotiorum* has an organization typical of homothallic Pezizomycotina, with a single *MAT* locus containing both alpha- and high mobility group (HMG)-domain encoding *MAT* genes (SS1G_04004.1 and SS1G_04006.1). Only one HMG-type gene is present, supporting the hypothesis that the ancestral fungal *MAT* locus contained a single HMG gene [55], [56]. Meanwhile, the genomes of the two sequenced *B. cinerea* strains show the typical *MAT* organization of heterothallic Pezizomycotina, with the presence of dissimilar ‘idiomorphs’ at a single *MAT* locus [54]. Isolate B05.10 (mating type MAT1-1) contains a *MAT1-1* idiomorph including a characteristic *MAT1-1-1* alpha-domain gene (Bc1G_15148.1), whereas isolate T4 (mating type MAT1-2) contains a *MAT1-2* idiomorph including a characteristic *MAT1-2-1* HMG-domain gene (BofuT4_P160320.1).

However, the *S. sclerotiorum* and *B. cinerea MAT* loci were also found to have unusual features. First, two novel ORFs not previously reported from other fungi were detected. The first, designated *MAT1-1-5* [54], is present in the *MAT1-1* idiomorph of *B. cinerea* B05.10 (Bc1G_15147.1) with a homolog in the *S. sclerotiorum MAT* locus (SS1G_04003.1), but is absent from the *MAT1-2* idiomorph of *B. cinerea* T4 (Figure 5). The second ORF, named *MAT1-2-4*, is present in the *MAT1-2* idiomorph of *B. cinerea* T4 (BofuT4_P160330.1) with a homolog in the *S. sclerotiorum MAT* locus (SS1G_04005.1), but is absent from the *MAT1-1* idiomorph of *B. cinerea* B05.10 (Figure 5). Novel genes have also been reported in *MAT* loci of other ascomycete fungi [57]–[60]. *MAT* regions were sequenced from seven additional *S. sclerotiorum* and two *B. cinerea* isolates to confirm these results were typical. *S. sclerotiorum* strain 1980 originates from a population with little evidence of genetic recombination. Sequencing of the *MAT* region from the additional *S. sclerotiorum* isolates, drawn from populations showing evidence of recombination, revealed the same *MAT* genes to be present, although with minor sequence differences (2–4 haplotypes for each gene). *MAT* region sequences from *B. cinerea* tester strains SAS56 (MAT1-1) and SAS405 (MAT1-2) [53] were very similar to B05.10 and T4, respectively.

A second unusual feature concerned the *B. cinerea MAT* loci. Fragments of *MAT1-2-1* and *MAT1-1-1* genes bordered the idiomorphs of B05.10 (MAT1-1) and T4 (MAT1-2), respectively (Figure 5). Both fragments appear non-functional, lacking start codons and a *MAT* domain. In typical heterothallic ascomycetes the *MAT* regions contain either *MAT1-1* or *MAT1-2* sequence, but not fragments of the *MAT* gene(s) of the opposite mating type. In some exceptional cases a complementary *MAT* fragment was present in solely one mating type (e.g. [61], [62]). *B. cinerea* is unique in having redundant fragments bordering both idiomorphs. This configuration is consistent with *B. cinerea* having evolved from a homothallic ancestor containing complete *MAT1-1-1* and *MAT1-2-1* genes at the same locus, with MAT1-1 and MAT1-2 mating types arising from the loss of core HMG and alpha-domain sequences, respectively, leaving only the disabled gene fragments seen in the current *MAT* loci (Figure 5). Indeed, the configurations of the *B. cinerea MAT* loci can be explained by the occurrence of two simple inversion and deletion events from a *S. sclerotiorum*-like homothallic ancestor (Figure 5). Such rearrangements have been observed in other *MAT* loci [57]. There are reports of the evolution of homothallism from heterothallism in nature based on rearrangements at the *MAT* locus [63], [64]; the current data provides the first clear evidence for the opposite transition from homothallism to heterothallism.

Finally, *B. cinerea* is unusual among pezizomycete fungi in that some isolates are capable of ‘dual mating’. Whereas most isolates act in a standard heterothallic fashion, occasionally isolates are detected that can mate with both MAT1-1 and MAT1-2 tester strains [53], [54], [65]. The organization of *MAT* loci in dual-mater strains of *B. cinerea* was investigated (Text S2). Four dual-mater isolates exhibited a *MAT1-2* like locus, and one contained a *MAT1-1* like locus. Neither PCR analysis nor Southern hybridization revealed any sequence homologous to the opposite *MAT* locus. We conclude that dual mating in *B. cinerea* is not due to the possession of both *MAT1-1* and *MAT1-2* sequence in the same genome. Instead this phenomenon arises by changes elsewhere in the genome - it is unknown whether this form of mating-type switching has a monogenic or polygenic basis.

Mating-type, and a series of other ‘sex-related’, genes have been identified as being involved in various stages of mating and fruiting body production in the Pezizomycotina but these genes have been identified primarily from studies concerning development of cleistothecia or perithecia [54], [66] rather than the apothecia seen in the Leotiomycotina. Screening of the genomes of *S. sclerotiorum* and *B. cinerea* with genes with known roles in mating and fruiting body development [66], [67] revealed orthologs for every gene tested (Table S14). This indicates that mating processes (e.g. pheromone signalling) and physiological factors governing asexual and sexual development (e.g. oxylipin signalling) are largely conserved within the Pezizomycotina. Given that the Leotiomycetes are a phylogenetic sister group of the Sordariomycetes, availability of genome sequence now makes it possible to compare how genes are spatially and temporally regulated and what novel genes give rise to tissue patterns distinctive of cleistothecia, perithecia and apothecia.

#### Genes involved in sclerotial development

Genes associated with sclerotial development have been described previously [68], including genes encoding the pH-responsive transcription factor Pac1, Ras, protein phosphatases, the MAP Kinases Smk1 and Bmp1 and adenylate cyclases Sac1 and Bac1. All of these proteins are components of signal transduction pathways and have pleiotropic effects on growth and development. Genes that are associated exclusively with sclerotial development may lend greater insight into the makeup, regulation and evolution of sclerotia. One such protein (Ssp1) was described originally as a sclerotium-specific protein [69] and accumulates to the highest abundance of any soluble protein in sclerotia, comprising nearly 50% of total soluble protein in a mature sclerotium. The *ssp1* gene (SS1G_14065.1) was cloned [70] and characterized by deletion mutagenesis [71]. *ssp1* transcripts accumulate only in sclerotial tissues making this a valuable gene for studying the regulation of sclerotium-specific gene expression. The search for related genes in other fungi outside the Sclerotiniaceae has revealed *ssp1* homologs only in *Aspergillus flavus* and *A. oryzae*, distantly-related Eurotiomycetes that share with *S. sclerotiorum* and *B. cinerea*, the ability to produce sclerotia. Using Reciprocal Smallest Distance analysis [72], 245 orthologous gene pairs shared by *S. sclerotiorum*, *B. cinerea*, *A. oryzae* and *A. flavus* but absent from the non-sclerotia-producing *A. nidulans* and *A. fumigatus* have been identified (Table S15). This list includes *ssp1* and may be enriched in sclerotium-specific genes offering a potential foothold into a broader understanding of sclerotium development and evolution.

#### Genes involved in conidiation

A developmental feature that distinguishes *B. cinerea* from *S. sclerotiorum* is conidiation. Both species produce spermatia (microconidia) from phialides arising singly or in clusters directly from basal hyphae. *B. cinerea* and other *Botrytis* species, however, have the ability to produce macroconidia from sterigmata on the swollen tips of aerial, branching conidiophores. *Sclerotinia* spp. do not produce structures resembling conidiophores or macroconidia. Genes dictating spatial patterning of sporulation might be present in *B. cinerea* but absent from *S. sclerotiorum*. Conidiophore pattern and cell-identity regulators in *A. nidulans* include *medusa* (*medA*), *stunted* (*stuA*), *abacus* (*abaA*) and *bristle* (*brlA*). Orthologs of *medA*, *stuA*, and *abaA* are present in *B. cinerea* and *S. sclerotiorum* whereas an unambiguous ortholog of *brlA* is not present in either genome (Table S16), as is also the case in *N. crassa* [73]. However, genes known to function upstream of *brlA* including *fadA*, *fluG*, *flbC*, and *flbD* are all present in both *B. cinerea* and *S. sclerotiorum* (Table S16). Neither *B. cinerea* nor *S. sclerotiorum* contains an ortholog to the *N. crassa fluffy* gene (NCU08726.3). Thus, comparative genomics fails to reveal a simple explanation for asexual sporulation in *B. cinerea* and its absence in *S. sclerotiorum*. However, there is no evidence that the *B. cinerea* orthologs of *Aspergillus* sporulation genes also function in sporulation in *B. cinerea*. The orthologs in *S. sclerotiorum* may have evolved to perhaps regulate the microconidiation pathway (shared between *S. sclerotiorum* and *B. cinerea*) or to regulate different processes. Functional analyses will be required to determine the role(s) of these genes in *S. sclerotiorum* and *B. cinerea* biology.

#### Genes involved in infection structure development

In addition to structures involved in sexual and asexual development unrelated to pathogenesis, *B. cinerea* and *S. sclerotiorum* both produce dedicated infection structures in order to invade plants. Both fungi demonstrate plasticity in the complexity of these structures used to breach the host cuticle. The structures range from simple swellings of germ tube tips and dome-shaped appressoria lacking melanin in their cell walls, to multi-lobed compound appressoria and the more complex infection cushions. Many of the genes that affect appressorium development in *M. oryzae* and other plant pathogenic fungi are components of the highly conserved MAP kinase and cAMP-dependent signal transduction pathways. Orthologs of all these genes are present in both *S. sclerotiorum* and *B. cinerea*, as well as in non-pathogenic fungi (Table S12). The tetraspanin-encoding gene (*pls1*) that is required for appressorium function in both *M. oryzae* and *B. cinerea* [74], is also present in *S. sclerotiorum* (Table S17). Among appressorium-associated genes (Table S17) are also members of the *mas1*-related gene family, which play a role in *M. oryzae* appressorium function [75]. Two members of the *mas1* family from *S. sclerotiorum* were over-represented in EST sequences from an infection cushion cDNA library [76]. *mas1*/*mas2* homologs in *B. cinerea* are upregulated during germination and appressorium formation, but the genes are not essential for infection [77]. Identifying specific genes that are essential for appressorium development as well as other developmental stages such as sclerotia and apothecia could provide targets for controlling disease spread through lifecycle interference.

### Insights into infection strategies shared by *S. sclerotiorum* and *B. cinerea*

#### ROS generation and tolerance

The generation of reactive oxygen species (ROS) has long been associated with defense responses in plant-fungus interactions. The ‘oxidative’ burst is an early and universal plant response following pathogen challenge. Plants resist invading pathogenic fungi, often biotrophs, by induction of the hypersensitive response, a programmed cell death that results from pathogen recognition and is generally associated with the induction of ROS. ROS are detected in the host plant during infection by *S. sclerotiorum* and *B. cinerea*, and likely other necrotrophs. Disease development is impeded when pathogen-generated ROS is inhibited [78]. Whereas ROS-mediated plant cell death is detrimental for biotrophic fungal pathogens, generation of ROS seems advantageous for a necrotrophic pathogen. However, this raises questions as to how *S. sclerotiorum* and *B. cinerea* thrive in a highly oxidative environment.

We examined the two genomes for genes that are involved in ROS generation and tolerance to oxidative stress. Remarkably, both fungi have gene repertoires for ROS-generating enzymes and antioxidants that are very similar to those in other plant pathogenic fungi or in saprophytic fungi (not shown). Regulation of ROS and redox homeostasis is a versatile means for cells to respond to developmental cues. The importance of the ROS/redox “climate” in fungal growth and differentiation was suggested by Hansberg and Aguirre [79] who proposed that hyperoxidant states are a primary driving force leading to differentiated states in fungi. The importance of antioxidant enzymatic systems for effective cellular homeostasis and appropriate development was recently demonstrated in several fungi. The *S. sclerotiorum* and *B. cinerea* genomes each contain two NADPH oxidase encoding genes (*Ssnox*1, *Ssnox*2, *Bcnox*A, *Bcnox*B). Mutants in either of these genes were unable to develop sclerotia. *S. sclerotiorum* mutants in the *Ssnox1* or *Ssnox2* genes differed in virulence: *Ssnox*1 mutants were weakly pathogenic while *Ssnox2* mutants were fully pathogenic (Veluchamy *et al.*, unpublished). The *B. cinerea* mutants were both impaired in virulence but to different extents: *Bcnox*B is important for penetration, whereas *Bcnox*A is involved in lesion expansion. A double mutant is almost avirulent, as is a *Bcnox*R mutant lacking the regulatory subunit of the Nox complex [80]. Since NADPH oxidases are highly conserved and they are involved in differentiation in various fungi [81], [82], their role in *S. sclerotiorum* or *B. cinerea* could primarily be in the differentiation of infection structures.

How can fungi trigger oxidative processes in a plant and at the same time cope with an aggressive oxidative environment? There is little information as to how oxidants and antioxidants are balanced in time and space to provide the specificity required to regulate diverse cellular processes. ROS regulation is complex as there are numerous (enzymatic and non-enzymatic) participants, superimposed in a cellular context that also includes reactive nitrogen species (e.g. nitric oxide; [83]). For example, deletion of the *B.cinerea bap*1 gene, which encodes a transcription factor that controls enzymes required for oxidative stress responses (catalases, peroxidases, GSA,GRX,TRx) results in high sensitivity to oxidative stress *in vitro*, but there was no impact on virulence [84]. Antioxidant genes controlled by Bap1 were not induced in early stages of infection, during which the host mounts an oxidative burst. Thus *B. cinerea* does not sense oxidative stress. There must be alternative protection systems and we suggest this provides an explanation, at least in part, as to why *S. sclerotiorum* and *B. cinerea* do not possess a larger suite of such genes.

#### Biosynthesis of oxalic acid

*S. sclerotiorum* and *B. cinerea* are renowned for the ability to acidify their environment through the secretion of organic acids. In particular, the production by *S. sclerotiorum* of oxalic acid and its association with white mold symptom development has been studied for over a century [85]–[88]. The physiological roles of oxalic acid in pathogenesis are numerous. Oxalic acid enhances the activity of polygalacturonases to promote cell wall degradation [89], inhibits plant–protection enzymes [90], [91], suppresses the plant oxidative burst [92], deregulates stomatal guard cell closure [93], mediates pH signalling [94], induces apoptosis-like cell death [5] and alters the cellular redox-status in the plant [8]. Each of these activities separately may facilitate infection [1], [95]. Oxaloacetate acetyl hydrolase (OAH) activity is associated with oxalic acid accumulation in *S. sclerotiorum* [96] and *B. cinerea* [97]. Genes encoding OAH activity have been identified in the *S. sclerotiorum* and *B. cinerea* genomes (SS1G_08218.1, BC1G_03473.1, BofuT4_132860.1). Deletion mutants in SS1G_08218.1 and BC1G_03473.1 are deficient in oxalic acid biosynthesis [97, Rollins et al, unpublished]. This observation, however, does not establish whether the primary source of oxaloacetate is the TCA cycle, the glyoxalate cycle or both. The dynamic role of oxalic acid in *S. sclerotiorum* and *B. cinerea* physiology is further exemplified by the identification of genes encoding oxalate decarboxylase, which converts oxalate into formate and carbon dioxide. Such activity was previously reported from *S. sclerotiorum* [98]. The biological functions of oxalic acid rely on a dynamic balance of production and breakdown and not simply on a system of maximal synthesis and accumulation. The acidic environment created by oxalate secretion facilitates the optimal activity of certain sets of cell wall degrading enzymes and peptidases (following two sections).

#### *B. cinerea* and *S. sclerotiorum* genomes are especially suited for pectin decomposition

The ability to degrade complex plant carbohydrates is an important aspect of fungal lifestyle. Plant cell wall carbohydrates form a complex network of different polysaccharides that are subdivided in three categories: cellulose, hemicellulose (including xylan, xyloglucan, glucogalactomannan, galactan, and respective side chains), and pectin (composed of galacturonan, rhamnogalacturonan, and respective side chains). This network is the target of carbohydrate-active enzymes and auxiliary proteins (jointly referred to as CAZymes) needed to access internal plant tissues and to degrade plant cell wall components to simple monomers serving as carbon sources. The genomes of *S. sclerotiorum* and *B. cinerea* contain respectively 346 and 367 genes encoding putative CAZymes (Table S18) including at least 118 and 106 CAZymes unambiguously associated with plant cell wall degradation (Tables S19, S20), although many other CAZymes likely contribute to this activity. The CAZyme content in the genomes of *S. sclerotiorum* and *B. cinerea* was compared to other ascomycetes (Table S18) including plant pathogens (namely *B. graminis*, *P. nodorum*, *P. teres f. teres*, *M. oryzae*, *G. zeae*) and two saprobes (*N. crassa*, *A. niger*) to examine whether the lifestyle of these fungi correlates with their CAZyme content and distribution (Table 2 and Table S18). The CAZyme content in *S. sclerotiorum* and *B. cinerea* genomes is smaller than in the other plant pathogens analysed except for *B. graminis*, but equivalent in size to the saprotroph *A. niger*, renowned for its versatility in degrading plant carbohydrates. The CAZyme content in *S. sclerotiorum* and *B. cinerea* is, however, larger than in *N. crassa*, and in the powdery mildew *B. graminis*, suggesting that the evolution of these species has led to different degrees of reduction in their carbohydrate degrading capabilities. In *B. graminis*, the reduction in genes encoding plant cell wall (PCW) degrading enzymes is extreme (Table S19) [10].

**Table 2:** Comparison of the plant cell wall (PCW) degrading potential from CAZome analysis between *S. sclerotiorum* and *B. cinerea* and other Ascomycetes.

Fungal species	Lifestyle	CAZymes per PCW component					
		C	H	HP	P	Total	%P
*Sclerotinia sclerotiorum*	Necrotrophic pathogen	20	40	13	33	106	31
*Botrytis cinerea* T4	Necrotrophic pathogen	18	41	15	44	118	37
*Blumeria graminis*	Biotrophic pathogen	1	7	1	1	10	10
*Phaeosphaeria nodorum*	Necrotrophic pathogen	47	66	28	32	173	18
*Pyrenophora teres f. teres*	Necrotrophic pathogen	38	55	27	25	145	17
*Gibberella zeae*	Hemi-biotrophic pathogen	24	53	27	47	151	31
*Magnaporthe oryzae*	Hemi-biotrophic pathogen	37	68	32	19	156	12
*Neurospora crassa*	Saprotroph	26	31	13	8	78	10
*Aspergillus niger*	Saprotroph	16	38	20	45	119	38

The values reflect the total number of members in the families strictly associated with each PCW component. Substrates: C - cellulose; H - hemicellulose; HP - hemicellulose and pectin side chains; P -pectin; %P - percentage of PCW-degrading enzymes using pectin as substrate.

Besides the quantitative differences, the CAZymes of *S. sclerotiorum* and *B. cinerea* display qualitative differences to those of the other plant pathogens analysed (Table 2, Table S19). *S. sclerotiorum* and *B. cinerea* have a noticeably lower number of enzymes putatively involved in degradation of hemicellulose (H, Table 2, Table S19). The pectin degrading capacity is larger for *B. cinerea* than for *S. sclerotiorum* (44 vs. 33 putative pectin-specific CAZymes, respectively). Their potential pectin degrading capacity is comparable to that of *P. nodorum* and *M. oryzae*, but significantly smaller than in *Fusarium oxysporum* and *Nectria haematococca* (73 and 74 pectin-specific CAZymes, respectively; data not shown). By contrast, the number of cellulose and hemicellulose degrading enzymes encoded in *S. sclerotiorum* and *B. cinerea* genomes is smaller than in all the other PCW-degrading pathogens. Different PCW-degrading pathogens likely use different approaches to decompose plant tissues. Even if the cellulose degrading capacity is similar between pathogens, their ability to cope with soft tissue components, *e.g.* hemicellulose and pectin, is likely to vary. Many saprotrophs degrade plant cell wall material in a manner totally different from *S. sclerotiorum* and *B. cinerea*. For instance, the CAZyme content in the genomes of *N. crassa* (Table S19), as well as *Hypocrea jecorina* and *Podospora anserina* (not shown) suggests a preference of these fungi for cellulose and hemicellulose rather than for pectin. Interestingly, the pectin-degrading CAZyme content of *S. sclerotiorum* and *B. cinerea* genomes shows similarities with that of the saprotroph *A. niger*. In particular, the cellulolytic, hemicellulolytic and pectin degrading capacities of *B. cinerea* and *A. niger* are strikingly similar. These two unrelated fungi are not only similar in the number of enzymes in all pectin-related CAZY categories, but also in the ratio of pectin-degrading lyases versus hydrolases. Such observations suggest a common preference for soft plant tissue (such as flowers or fruit), which is rich in pectin.There are also differences between *B. cinerea* and *A. niger*, particularly in the number of cellulose-binding modules (CBMs, Table S20). These categories are larger in *S. sclerotiorum* and *B. cinerea*, indicative of a stronger preference for vegetative plant tissues.

Among the unusual features of the *S. sclerotiorum* and *B. cinerea* genomes is the presence of a large set of enzymes likely involved in α-1,3-glucan degradation (family GH71). Many of the GH71 catalytic modules are attached to CBM24 modules, with up to four CBM24 modules linked to one GH71 module in a single polypeptide. Whether *S. sclerotiorum* and *B. cinerea* use such enzymes for the degradation of their own cell walls, the walls of antagonistic fungi, or for plant polysaccharides is presently unknown. Interestingly, a significant number of CBM18 (chitin-binding) modules is also present in both genomes, although the number of CAZymes involved in degrading chitin in the genomes of *S. sclerotiorum* and *B. cinerea* is low. Since several of the CBM18 modules are attached to CE4 chitin deacetylase modules, it is possible that these polypeptides contribute to reducing the release of chitooligosaccharides, which is known to lead to responses by host plants. Similar expansion of the CBM18 family is observed in all pathogenic fungi analysed (Table S20).

*S. sclerotiorum* and *B. cinerea* and five other ascomycetes were grown on monosaccharides and on plant-derived polysaccharides to establish correlations with their CAZyme content (Figure S9). Best growth for both *S. sclerotiorum* and *B. cinerea* was observed on pectin, while both grew poorly on xylan and cellulose. By contrast, fungi with a high number of genes encoding xylan- or cellulose-degrading enzymes grew better on these substrates while those containing a lower number of genes encoding pectin-degrading enzymes (*M. oryzae*, *N. crassa*) grew poorly on pectin. The CAZyme annotation and carbon growth profile suggest a specialization of *S. sclerotiorum* and *B. cinerea* towards degrading the pectin fraction of the plant cell wall. Their poor growth on xylan is due to a combination of a reduced xylanolytic system and the inability to use xylose, the main component of xylan, as carbon source. It is likely that the remaining ability to degrade hemicelluloses is not used for the release of nutrients, but rather for decomposition of plant cell walls to enable infection.

Transcriptome analysis identified 43 *B. cinerea* (11.1%) and 40 *S. sclerotiorum* (11.2%) CAZyme-encoding genes whose expression was significantly upregulated during colonization of sunflower cotyledons as compared to *in vitro* grown mycelium, whereas 6 B. *cinerea* (1.5%) and 10 *S. sclerotiorum* (2.8%) CAZyme-encoding genes were significantly downregulated (Table S21). While no pair of orthologs was downregulated in the two fungi, 17 pairs of orthologs were significantly upregulated *in planta* in both *B. cinerea* and *S. sclerotiorum*. Sunflower cell walls consist largely of cellulose, glucuronoxylans and pectin and to a lesser extent of mannans and xyloglucans [99]. Among the *B. cinerea* genes significantly upregulated *in planta*, 12 encode cellulose degrading enzymes, 3 xylan degrading enzymes, 6 pectin degrading enzymes and 2 mannan degrading enzymes (Table S21). This suggests that rather than focusing on specific components, *B. cinerea* produces a broad range of enzymes that is able to degrade both major and minor components of the sunflower cell wall. A similar pattern was observed for *S. sclerotiorum* during infection with upregulation of genes encoding enzymes involved in the degradation of cellulose (6), xylan (8), pectin (11) and mannan (2).

#### Secreted peptidases

Pathogenic as well as saprotrophic fungi secrete peptidases to degrade a variety of (poly)peptides in their environment. This degradation is potentially beneficial in eliminating the activity of antifungal host proteins and in providing nutrients. The genomes of *S. sclerotiorum* and *B. cinerea* were analysed for genes encoding proteins with predicted proteolytic activity, with emphasis on the secreted peptidases (Table 3). *S. sclerotiorum* and *B. cinerea* have a variety of peptidases comparable to the Eurotiomycetes and Sordariomycetes. The number and variety of peptidases encoded by the *B. graminis* genome is strongly reduced. *S. sclerotiorum* and *B. cinerea* possess more secreted sedolisins (S53, acidic optimum) than *G. zeae* and *M. oryzae*, which in turn have many more subtilisins (S8A, alkaline optimum). Sedolisins and subtilisins belong to the same clan (SB) and share the same ancestral peptidase [100]. *S. sclerotiorum* and *B. cinerea* also have two genes encoding glutamic peptidase (G01), an enzyme that is active at pH 2 [101]. Altogether *S. sclerotiorum* and *B. cinerea* possess a large number of genes encoding peptidases with an acidic pH optimum and a small number of genes encoding peptidases with a basic pH optimum. These data suggest an adaptation of the peptidase gene content in *S. sclerotiorum* and *B. cinerea*, to perform well in a low pH environment generated by the production of oxalic acid (see section: Biosynthesis of oxalic acid). A notable difference between *S. sclerotiorum* and *B. cinerea* is that the *S. sclerotiorum* genome contains only 9 aspartic proteinase (A01A)-encoding genes while *B. cinerea* has 14 such genes [102], however, the functional consequences of this observation are unknown.

**Table 3:** Secreted peptidases in *S. sclerotiorum*, *B. cinerea*, and other ascomycetes.

Clan	Family	Name	pH	Leotiomycetes			Dothideomycetes		Sordariomycetes			Eurotio-mycetes
				Ss	Bc	Bg	Pn	Pt	Gz	Mo	Nc	An
**AA**	**A01A**	**Aspartic Proteinase**	A	8(1)	13(1)*	4	8(3)	7(1)	15(1)	14	13(1)	6(2)
**CA**	**C13**	**Legumain**	A	1**	1	1	1	1	1	1	1	1
**GA**	**G01**	**Glutamic peptidase**	A	2	2(1)	1	1	1	1	1	0(1)	3(1)
**MA**	**M12**	**Astacin/adamalysin**	N	0(1)	1(1)	0	3	3	1	1(1)	0(1)	1(1)
	**M35**	**Deuterolysin**	A-N	1(1)	1(1)	0	2	1	1	4(5)	2	0
**MC**	**M14**	**Carboxypeptidase A**	N-B	1	1(1)	0	4	4	4	6	1	1
**ME**	**M16**	**Pitrilysin**		0(4)	2(2)	0(1)	0(4)	0(3)	3(3)	0(3)	0(3)	0(5)
**MJ**	**M19**	**Membrane dipeptidase**		0(1)	1	0	0(1)	0(1)	0(1)	0(2)	0(1)	0(1)
**MH**	**M20**	**Carboxypeptidase Ss**	A	2(4)	2(4)	0	4	2(3)	5(1)	1(5)	0(3)	2(7)
	**M28**	**Aminopeptidase Y**	N	3(1)	1(2)	0	11	8(3)	9(1)	13(2)	3(1)	4(1)
**PA**	**S01A**	**Chymotrypsin**	N	1	1	0	2	1	1	1	0	0
	**S01B**	**Glutamyl peptidase**		0(1)	0	1	0(1)	0(1)	0(2)	0(1)	0(1)	0
	**S01X**	**S1 unassigned peptidase**		0	0	0	0	0	0	1	0	0
**SB**	**S08A**	**Subtilisin**	B	3	2(1)	2	6(1)	5	14(5)	21	5	2(1)
	**S08B**	**Kexin**	N	1	1	1	1	1	1	1	1	1
	**S53**	**Sedolisin**	A	7	7(1)	1	3(1)	1(2)	3	3	1	7
**SC**	**S10**	**Carboxypeptidase Y**	A-N	8(1)	8(1)	4(1)	5(1)	8(1)	8	5(1)	4	9
	**S28**	**Pro-X Carboxypeptidase**	B	1(1)	2(1)	2	0	3	2	4	2	2(1)
**SE**	**S12**	**Ala-Ala Carboxypeptidase**	N-B	0(4)	1	0	1(6)	0(2)	2(10)	1(3)	2(4)	1(9)
**SF**	**S26**	**Signal peptidase I**		1	1(1)	1(2)	1(2)	1	0(2)	1(1)	0(1)	0(3)
**SJ**	**S16**	**Lon-A peptidase**		0(1)	1(1)	0(2)	1(1)	0(2)	0(1)	1	0(2)	0(2)

The number of peptidases detected within each genome that are predicted to be directed to the secretory pathway. In parentheses the number of non-secreted peptidases. Not shown are the peptidases of which the activity is restricted to autocatalytic activation of a precursor protein, or peptidases with problematic function inference (e.g. S9 and S33 proteases). pH indicates at which pH a certain family is generally most active (A = Acidic optimum; N = Neutral optimum; B = Basic optimum). Species are abbreviated as: Ss: *S. sclerotiorum*; Bc: *B. cinerea*; Bg: *B. graminis*; Pn: *P. nodorum*.; Pt: *P. teres f. teres*; Gz: *G. zeae*; Mo: *M. oryzae*; Nc: *N. crassa*; An: *A. niger*.

*Based on corrected gene models [102].

**Based on corrected gene models (unpublished).

#### Secreted effector proteins

Secreted effector proteins that are transferred into plant host cells have been recently shown to be essential for pathogenesis in many plant pathogenic microorganisms, for example in the biotrophic powdery mildews [10], smuts [103], and rusts [104] and in the hemibiotroph *M. oryzae* [105]. In the necrotrophic fungi *P. nodorum* and *Pyrenophora tritici-repentis*, host-specific proteinaceous toxins have been demonstrated to serve as effectors that enhance virulence [106], [107]. Up to now, little experimental evidence for the existence of similar effector proteins is available for *B. cinerea* and *S. sclerotiorum*. One exception is the recent identification of a xylanase in *B. cinerea* that acts as a virulence factor, promoting necrosis in the host plant independent of its enzymatic activity [108]. From genes encoding proteins with N-terminal leader peptides (based on SignalP prediction), genes for CAZymes and peptidases were eliminated, resulting in 879 and 603 genes encoding secreted proteins, respectively, in the genomes of *B. cinerea* and *S. sclerotiorum*. The larger size of the *B. cinerea* secretome was also evident for the small secreted proteins: 521 in *B. cinerea* and 363 in *S. sclerotiorum* were smaller than 300 amino acids, and 333 in *B. cinerea* and 193 in *S. sclerotiorum* were smaller than 150 amino acids. Experimental support (microarray expression data, ESTs and OrthoMCL analysis) was obtained for approximately half of the gene models encoding small secreted proteins (respectively 49–51% for <150aa, and 63–65% for <300aa). Comparative expression analysis during *in planta* and *in vitro* growth suggested a role in pathogenesis only for CAZymes, as described above (see section: *B. cinerea* and *S. sclerotiorum* genomes are especially suited for pectin decomposition). For the remaining secretome, the number of genes upregulated *in planta* was not significantly higher than the average of all genes (Table S22). On the contrary, both in *S. sclerotiorum* and *B. cinerea*, more genes encoding secreted proteins were downregulated than upregulated *in planta*.

### Differences in infection strategies between *S. sclerotiorum* and *B. cinerea*

#### Comparative infection transcriptomics

At least 69% of predicted genes in *S. sclerotiorum* and *B. cinerea* appears to be expressed, as concluded from their representation in at least one of the cDNA libraries tested (Figure S10) or from hybridization signals in custom oligoarrays. Transcripts were found to be differentially expressed in equal proportions in both fungi, when comparing sunflower-inoculated cotyledons (48 hpi) and mycelium grown *in vitro*: 192 *S. sclerotiorum* (1.8%) and 253 *B. cinerea* (1.75%) predicted genes are upregulated in leaves while 173 *S. sclerotiorum* (1.65%) and 247 *B. cinerea* (1.7%) genes are downregulated. When considering *B. cinerea/S. sclerotiorum* orthologs, it appears that 50% of the genes significantly upregulated during sunflower colonization in one species have a similar expression pattern in the other. This proportion was smaller (20%) for the genes that are downregulated during sunflower colonization. The common set of genes that are upregulated *in planta* suggests that the two species share at least some mechanisms to colonize plant tissues. The lists of upregulated *S. sclerotiorum* and *B. cinerea* genes are enriched in CAZyme-encoding genes relative to the whole gene complement (9-fold for both species), with 40–43 genes in each species (approx. 11% of the CAZy genes in each of the genomes) (see section: see section: *B. cinerea* and *S. sclerotiorum* genomes are especially suited for pectin decomposition). There is also an enrichment in P-type ATPases, MFS-type sugar transporters and peptidase-encoding genes. Of the genes that are only present in one of the two species, it appears that *B. cinerea* exhibits a two-fold higher number of *in planta* upregulated genes than *S. sclerotiorum*. This suggests that *B. cinerea* and *S. sclerotiorum* may have adopted species-specific strategies for infection. In addition to the genes that are differentially expressed between the *in vitro* and *in planta* conditions, the expression analysis provided a list of genes that are most highly expressed during infection, including several peptidases and other secreted proteins. Details of experiments, raw values and lists of differentially expressed genes with associated normalized values are available at http://urgi.versailles.inra.fr/Data/Transcriptome.

#### Secondary metabolism

**Figure 6:**
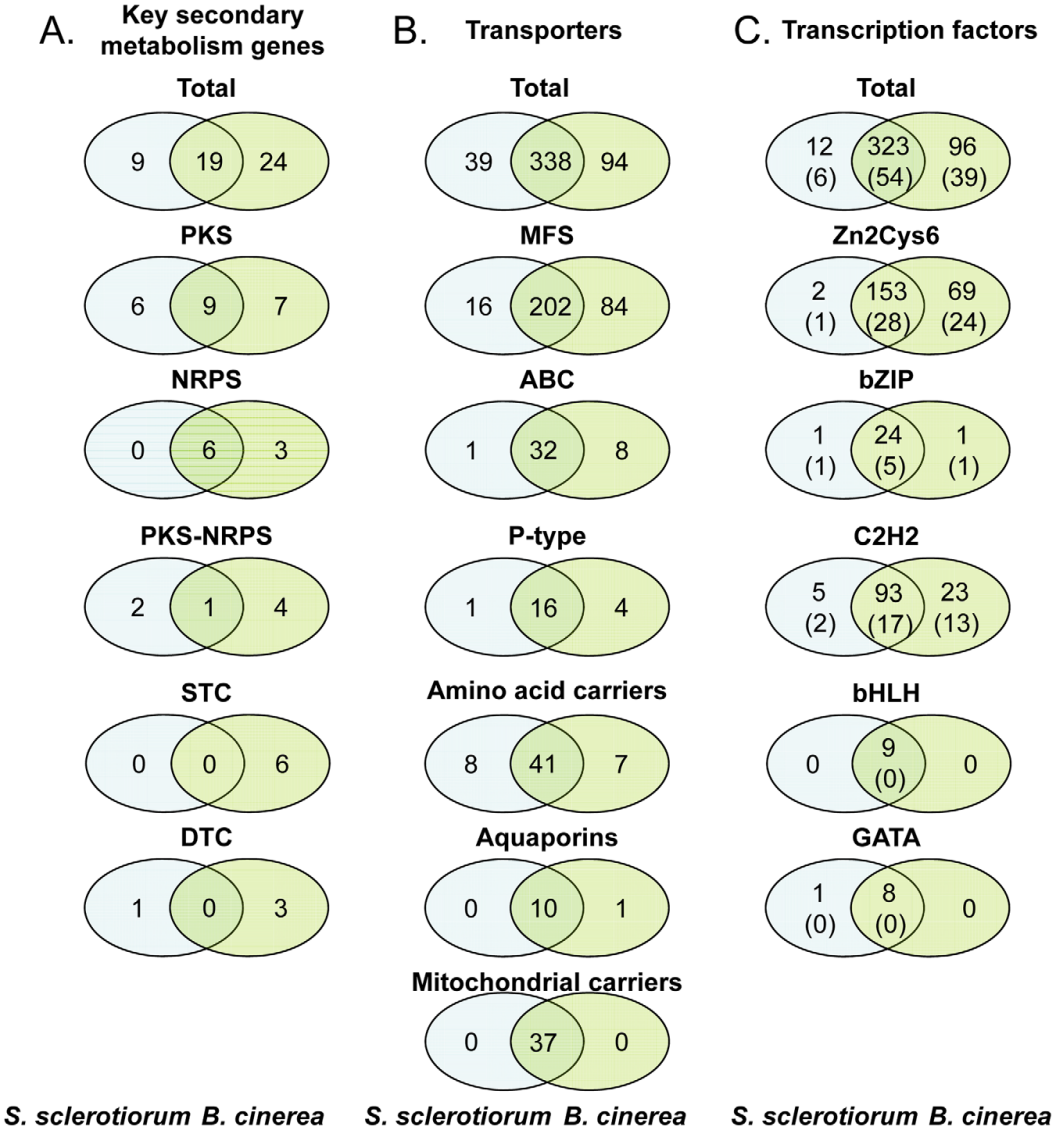
Number of genes encoding secondary metabolism key enzymes, membrane transporters, and transcription factors in the genomes of *S. sclerotiorum* and *B. cinerea*. *S. sclerotiorum/B. cinerea* orthologs were determined by BDBH. Secondary metabolism key enzymes (A), membrane transporters (B), and transcription factors (C). In panel C, the numbers in brackets indicate the number of genes lacking orthologs in other fungi.

One of the crucial weapons that necrotrophic, polyphageous pathogens possess is the production of (non-specific) phytotoxic compounds to kill cells of a range of plant species. Two groups of phytotoxic metabolites produced by *B. cinerea* have been characterized *i.e.* the sesquiterpene botrydial and related compounds [109] and botcinic acid and its derivatives [110]. To identify the pathways involved in the production of other secondary metabolites in *S. sclerotiorum* and *B. cinerea*, we searched the genomes for genes encoding key enzymes such as NRPS (non-ribosomal peptide synthetase), PKS (polyketide synthase), TS (terpene synthase) and DMATS (dimethylallyl tryptophane synthase), which are essential for the biosynthesis of peptides, polyketides, terpenes and alkaloids, respectively [111]. Both genomes contain a significant number of genes encoding key secondary metabolism (SM) enzymes (28 in *S. sclerotiorum*, 43 in *B. cinerea*; main classes in Figure 6A, complete list in Table S23). *S. sclerotiorum* and *B. cinerea* have the potential to produce approximately 26 and 40 main secondary metabolites, respectively, as some SM pathways have more than one key enzyme. The numbers of key enzyme genes are similar to the average found in ascomycetes (Table 4) and significantly higher than in the Leotiomycete *B. graminis*, which has undergone massive gene loss probably due to its exclusively biotrophic life-style [10]. Among the ascomycetes, *B. cinerea* has the highest number of TS from the STC (sesquiterpene cyclase) sub-family, although similar numbers of STC genes were identified in basidiomycetes [112]. Most *S. sclerotiorum* and *B. cinerea* key SM genes (90%) belong to clusters of genes that encode other biosynthesis enzymes, regulators and/or transporters. Generally in fungi, all genes from a SM cluster are involved in the same metabolic pathway and are co-regulated [111], [113], [114]. In *S. sclerotiorum* and *B. cinerea*, about one third of the clusters contain a gene encoding a Zn(II)2Cys6 transcription factor, that could control the expression of genes within its own cluster. Also, 40% of the SM clusters contain a gene encoding an ABC or MFS transporter that could export the metabolites produced by the enzymes encoded by the gene cluster. SM clusters are randomly distributed in the genome of *S. sclerotiorum* (Figure S11), and there was no enrichment in the location of clusters in sub-telomeric regions. By contrast, SM gene clusters in *Aspergillus spp.* and *G. zeae* are frequently (50%) located in sub-telomeric regions [67], [115].

**Table 4:** Secondary metabolism key enzyme-encoding genes in fungal genomes.

Fungal species	PKS^1^	NRPS^2^	PKS–NRPS^3^	CHS^4^	DMATS^5^	STC^6^	DTC^7^
*Sclerotinia sclerotiorum*	15	6	3	1	1	0	1
*Botrytis cinerea*	16	9	5	1	1	6	3
*Blumeria graminis*	1	1	0	1	0	0	0
*Phaeosphaeria nodorum*	19	8	1	2	0	2	1
*Pyrenophora teres f. teres*	18	27	2	1	1	1	1
*Gibberella zeae*	11	19	2	1	0	3	0
*Magnaporthe oryzae*	22	8	10	2	3	1	2
*Neurospora crassa*	5	3	1	1	1	0	1
*Aspergillus niger*	15	12	5	1	0	2	2

Data from this study and from [10]–[12], [157], [158].

1 PKS: PolyKetide Synthase.

2 NRPS: Non-Ribosomal Peptide Synthetase.

3 PKS-NRPS: PolyKetide Synthase - Non-Ribosomal Peptide Synthase hybrid.

4 CHS: CHalcone Synthase.

5 DMATS: DiMethyl Allyl Tryptophan Synthase.

6 STC: SesquiTerpene Cyclase.

7 DTC: DiTerpene Cyclase.

*B. cinerea* has a higher number of genes encoding key SM enzymes (43) compared to *S. sclerotiorum* (28, Figure 6A). This difference is even more striking when considering orthologs and paralogs (Figure 6A, Table S23). Only 19 key SM genes correspond to orthologous pairs in both genomes, while 24 genes are only found in *B. cinerea* and 9 only in *S. sclerotiorum*. The 19 shared key SM genes belong to 17 clusters that have a comparable organization between the two genomes (microsynteny), with one exception. *B. cinerea* genes BcPKS6 and BcPKS9 are jointly responsible for production of the phytotoxin botcinic acid. In *B. cinerea* these PKS genes are in different genomic locations while their orthologs in *S. sclerotiorum* are part of the same cluster. This suggests that a major genomic rearrangement, either a fusion or fission, has occurred in one of the species [116]. Among the other clusters shared by the two species (detailed in Text S3) are those that are conserved among ascomycetes and are involved in biosynthesis of melanin (PKS13), coprogen (NRPS6) and intracellular siderophores (NRPS2, NRPS3).

For nearly all key SM genes present in both *S. sclerotiorum* and *B. cinerea*, orthologs were found in other fungal genomes. The *S. sclerotiorum* or *B. cinerea* key SM genes found in only one of the two genomes, were called “specific”, although most have orthologs in at least one other fungal genome. The 9 *S. sclerotiorum* “specific” key SM genes encode PKS (6 genes) and PKS-NRPS hybrids (2 genes). *S. sclerotiorum* also has one specific TS corresponding to a non-classical DTC (diterpene cyclase) with an additional GGPP synthase domain (Table S23). This bifunctional terpene synthase is orthologous to the fusicoccin synthase (FUS) from *Phomopsis amygdali* [117] although the cluster is only partially conserved with *P. amygdali* suggesting that *S. sclerotiorum* might produce a metabolite different from fusicoccin. The 24 *B. cinerea* “specific” key SM genes encode TS (10 genes), PKS (7 genes), NRPS (3 genes) and PKS-NRPS hybrids (4 genes). Remarkably, *S. sclerotiorum* does not contain STC genes, while *B. cinerea* has 6, including the STC1 (also named BcBOT2) gene which is part of the cluster required for synthesis of the phytotoxin botrydial [118]. *B. cinerea* also contains a TS encoding gene (PAX1) orthologous to the *Penicillium paxilli PaxC* gene, involved in biosynthesis of the indole-diterpene paxillin [119] (Table S23).

The genomic regions located upstream and downstream of the *B. cinerea*-specific SM clusters were detected in the *S. sclerotiorum* genome. Genes from the surrounding regions have the same physical organization in both species, demonstrating that these regions were syntenic, except for the presence or absence of a given SM cluster. As discussed above, loss of synteny between both genomes is frequently associated with the occurrence of TE-rich regions in the *S. sclerotiorum* genome. In several cases, the loss of synteny at a locus with a species-specific SM cluster indeed appears to be associated with the presence of TEs in one of the genomes. For example, the *B. cinerea*-specific DTC3 gene cluster corresponds to a locus in the *S. sclerotiorum* genome where a reverse transcriptase is detected, as well as a relic of the DTC3 ortholog. This observation suggests that a deletion in *S. sclerotiorum* has removed most of the original DTC3 cluster. The striking difference in the content of SM clusters between *S. sclerotiorum* and *B. cinerea* might reflect a recent gain of clusters in *B. cinerea*, or the loss of clusters in *S. sclerotiorum*. As most of the SM key enzyme genes found in *B. cinerea* have orthologs in other fungi, the most probable hypothesis is that the SM clusters were present in the common ancestor of the Sclerotiniaceae and that many of them were lost in *S. sclerotiorum* as exemplified for the DTC3 gene cluster.

Overall, 42 and 65% of *B. cinerea* and *S. sclerotiorum* SM clusters, respectively, are orthologous. Most of the shared clusters are conserved among ascomycetes and involved in elementary cellular processes including cell protection (melanin) and iron scavenging (siderophores). On the other hand, the capacity for producing SMs considerably differs between these two related species, despite their similar behaviour as plant pathogens. More than half of the *B. cinerea* SMs cannot be produced by *S. sclerotiorum*. Two of the *B. cinerea*-specific SM clusters are involved in production of the phytotoxin botrydial [118] and the phytohormone abscisic acid [120]. In addition, *B. cinerea*-specific SM genes are highly expressed during sunflower cotyledon colonization (see 6.1) which suggests they may be involved in the production of metabolites important for infection.

#### Transporters

Plant pathogens must protect themselves, especially during infection where they are likely to encounter host defense mechanisms. Membrane transporters play a role in counteracting the physiological impact of antimicrobial host defense compounds. For example, the *B. cinerea* ABC transporter AtrB has been shown to be required for tolerance to the *Arabidopsis* phytoalexin camalexin [121]. Mutations leading to overexpression of AtrB and a MFS-type drug efflux transporter are responsible for the appearance of *B. cinerea* field strains with increased tolerance to unrelated fungicides (multidrug resistance) in French and German vineyards [122]. The genomes of *S. sclerotiorum* and *B. cinerea* were analysed for genes encoding nutrient transporters, mitochondrial carriers, aquaporins and efflux pumps. ABC-transporters utilizing ATP hydrolysis to provide energy for transport [123] have a conserved architecture of one nucleotide binding fold (NBF) and six transmembrane-spanning helices (TMD) in different arrangements (Table 5). The major facilitator superfamily (MFS) proteins use proton-motive force for substrate translocation. The differential regulation of 17 MFS-type hexose transporter genes during colonization of sunflower cotyledons provided support for their involvement in sugar uptake during pathogenesis [124]. Analyses of fungal genomes for the content of membrane transporter-encoding genes did not reveal major differences between saprotrophs and pathogens [125], [126]. Comparison of the numbers of transporter genes in the genome of *S. sclerotiorum* with the two *B. cinerea* strains revealed a bias towards larger sizes of certain families in *B. cinerea* (Table 5; Figure 6B). While 42 and 40 ABC transporter genes were identified in *B. cinerea* B05.10 and T4, respectively, only 33 were identified in *S. sclerotiorum*. Similarly, *B. cinerea* contains 30% more MFS transporter genes (282–286) as compared to *S. sclerotiorum* (218). Differences in gene numbers were less evident or not observed for P-type ATPases, amino acid transporters, mitochondrial carriers and aquaporins (Figure 6B). The majority of *B. cinerea* genes that were absent in *S. sclerotiorum* did not have orthologs in other fungi, indicating that gene expansion in *B. cinerea* rather than gene loss in *S. sclerotiorum* has likely been the driving force for the observed differences. Approximately half of the transporter genes were highly expressed both in mycelium and during infection (Table S24). Members of the MFS transporter gene families showed higher expression levels during growth *in planta* as compared to growth *in vitro*. By contrast, the majority of MDR-related MFS transporters were repressed during infection. Several P-type ATPases were upregulated *in planta* in *B. cinerea* but not in *S. sclerotiorum*.

**Table 5:** Frequency of occurrence of predicted genes encoding membrane transporters.

Category	*S. sclerotiorum*	*B. cinerea* T4	*B. cinerea* B05.10
**ABC transporters**	**33**	**40**	**42**
[(TMD_6_)-NBF)]_2_	18	21	23
[(NBF-(TMD_6_)])_2_	8	12	12
NBF-(TMD_6_)	3	3	3
(TMD_6–10_)-NBF	4	4	4
**MATE transporters**	**2**	**2**	**2**
**P-type ATPases**	**17**	**20**	**20**
**MFS transporters**	**218**	**286**	**282**
sugar transporters	51	68	68
MDR-related transporters	85	109	107
other MFS transporters	78	107	105
**Amino acid transporters**	**49**	**48**	**48**
**Mitochondrial carriers**	**37**	**37**	**37**
**Aquaporins**	**10**	**11**	**11**

ATP binding cassette (ABC) transporters are subdivided into different structural types, according to the arrangement of transmembrane domains (TMD) and nucleotide binding folds (NBF). Not included are proteins containing NBF but no TMD. MATE: Multidrug and Toxic Compound Extrusion. MFS: Major facilitator superfamily. MDR: Multidrug resistance.

#### Transcription factors

Transcription factors (TFs) are essential players in regulatory networks. Thirty-seven TF families have been identified in fungi (http://supfam.org/SUPERFAMILY) among which six are specific to the fungal kingdom [127]. Despite the close phylogenetic relatedness between *S. sclerotiorum* and *B. cinerea*, there are striking differences in the number of TFs between both species. 330 TF-encoding genes were identified in *S. sclerotiorum*, and 392–410 in *B. cinerea* (Table S25). The difference between the two fungi also extends to the distribution among families, i.e. a large number of TFs are from the Zn(II)2Cys6 binuclear cluster family (*S. sclerotiorum* 155 and *B. cinerea* 222, Figure 6C) and the Zn(II)-coordinating C2H2 (*S. sclerotiorum* 98 and *B. cinerea* 116). The total number of TFs shared (i.e. encoded by orthologous genes) between *S. sclerotiorum* and *B. cinerea* is 323. Among them, 54 (17%) have no ortholog in other sequenced fungal genomes (Figure 6C). Additionally, *B. cinerea* has 96 TFs that are absent in *S. sclerotiorum*, while only 12 *S. sclerotiorum* TFs are absent in *B. cinerea*. These three categories of TFs mostly (86%) belong to Zn(II)2Cys6 binuclear cluster and C2H2 zinc finger families. Indeed, *S. sclerotiorum* and *B. cinerea* share 153 Zn(II)2Cys6 binuclear cluster TFs (Figure 6C), while an additional set of 69 of these TFs are found only in *B. cinerea*. For the C2H2 zinc finger family, *S. sclerotiorum* and *B. cinerea* share 93 TFs, while an additional set of 23 C2H2 TFs are found only in *B. cinerea*. Among the 69 *B. cinerea*-specificZn(II)2Cys6 binuclear cluster TFs lacking in *S. sclerotiorum*, 9 are located in secondary metabolism gene clusters that are not present in *S. sclerotiorum*.

Overall, among the *B. cinerea* TFs that have no counterpart in *S. sclerotiorum*, 57 out of 96 (60%) are encoded by genes that have orthologs in other fungal genomes, while 39 are *B. cinerea-*specific. This distribution suggests that 57 TFs conserved among fungi have been lost in *S. sclerotiorum*. The cellular processes controlled by these TFs are possibly altered in *S. sclerotiorum*. It would be of interest to characterise the regulatory networks that, in other fungi, are controlled by these conserved TFs. Such studies might provide clues about the cellular deficiencies occurring in *S. sclerotiorum* (e.g. the inability of asexual sporulation). Additionally, the function of the 39 *B. cinerea*–specific TFs remains to be investigated.

### Conclusions

Comparative genomics of *S. sclerotiorum* and *B. cinerea* has revealed an expected high degree of sequence identity and synteny, however, we observed several striking differences in gene content between these plant pathogens. The first difference was in the content of transposable elements. The data suggest a recent burst of transposition in the *S. sclerotiorum* genome relative to *B. cinerea*. The two species differ in the regulation of sexual reproduction and this is fully explained from the sequence and organization of the MAT loci. Features of these loci provide evidence for an evolution of heterothallism from homothallism. The failure of *S. sclerotiorum* to produce conidiospores could not be explained from the gene content, as all key conidiation genes seemed to be present and potentially functional. The genome sequences further revealed a striking difference in the amount and types of potential secondary metabolites. The botrydial toxin biosynthetic cluster and the phytohormone abscisic acid biosynthetic genes were unique to *B. cinerea*. The precise chemical nature and biological function of most of these metabolites remains to be determined to understand whether they contribute to the adaptation of the two species to different ecological niches. The content of CAZyme-encoding genes revealed a preference of *S. sclerotiorum* and *B. cinerea* for pectin as nutrient source. This is in agreement with the observation that *S. sclerotiorum* and *B. cinerea* are pathogens of dicots and preferentially grow on aerial plant tissues that are rich in pectin. With respect to pathogenicity determinants, there are no unique features that could be identified as ‘silver bullets’, which distinguish these aggressive pathogens from other pathogenic and non-pathogenic fungi. Comparison with two other necrotrophic fungi revealed only few (if any) genes shared among necrotrophs and absent in fungi with other trophic lifestyles (i.e saprotrophs or hemi-biotrophs). These findings point to the multigenic, variable, and sophisticated nature of necrotrophic pathogenicity whose nuance may only be revealed through systematic functional analysis of candidate activities and regulators. We suggest that the specific regulation of networks of the available suite of genes is key to pathogenic success for *S. sclerotiorum* and *B. cinerea*.

## Materials and Methods

### Materials and methods for phylogenetic analysis

#### Isolate sampling

The isolates included in the sample were 9 species in the genus *Botryotinia* (including the genome strains B05.10 and T4), 4 species in the genus *Sclerotinia* (including the sequenced strain 1980), 4 members of the Sclerotiniaceae (*Monilinia fructicola*, *Myriosclerotinia scirpicola*, *Sclerotium cepivorum* and *Dumontinia tuberosa*), 3 members of the Rutstroemiaceae (*Lambertella langei*, *Lambertella subrenispora* and *Sclerotinia homoeocarpa*) with the powdery mildew *Blumeria graminis* as the presumed outgroup. Details of the isolates are provided in Table S26.

#### Fungal cultures and DNA extraction

All isolates were grown on potato dextrose agar (Difco Laboratories, Detroit, MI) for 3–5 days, and were then transferred to standing culture potato dextrose broth (Difco Laboratories) for 1–2 days. On both solid and liquid media, cultures were grown in the dark at ambient room temperature (20–22°C). Cultures were filtered through Miracloth (Calbiochem, EMD Chemicals Inc., Darmstadt, Germany) and lyophilized. DNA was extracted from 10–15 mg of lyophilized mycelium using a DNeasy Plant Mini Kit (Qiagen, Mississauga, ON). Quantity of DNA was estimated on 1.0% ethidium bromide-stained agarose gels, with known quantities of bacteriophage lambda DNA as standards against which comparisons of band intensity could be made. DNA extractions were diluted to 10–20 ng/µL in elution buffer (10 mM Tris-Cl, 0.5 mM EDTA; pH 9.0) for use in PCR.

#### PCR and sequencing

An approximately 500 bp segment of the internal transcribed spacer (ITS) region, and portions of genes encoding actin (ACT), calmodulin (CAL), glyceraldehyde-3-phosphate dehydrogenase (G3PDH), and heat shock protein 60 (HSP60) were amplified using the primer sets listed in Table S27. Polymerase chain reactions were performed using GoTaq Colourless Master mix (Promega Corporation, Madison, WI) containing: GoTaqDNA Polymerase in 1× Colorless GoTaqReaction Buffer (pH 8.5), 200 µM dNTPs, and 1.5 mM MgCl_2_; 0.2 µM of each primer; 10–20 ng of DNA template in the total volume of 50 µL per reaction. PCR was performed under the following conditions in a GeneAmp PCR System 9700 programmable thermal cycler (Applied Biosystems, Foster City, CA): denaturing at 95°C for 2 min followed by 35 cycles of 30 s denaturing at 95°C, 30 s annealing at corresponding temperatures for each primer set (Table S27), and 1 min elongation at 72°C, followed by a final extension step at 72°C for 5 min. PCR fragments were visualized by electrophoresis on a 1.0% ethidium-bromide stained agarose gel, using a 100 bp ladder (BioShop Canada Inc., Burlington, ON) to estimate the size of the fragments. All PCR products were purified and sequenced using 3730×l DNA Analyzer systems (Applied Biosystems, Foster City, CA) at the McGill University and Genome Quebec Innovation Centre.

#### Phylogenetic analyses

Sequences were aligned in Clustal X v. 1.81 [128] and further edited manually. Maximum parsimony (MP) and maximum likelihood (ML) analyses were performed in PAUP* 4.0b10 [129] with heuristic searches. Characters were treated as unordered and gaps as missing data. Bootstrap support for internal branches was estimated from 1000 pseudoreplicates for both MP and ML analyses. Models of sequence evolution were estimated for each locus with ModelTest 3.7 [130], [131], and the phylogenetic parameters are shown in Table S28.

Bayesian analyses were performed for each genetic locus in Mr. Bayes v.3.0b4 [132] to estimate the posterior probabilities of tree topology with Metropolis-coupled Markov chain Monte Carlo (MCMCMC) searches. All analyses employed one cold chain and three incrementally heated chains, where the heat of the *i*th chain is b = 1/[1+(*I*−1)T] and t = 0.2; when *I* = 1, B = 1, corresponding to the cold chain. Independent analyses of each of the four loci were conducted with 2,000,000 generations each, with a sampling frequency of 1 tree every 100 generations. The average standard deviation of split frequencies stabilized (to a difference of less than one percent) after 10,000 generations in all our analyses. Therefore the initial 10,000 generations from each run were discarded as burn-in when summarizing tree parameters and topology. Flat Dirichlet probability densities were used as priors for the substitution rate parameters and stationary nucleotide frequencies and uniform priors were used for the shape and topology parameters and an exponential unconstrained prior was used for the branchlengths parameter.

Analyses of the combined (concatenated) dataset were performed using ML and Bayesian inference. A partitioned dataset with each partition corresponding to each genetic locus with a unique evolutionary model for each partition (Table S28) was used for the Bayesian analysis. The heuristic ML approach employed a combined data set with the following single, overall model of evolution re-estimated from ModelTest 3.7: a general time reversible model with gamma distribution with invariant sites (base frequencies = [0.2580, 0.2512, 0.2277, 0.2631]; rate matrix = [1.3214, 4.0315, 1.3851, 0.8728, 7.8478]; shape parameter for gamma distribution = 0.9427; proportion of invariant sites = 0.3866).

The resultant tree topology is shown in Figure 2, with bolded branches indicating well-supported nodes (ML bootstrap values >90% and Bayesian posterior probabilities >0.95; Table S29). The concatenated tree was rooted with members of the Rutstroemiaceae, previously shown to be an outgroup to the Sclerotiniaceae [13], [14], [132].

### Sequencing, assembly, and physical mapping

The sequenced strain of *S. sclerotiorum* ‘1980’ is available from the Fungal Genetics Stock Center (http://www.fgsc.net/). Plasmid (4-kb and 10-kb inserts) and Fosmid (40-kb inserts) libraries were generated with randomly sheared and size-selected DNA. Plasmid and Fosmid inserts were sequenced from both ends to generate paired reads. Sequence reads (total of 507,621) were filtered for quality, vector and other contamination, and the resulting 476,001 reads were assembled with the Arachne assembler [20]. A consensus sequence was determined from an average of approximately 9.1-fold sequence depth (7.8 depth in Q20 bases). The assembly (GenBank accession AAGT01000000) totals 38.3 Mb and consists of 36 scaffolds with an N50 of 1.6 Mb. The scaffolds consist of 679 sequence contigs with a N50 of 123 kb; at least half of all bases in the assembly fall within at least the size of the N50 scaffold or contig. The contigs total 38.0 Mb, so only 0.8% of the assembly is represented by contig gaps. This assembly contains 98.7% of bases with Q40 quality or greater.

The sequence of *B. cinerea* strain B05.10 was generated by Syngenta AG using four size selected shotgun libraries. Sequence reads were filtered for quality, vector and other contamination, and the resulting 291,603 reads were assembled using the Arachne assembler. A consensus sequence was generated from an average of approximately 4.5 fold sequence depth (3.9-fold depth in Q20 bases). The assembly (GenBank accession AAID00000000) totals 42.3 Mb and consists of 4,534 contigs with a N50 of 16.4 kb, which are ordered and oriented by paired end clone reads within 588 scaffolds, with a N50 of 257 kb.

The sequence of *B. cinerea* strain T4 (Genbank accessions FQ790245 to FQ790362) was determined and assembled by Genoscope, Evry, France. The genome was sequenced using a whole genome shotgun (WGS) strategy, by generating plasmid (3 kb and 10 kb inserts) and BAC (∼50 kb inserts) libraries. Clones from these libraries were end-sequenced to generate paired reads. Sequence reads from plasmid inserts (573,705, representing 10.5× coverage) were assembled with Arachne [20], resulting in 3,054 contigs. Contigs were ordered and oriented into scaffolds using paired sequences from BACs (35,668 reads). The gap lengths were estimated using linked clones. 118 scaffolds larger than 20 kb with gap sizes lower than 10%, were used for annotation. These 118 scaffolds (39.5 Mb, N50 of 562 kb, Table 1) contain 2,281 contigs (37.9 Mb, N50 of 35 kb). This assembly contains 98.7% of bases with Q40 quality or greater.

An optical map, a type of physical map, was created for *S. sclerotiorum* strain 1980 by OpGen. The restriction enzyme *Bsi*WI was used to create ordered fragments and the resulting map contained 16 optical contigs. These likely correspond to complete chromosomes, in agreement with an estimated chromosome number of 16 using pulsed-field gel electrophoresis [21]. The assembly was compared to the optical map using an *in silico* digest of the assembly (http://www.broadinstitute.org/annotation/genome/sclerotinia_sclerotiorum/MultiHome.html). This comparison suggested that supercontig 4 contained an unsupported join in the assembly, between contigs 132 and 133; the assembly was updated to break scaffold 4 between these contigs, and a revised version of the assembly was submitted to Genbank. No other major disagreements were found. A total map size of approximately 38.5 Mb was estimated by calculating the ratio between the assembly fragments and map units. The assembly supercontigs cover 99.4% of the optical map (contigs cover 98.6%) (Figure S1). Only two supercontigs, 35 and 36, were not anchored to the optical map; these supercontigs are 39 kb and 16 kb respectively, and do not contain any *Bsi*WI restriction site. Supercontig 35 consists of arrayed ribosomal DNA repeats. The optical contigs appear to correspond to complete chromosomes, as they end with a flush set of DNA molecules (data not shown). Additionally, reads containing telomeric repeat arrays of (TTAGGG) are linked to the ends of 26 of the 32 chromosome ends, and are present at the end of two additional scaffolds (Figure S1). At least two scaffolds are mapped to each optical contig, suggesting that centromeres could lie in these unassembled gaps.

### Genetic linkage map of *B. cinerea*

68 individual progeny from a cross between strains T4 (isolated from tomato) and 32 (isolated from *Vitis vinifera*) were kindly provided by Caroline Kunz (Université Pierre et Marie Curie, Paris). Around 400 microsatellite markers were designed from the genomic sequences of the B05.10 and T4 strains by using the “GRAMENE” software [133]. In addition, a set of 144 SNP (Single Nucleotide Polymorphism) markers were identified through the comparison of the B05.10 and T4 genome sequences and genotyped in the progeny using the SNPlex genotyping assay (Applied Biosystems). The segregation of markers among the progeny was analysed using MAPMAKER software [134] set at min LOD3 and max Distance at 37.

### Gene prediction

For *S. sclerotiorum* and *B. cinerea* B05.10, gene structures were predicted using a combination of FGENESH and GENEID. The version of GENEID used for these calls was 1.2a. FGENESH is unversioned. As FGENESH uses a statistical model of gene structure that requires training on each organism for accurate prediction, Softberry trained FGENESH on *S. sclerotiorum* sequences. GENEID is an *ab initio* gene caller and was run with the default parameters after being trained on a set of 542 *S. sclerotiorum* genes that were manually curated based on EST and protein alignments.These *S. sclerotiorum* trained gene models were also used for *B. cinerea* B05.10 without additional training.

The results from these two gene callers were combined in the following manner. Both FGENESH and GENEID were run on the entire genomic sequences of *S. sclerotiorum* and *B. cinerea* (B05.10) to provide an initial set of predicted genes. This resulted in a set containing 12,961 FGENESH predictions and 14,711 GENEID predictions for *S. sclerotiorum*, and 13,864 FGENESH predictions and 16,907 GENEID predictions for *B. cinerea*. Next, gene predictions less than 30aa (90 nt) were removed, and any gene prediction less than 50aa (150 nt) was removed, unless it was overlapped by a prediction from a different program, BLAST evidence, a HMMER PFAM domain, or an EST alignment. Applying these criteria removed 2,166 *S. sclerotiorum* and 2,373 *B. cinerea* genes from consideration. We manually annotated 1,141 *S. sclerotiorum* and 751 *B. cinerea* genes. FGENESH and GENEID predictions were clustered based on overlapping exons, requiring strand consistency. For each locus, a gene structure was chosen based on BLAST similarity to other proteins (requiring ≥60% average identity and ≥80% query coverage), and selecting the gene prediction from the program with best overall agreement to EST splice sites (GENEID performed better than FGENESH by this metric). The initial gene sets contained 14,522 genes for *S. sclerotiorum* and 16,448 genes for *B. cinerea*.

The accuracy of the gene set was evaluated by comparing to EST sequences. For *S. sclerotiorum*, a set of 75,468 ESTs that were sequenced as part of this project or available in Genbank, were aligned to the genome using BLAT. EST alignments were clustered by combining all overlapping ESTs. Each of the resulting 7,400 EST clusters was compared to any overlapping predicted genes. In cases where multiple overlapping ESTs suggest different gene structures, the EST that most closely matched the gene structure was used. Roughly one-third of genes (5,192) have some overlap with an EST cluster; of these, 75% show no splice site disagreements. A small number of predicted genes appear partial in *S. sclerotiorum*: 4 are missing a start codon, 6 are missing a stop codon, and 98 span contigs. Possible missed annotations include ESTs with no overlapping gene; a total of 559 clusters are at least 200 bases from an annotated gene, and of these 88 contain canonical splice signals. After the automated annotation was complete, ESTs from additional libraries were generated and aligned to the genome. In total, we sequenced ESTs from 8 libraries, generating 96,700 quality filtered sequences (Table S30). These ESTs align to 7,942 genes.

For *B. cinerea* (B05.10), a set of 9,207 ESTs were aligned to the genome using BLAT. EST alignments were clustered by combining all overlapping ESTs into a cluster. Each of the resulting 2,349 EST clusters was compared to overlapping predicted genes. In cases where multiple overlapping ESTs suggest different gene structures, the EST that most closely matched the gene structure was used. Roughly one-eighth of genes (2,012) have some overlap with an EST cluster; of these, 82% show no splice site disagreements. A small number of predicted genes appear partial in *B. cinerea* (B05.10): 67 are missing a start codon, 153 are missing a stop codon, and 444 span contigs. Possible missed annotations include ESTs with no overlapping gene; a total of 132 clusters are at least 200 bases from an annotated gene, and of these only 4 contain canonical splice signals.

*S. sclerotiorum* and *B. cinerea* (B05.10) gene calls were further evaluated to remove transposable elements and other dubious calls. Genes with at least 100 bases of overlap with repeats called by RepeatMasker or BLAST similarity against a transposon-related protein database, or containing a PFAM repeat domain but not a PFAM non-repeat domain, were flagged as dubious gene calls. Also, genes that have 5 or more BLAST hits at ≥95% identity to the same genome and have no supporting ESTs, BLAST hits or non-repeat PFAM domains, were flagged as dubious. To identify poorly supported gene calls, two additional gene predictors, GeneMark and SNAP, were run on both genomes. Genes less than 100 amino acids that were only supported by a single gene predictor (comparing GENEID, FGENESH, GeneMark, and SNAP) and without EST, BLAST, or PFAM domain support were flagged as dubious genes. Additionally, genes with two predictions that do not share the same reading frame were flagged. In total, all these methods flagged 2,662 *S. sclerotiorum* genes and 2,784 *B. cinerea* (B05.10) genes as dubious. All predicted genes were used to query the PFAM set of hidden Markov models using HMMER (http://hmmer.janelia.org) and the public protein databases using BLASTP. Transfer RNAs were identified using the tRNAScan-SE program.

For *B. cinerea* T4, the automated gene prediction was performed using the URGI genomic annotation platform including pipelines, databases and interfaces, developed for fungi (http://urgi.versailles.inra.fr/). Gene prediction was performed using Eugene pipeline version 3 [135]. The gene models predicted by EuGene rely on a combination of several *in silico* evidences (*ab initio* and similarity). *Ab initio* gene prediction softwares are Eugene_IMM [136] (probabilistic models discriminating coding from non coding sequences), SpliceMachine [137] (prediction of CDS start sites and intron splicing sites) and FGENESH (http://linux1.softberry.com/berry.phtml) (*ab initio* gene predictor). Several similarity methods were used to identify genes such as BLASTN and Sim4 against *B. cinerea* ESTs, as well as BLASTX against Uniprot and fungal protein databases. The different results were used by Eugene to predict final gene models. The three *ab initio* gene prediction softwares were trained using a set of manually annotated genes from *B. cinerea*. FGENESH was trained by Softberry (Boston, USA), while EuGene-IMM and SpliceMachine were trained at URGI using a set of 305 genomic/full-coding cDNA pairs. One third of the set was used for training *ab initio* softwares, one third to optimize the parameters of EuGene and the last third to calculate the accuracy of EuGene. We finally obtained for exons and genes, a sensitivity of 97.8 and 92.1 and a specificity of 97.5 and 92.1, respectively. Genes with a size smaller than 100 nucleotides were automatically filtered out by EuGene. EuGene finally predicted 16360 genes in the *B. cinerea* T4 genome. In addition, 434 tRNA genes were predicted by tRNAscan-SE [138].

### Genome alignment and synteny

Genome assemblies were aligned using MUMmer [139]. The *B. cinerea* genomes were aligned with nucmer, selecting only 1∶1 matches (−mum), and alignments were processed with delta-filter to select 1∶1 local mapping of the reference to query. A total of 35.5 Mb of each could be aligned, or 94% of the *B. cinerea* T4 genome and 91% of the *B. cinerea* B05.10 genome. A total of 98,744 insertion/deletion positions and 175,009 SNPs were identified from the nucmer alignments using the show-snps program. This suggests that the overall rate of difference between the two genomes in aligned regions is 1 SNP every 203 bases and 1 insertion/deletion every 360 bases. *S. sclerotiorum* and *B. cinerea* are too distant to align well at the nucleotide level globally; only a total of 7.4 Mb can be aligned at 85.0% identity. Therefore the *B. cinerea* genomes were aligned to *S. sclerotiorum* using promer, selecting only 1∶1 matches (−mum), and alignments were processed with delta-filter to select 1∶1 local mapping of the reference to query. The promer alignments cover 17.4 Mb sharing 74.3% identity for *S. sclerotiorum*-*B. cinerea* T4 and 17.2 Mb sharing 74.6% identity for *S. sclerotiorum*-*B. cinerea* B05.10.

To identify larger syntenic regions between genomes, we first identified orthologs using BLASTP as well as OrthoMCL. Using BLASTP (1e-5, softmasked), *S. sclerotiorum* shares 8,609 best bi-directional (bbd) BLAST hits with *B. cinerea* B05.10 or 8,601 with *B. cinerea* T4; 8,088 *S. sclerotiorum* proteins have matches in both genomes. About 1,300 additional proteins in *S. sclerotiorum* have BLAST matches that are not best bidirectional. Using OrthoMCL to cluster the three genomes, which uses BLASTP similarity for clustering, there are a total of 12,095 protein families, of which 8,079 are in all three genomes and nearly all of these (7,677) are single copy. Either raw BLAST data or ortholog sets were then used to find syntenic regions of at least four genes between genomes using DAGchainer [27], and parsed using accessory perl scripts. Syntenic regions between the two *B. cinerea* genomes include 11,422 paired genes and cover 36.9 Mb in 249 regions (average size 148 kb). Syntenic regions between *S. sclerotiorum* and *B. cinerea* T4 include 7,752 paired genes and cover 27.7 Mb in 571 regions (average size 49 kb). Syntenic regions between *S. sclerotiorum* and *B. cinerea* B05.10 include 7,702 paired genes and cover 27.9 Mb in 602 regions (average size 46 kb). Segmental duplication blocks were identified by comparing each genome against itself, using DAGchainer [27].

### Transposable elements detection and annotation

Transposable Elements (TEs) were detected and annotated using the REPET pipeline [30], [31] http://urgi.versailles.inra.fr/Tools/REPET) for detection (TEdenovo) and annotation (TEannot) of transposons in eukaryotic genomes. The TEdenovo pipeline detects TE copies, groups them into families and defines the consensus sequence for each family containing at least 3 copies. The TEannot pipeline annotates TEs using the consensus sequences library. Manual annotation of the consensus sequences allowed their grouping in TE super-families corresponding to a transposon with a defined full-length copy (with both LTRs or ITRs and full ORFs when available) and classified as classI or classII TE. In addition, the genome of *B. cinerea* T4 was searched using TBLASTN for sequences similar to either the transposase of *B. cinerea* mariner element Flipper [140] or the reverse transcriptase from Boty1 [141]. Sequences with similarities to TE-encoded proteins were grouped and aligned to identify or reconstruct the corresponding full-length TE. Manually identified TEs were compared to the consensus sequences obtained from REPET.

### Functional annotation of predicted genes

Automated functional annotation of *S. sclerotiorum* and *B. cinerea* proteins was performed using protein sequences deduced from all gene models automatically predicted. The pipeline uses protein domain identifier InterProScan [142] which runs a set of methods including pattern matching and motif recognition. In addition, we used an automated assignment against protein domains databases such as CDD [143], KEG [144] and KOG [145]. Sub-cellular targeting signals as well as transmembrane domains were predicted using SignalP, TargetP and TMHMM [146]. Orthologs in *S. sclerotiorum* were determined based on best bi-directional BLAST hits. Overall, 72% of *B. cinerea* T4 predicted genes (11767 of 16360) encode proteins having either functional information and/or an ortholog in *S. sclerotiorum*. This percentage increases to 82% taking into account only reliable genes.

### Analysis of support for the predicted genes from the three genomes

Four types of evidence were used to support gene calls, based on bioinformatics or experimental criteria (Figure S3). The first criterium was based on identification of functional domains (e.g. InterProScan, CDD) or topology/targeting domains (e.g. SignalP, TargetP, TMHMM) (see M7). Genes with at least one positive hit (functional or topology/targeting domain) were considered as supported by protein domain evidence. The second criterium was based on identification of orthology to genes in other fungi using OrthoMCL (see M9). The third criterium relied on expression data using ESTs (existence of at least one EST for a gene) (see M10). The fourth criterium relied on expression data using hybridization signals on a whole-genome oligonucleotide Nimblegen microarray (see M11). Among the 16,360 predicted genes in *B. cinerea* T4, 13,555 (83%) genes are supported by at least 1 type of evidence and 6,462 (39.5%) genes are supported by all 4 types of evidence. Among the 16,448 predicted genes in *B. cinerea* B05.10, 13,922 (85%) genes are supported by at least 1 type of evidence and 6,319 (38.5%) genes are supported by all 4 types of evidence. Among the 14,522 predicted genes in *S. sclerotiorum*, 12,283 (85%) genes are supported by at least 1 type of evidence and 4,121 (28%) genes are supported by all 4 types of evidence.

### Conservation of *S. sclerotiorum* and *B. cinerea* predicted genes relative to other ascomycetes

Orthologs between *S. sclerotiorum*, *B. cinerea*, and other ascomycete fungi were identified using OrthoMCL version 1.4 [147]. The Ascomycete genomes included in the analysis were *B. graminis* (22_02_11; blugen.org), *P. nodorum* (20110506 from Richard Oliver; Broad Institute), *P. teres f. teres* (PRJNA50389; Genbank), *G. zeae* (FG3; Broad Institute), *M. oryzae* (MG8; Broad Institute), *N. crassa* (NC10; Broad Institute), and *A. niger* (AspGD). Each set of proteins was blasted against itself and other proteomes with an e value of 1e-5. An inflation parameter of 1.5 was used for Markov Clustering with MCL. 12,120 *B. cinerea* (T4) proteins, 12,260 *B. cinerea* (B05.10) proteins, and 9,930 *S. sclerotiorum* proteins were identified as members of gene families; of these about 1,500 were conserved only in the two species (Table S8). OrthoMCL families enriched in *S. sclerotiorum* and *B. cinerea* were identified based on a hypergeometric distribution (p-value computed by phyper function in R; q-value computed by p.adjust in R), however significantly enriched families (q-value<0.05) included only repetitive elements or families specific to only these species. To identify functions specific to these lineage specific proteins, we mapped GO terms to the protein sets of *S. sclerotiorum* and *B. cinerea* (B05.10) using Blast2GO, and computed enrichment using Fisher's Test exact test.

To identify functions enriched in *S. sclerotinia* and *B. cinerea* proteins, we identified PFAM domains in each of the above genomes, and computed enrichment or depletion in subsets of species. Protein domains from PFAM release 25 (ftp://ftp.sanger.ac.uk/pub/databases/Pfam) were assigned to proteins in each genome using hmmsearch from hmmer3 (http://hmmer.janelia.org/software), requiring an Evalue cutoff of 1e-5. For each genome, the total number of proteins with each type of domain was computed; a protein with multiple domains of the same type was counted only once. To identify domains enriched in subsets of genomes, significant differences were identified by computing the p-value for each domain based on a hypergeometric distribution (phyper function in R), and computing q-values to correct for multiple testing (p.adjust, fdr). Domains significantly enriched in the *S. sclerotiorum* and *B. cinerea* genomes (Table S10) were filtered to remove those found in transposable elements (PF03221, Tc5 transposase DNA binding domain; PF00078, Reverse transcriptase; PF00665, Integrase core domain; PF03184, DDE superfamily endonuclease; PF03732, Retrotransposon gag protein; PF00075, RNase H; PF05225, helix-turn-helix, Psq domain). Domains significantly depleted in the *S. sclerotiorum* and *B. cinerea* genomes (Table S11) were filtered to list those that are conserved in at least 5 species.

### EST libraries

For *S. sclerotiorum*, a total of 96,700 filtered EST sequenced were generated from eight cDNA libraries, prepared from mRNA from developing sclerotia, developing apothecia following 55 h light exposure, mycelia at pH 7, infected *Brassica* or infected tomato, two infection cushion samples, and mycelium exposed to oxidative stress (Table S30). ESTs were aligned to the *S. sclerotiorum* genome using BLAST and compared to predicted gene structures.

Fourteen cDNA libraries of various *B. cinerea* isolates (T4, B05.10, SAS56×SAS405, ATCC 58025) and stages of development have been prepared and sequenced in various laboratories (Table S31). Some of them were publicly available [148], [149] (library BcA1 and AL11), others were sequenced in the framework of the *B. cinerea* sequencing project by Genoscope (http://www.genoscope.cns.fr/, libraries PD0A*) while others are private (Bayer Crop Sciences/P. Tudzynski). From these 14 cDNA libraries (Table S31), 78,755 bacterial clones were obtained and sequenced once or twice (5′ end; 3′ end), leading to 83,117 ESTs (1 or 2 ESTs per clone).

Eighty six percent of ESTs, (71,238 ESTs, 67,625 clones, Figure S10) were successfully clustered on the *B. cinerea* T4 genome and assembled using Phrap [http://www.phrap.org/] to finally obtain 9,667 EST contigs. As some genes were lying in different contigs, the 9,667 contigs were assembled in 9,004 unisequences. In order to get an estimate of the expression of genes corresponding to the unisequences, we calculated the raw_clone_nr sum (clone number in all libraries), the raw_clone_nr (clone number in the current library), the percent_clone (100* raw_clone_nr/clone_nr in the current library), and the norm_percent (percent_clone normalized by the percent_clones in all libraries = 100 * (percent_clone)/sum(percent_clone) in all libraries). Eighteen percent of unisequences without any corresponding genes automatically predicted (mostly due to gap in the genome sequence) were used to design oligos for the Nimblegen microarrays.

### Microarray experiments

A Nimblegen 1-plex array was designed using 21,200 *B. cinerea* gene models corresponding to 19,454 ORFs either identified in T4 or B05.10 genomes (12,071 T4 ORFs shared between T4 and B05.10; 93 B05.10 ORFs shared between T4 and B05.10; 4,072 T4 ORFs specific to T4; 3,218 B05.10 ORFs specific to B05.10), 12 experimental genes and 1,734 additional EST unisequences. Nine probes per sequence were defined, leading to 169,347 probes representing 20,889 genes and covering 20,916 provided gene models (19,222 ORFs, 12 experimental genes and 1,682 ESTs). In addition, 16,871 random probes (negative controls) were designed. Two copies of each probe were placed on the array.

A second Nimblegen 1-plex array was designed using 15,026 *S. sclerotiorum* gene models corresponding to 14,522 ORFs and 504 additional EST unisequences. Thirteen probes per sequence were defined, leading to 190,130 probes representing 14,801 genes and covering 14,858 provided gene models (14,360 ORFs and 498 ESTs). In addition, 9,047 random probes (negative controls) were designed. Two copies of each probe were placed on the array.

Expression of fungal genes was studied during infection on sunflower cotyledon, and compared with *in vitro* expression. The experimental conditions were: (i) Infection of sunflower cotyledons by mycelial plugs of *B. cinerea* (B05.10) and *S. sclerotiorum* at 2 days after inoculation (100% of the surface area was infected), (ii) mycelial cultures grown *in vitro* (malt agar) for each fungal strain, and (iii) non-infected sunflower cotyledons. RNAs were extracted from 3 biological replicates, labelled and hybridized to arrays. 2 or 3 biological replicates per experimental condition were exploitable. Data were analysed using ANAIS methods [150]. Probe hybridization signals were normalized using the quantile function, and summed for each gene. Genes were considered as expressed when the signal was above the defined background (1.5 fold the 95^th^ percentile of random probes hybridization signals), in all the biological replicates of at least one experimental condition. The identification of differentially expressed genes was performed using an ANOVA test. To deal with multiple testings, the ANOVA p-values were further submitted to Bonferroni correction. Transcripts with a corrected p-value<0.05 and more than 2.0 fold change in transcript level were considered as significantly differentially expressed. Details of experiments, raw values and lists of differentially expressed genes with associated normalized values are available at http://urgi.versailles.inra.fr/Data/Transcriptome.

### Database resources and interface

#### *B. cinerea* T4 resources (http://urgi.versailles.inra.fr/Species/Botrytis)

Databases and Genome Browser (Gbrowse, Apollo) system relies on the international open source project Generic Model Organism Database (GMOD: http://www.gmod.org). The database was populated with *B. cinerea* T4 genome sequences (supercontigs and contigs) and features predicted or mapped to these sequences: predicted genes, proteins from Uniprot and local fungal protein database, ESTs from 14 *B. cinerea* cDNA libraries (83,000 ESTs, 9667 unisequences), ESTs from *S. sclerotiorum* (70,000), genes from *B. cinerea* strain B05.10 and *S. sclerotiorum*, genome supercontigs of *B. cinerea* (B05.10) and *S. sclerotiorum*, Repeats (Transposable Elements and Tandem Repeats). Results from automated gene prediction are currently manually curated using an editing interface (Apollo), leading to an increase in quality and reliable annotation (http://urgi.versailles.inra.fr/Tools/Apollo). We have also developed and setup an integrated system relying on the Genome Report System (unpublished, http://urgi.versailles.inra.fr/grs/index.html) set up to display functional annotation data on proteins for both *S. sclerotiorum* and *B. cinerea* (T4, B05.10). Searches are processed through GnpIS QuickSearch (http://urgi.versailles.inra.fr/gnpis).

#### *B. cinerea* B05.10 and *S. sclerotiorum* resources

Sequence data from the Broad Insitute is available from http://www.broadinstitute.org/annotation/genome/sclerotinia_sclerotiorum/MultiHome.html and http://www.broadinstitute.org/annotation/genome/botrytis_cinerea/.

### Peptidases

Complete proteome sequences were downloaded from NCBI or JGI and subjected to Merops Batch BLAST analysis [151]. Sequences from *S. sclerotiorum* and *B. cinerea* identified by Merops as putative peptidases were scrutinised based on the presence of active site and ligand residues as well as e values (cut off 1e-5). Additional BLAST analysis was performed at NCBI in order to confirm or reject suspicious hits. We excluded from the analysis the peptidases of which the activity is restricted to an autocatalytic activation of the precursor protein. We also excluded putative peptidases with problematic function inference such as the S9 and S33 proteases. The peptidases of *S. sclerotiorum* and *B. cinerea* were subjected to SignalP analysis [152]. Analyses of the other fungal peptidases were restricted to the families of which at least one sequence was predicted to correspond to a secreted peptidase and performed as described.

### Methods for CAZy annotation

The best protein models of the *S. sclerotiorum* and *B. cinerea* genomes were subject to expert analysis using the CAZy database (www.cazy.org) annotation pipeline [153]. Each model was compared by BLAST [154] to libraries of known catalytic and carbohydrate-binding modules derived from the CAZy database and from previously analyzed genomes. Each identified protein model was subject to modular analysis compring BLAST [154] and HMMer [155] analysis against CAZy-derived libraries and HMM profiles, respectively, followed by human curation. Later, the quality of each identified model was manually evaluated and an expert functional annotation was proposed by comparison against characterized enzymes from CAZy. The approaches are described in more detail elsewhere [153]. Finally, comparative analysis was performed against other fungal genomes using the same principles as described before [156].

### Genbank accession numbers

Assemblies and annotations were submitted to GenBank under the following accession numbers: AAGT01000000 (*S. sclerotiorum*), AAID00000000 (*B. cinerea B05.10*), FQ790245 to FQ790362 (*B. cinerea T4*).

## Supporting Information

Figure S1 Alignment of *S. sclerotiorum* scaffolds to optical map and location of telomeres. *S. sclerotiorum* assembly scaffolds (blue and black numbered rectangles, alternating colors) were aligned to the optical map contigs (grey boxes) based on shared restriction sites (see Materials and Methods). Scaffold 4 is split into 2 pieces (4a and 4b) at a contig gap based on this alignment (see Materials and Methods). The two smallest scaffolds (35 and 36) could not be placed on the optical map due to lack of anchoring restriction sites. Telomeric repeat arrays of TTAGGG were detected specifically in sequence at or linked to scaffold ends, as shown with green circles.
(PDF)

Figure S2 GC content distribution of *S. sclerotiorum*, *B. cinerea*, and other representative Pezizomycotina fungi. Percent GC for 10 kb windows is plotted on the x-axis, and percent of all genome windows for each GC bin is plotted on the y-axis. *S. sclerotiorum* (Ss), *B. cinerea* (Bc) and *B. graminis* (Bg) show a shifted lower %GC profile compared to the rest of these genomes. *N. crassa* (Nc) is unique in displaying a bimodal distribution of %GC. Other genomes shown are *Aspergillus fumigatus* (Af), *Aspergillus nidulans* (An), *Magnaporthe oryzae* (Mo), *Gibberella zeae* (Gz), and *Phaeosphaeria nodorum* (Pn).
(PDF)

Figure S3 Evidence for accuracy of gene prediction in *S. sclerotiorum* (A), *B. cinerea* T4 (B) and *B. cinerea* B05.10 (C). Four types of evidence were combined: (i) Functional annotation (at least one domain/motif), (ii) Orthology between *S. sclerotiorum*, *B. cinerea* and other fungi, (iii) EST support, (iv) Nimblegen Microarrays support.
(PDF)

Figure S4 Gene length distribution before and after filtering of dubious gene predictions. Histogram of gene length for *B. cinerea* (Bc) B05.10 and T4 initial and filtered (high confidence) genes, *S. sclerotiorum* (Ss) initial and filtered (high confidence) genes, *N. crassa* (Nc) genes, and *G. zeae* (Gz) genes.
(PDF)

Figure S5 Dotplot view of syntenic regions between genomes.
(PDF)

Figure S6 Diversity of ScTIR1 genomic copies. Full-length genomic copies of ScTIR1 were retrieved from the genome using REPET and aligned (clustalW). The aligment was used to construct a phylogenetic tree using Maximum parsimony. Identical sequences copies are circled.
(PDF)

Figure S7 Gene count per OrthoMCL family for each species. Genes were clustered into families using OrthoMCL for *S. sclerotiorum*, both strains of *B. cinerea*, and 12 other fungal genomes. The x-axis bins measure the number of genes per family for each species, where orphan genes not in families are in the 0 bin, genes in single copy in a family are in the 1 bin, and paralogous genes of 2 or more in a family are in the higher bins. The y-axis varies between the subplots, and counts the total number of genes in each bin per species.
(PDF)

Figure S8 Phylogeny of apoptosis-associated BIR1-homologs in fungi and yeasts. Dark gray boxes indicate homologs with two BIR-domains in a single protein, light gray boxes indicate homologs with one BIR-domain in a single protein, white boxes indicate homologs lacking a BIR-domain.
(PDF)

Figure S9 Growth of *S. sclerotiorum* and *B. cinerea* and five other Ascomycetes on monosaccharides and simple or complex plant polysaccharides. More extensive growth profiles for these and other fungi can be found at www.fung-growth.org.
(PDF)

Figure S10 (A). Representation of *S. sclerotiorum* EST libraries (percentage) corresponding to the 63,810 clones. (B). Representation of each of the *B. cinerea* libraries (percentage) corresponding to the 67,625 clones (71,238 ESTs) mapped and clustered on the *B. cinerea* T4 genome before assembling in EST contigs.
(PDF)

Figure S11 Localization on the *S. sclerotiorum* genome map of genes encoding secondary metabolism key enzymes. Regions that show synteny with *B. cinerea* are indicated in red. SB1 to SB19 indicate genes that are shared between the two species whereas S1 to S9 indicate *S. sclerotiorum*-specific genes (see Table S23 for more details). SB1: PHS1,SB2: PKS1, SB3: PKS2, SB4: PKS8, SB5: PKS9, SB6: PKS10, SB7: PKS12, SB8:PKS13, SB9: PKS18, SB10: PKS21, SB11: CHS1, SB12: PKS6, SB13: NRPS1, SB14:NRPS2, SB15: NRPS3, SB16: NRPS4, SB17: NRPS5, SB18: NRPS6, SB19:DMATS1, S1: SsFUS1, S2: SsPKS3, S3: SsPKS4, S4: SsPKS5, S5:SsPKS7, S6: SsPKS11, S7: SsPKS14, S8: SsPKS15, S9: SsPKS16.
(PDF)

Table S1 Linkage groups in *B. cinerea* based on the analysis of 68 progeny from a cross.
(PDF)

Table S2 Genome summary of Pezizomycotina fungi.
(PDF)

Table S3 Duplicated blocks within the genomes of *B. cinerea* strain B05.10 and *S. sclerotiorum*.
(PDF)

Table S4 Repeats identified *de novo* by cross-match alignments in *S. sclerotiorum* and *B. cinerea* genomes.
(PDF)

Table S5 Repeats identified in *S. sclerotiorum* and *B. cinerea* genomes by RepeatMasker.
(PDF)

Table S6 Number of TE families and TE copies identified in *S. sclerotiorum* and *B. cinerea* genomes using REPET.
(PDF)

Table S7 Number of solo-LTRs and full-length copies of Gypsy/Copia-like retroelements identified in the genomes of *S. sclerotiorum* (Sclery) and *B. cinerea* (Boty).
(PDF)

Table S8 Conservation of *S. sclerotiorum* and *B. cinerea* genes in OrthoMCL families.
(PDF)

Table S9 GO Term enrichment for genes specific to *S. sclerotiorum* and *B. cinerea*.
(PDF)

Table S10 PFAM domain enrichment in *S. sclerotiorum* and *B. cinerea*.
(PDF)

Table S11 PFAM domain depletion in *S. sclerotiorum* and *B. cinerea*.
(PDF)

Table S12 Signaling pathway components in *S. sclerotiorum* and *B. cinerea* as compared to orthologs in three filamentous fungi and *S. cerevisiae*.
(PDF)

Table S13 *S. sclerotiorum* and *B. cinerea* homologs of apoptosis-associated genes found in other fungi.
(PDF)

Table S14 *S. sclerotiorum* and *B. cinerea* orthologs of *Aspergillus nidulans* genes involved in mating and fruiting body development.
(PDF)

Table S15 Orthologous genes shared between *S. sclerotiorum*, *B. cinerea* and sclerotia-producing Aspergilli, *A.flavus* and *A. oryzae*.
(PDF)

Table S16 *S. sclerotiorum* and *B. cinerea* orthologs of conidiation-associated genes in *Aspergillus nidulans* and *Neurospora crassa*.
(PDF)

Table S17 *S. sclerotiorum* and *B. cinerea* orthologs of appressorium-associated genes in *Magnaporthe oryzae*.
(PDF)

Table S18 Total Carbohydrate-active enzyme and associated (CAZy) modules of *S. sclerotiorum* and *B. cinerea* compared to seven other Ascomycetes.
(PDF)

Table S19 Comparison of plant cell wall (PCW) and fungal cell wall (FCW) degrading and modifying CAZyme family subsets between *S. sclerotiorum* and *B. cinerea* and seven other ascomycetes.
(PDF)

Table S20 Comparison of plant cell wall (PCW) and fungal cell wall (FCW) sets of CBM families between *S. sclerotiorum* and *B. cinerea* and seven ascomycetes.
(PDF)

Table S21 Polysaccharide degradation-related genes that are induced upon infection.
(PDF)

Table S22 Expression analysis of genes encoding secreted proteins.
(PDF)

Table S23 Genes encoding secondary metabolism key enzymes in *S. sclerotiorum* and *B. cinerea* genomes.
(PDF)

Table S24 Expression changes of genes encoding membrane transporters during mycelial and *in planta growth of S. sclerotiorum* and *B. cinerea*.
(PDF)

Table S25 Transcription Factor-encoding genes in *S. sclerotiorum* and *B. cinerea*, as compared to other ascomycetes.
(PDF)

Table S26 Fungal strains used for the construction of the phylogeny shown in Figure 2.
(PDF)

Table S27 Primer sets for amplification of loci in the Sclerotiniaceae.
(PDF)

Table S28 Amplification methods and parameters used for phylogenetic analyses of housekeeping loci.
(PDF)

Table S29 Support values for each node in the phylogeny (Figure 2).
(PDF)

Table S30 *S. sclerotiorum* EST sequences generated by this project.
(PDF)

Table S31 *B. cinerea* EST sequences generated by this project.
(PDF)

Text S1 *Botrytis cinerea* genetic map.
(PDF)

Text S2 Dual mating in *Botrytis cinerea* (teleomorph *Botryotinia fuckeliana*).
(PDF)

Text S3 Secondary metabolism gene clusters.
(PDF)
