# Supplementary material for: Genomic Analysis of the Necrotrophic Fungal Pathogens Sclerotinia sclerotiorum and Botrytis cinerea
Source: PLoS Genet. 2011 Aug 18;7(8):e1002230. doi: 10.1371/journal.pgen.1002230 (PMC3158057; doi:10.1371/journal.pgen.1002230)

Figure S1

Alignment of *S. sclerotiorum* scaffolds to optical map and location of telomeres.

S. sclerotiorum assembly scaffolds (blue and black numbered rectangles, alternating colors) were aligned to the optical map contigs (grey boxes) based on shared restriction sites (see Methods). Scaffold 4 is split into 2 pieces (4a and 4b) at a contig gap based on this alignment (see Methods). The two smallest scaffolds (35 and 36) could not be placed on the optical map due to lack of anchoring restriction sites. Telomeric repeat arrays of TTAGGG were detected specifically in sequence at or linked to scaffold ends, as shown with green circles.

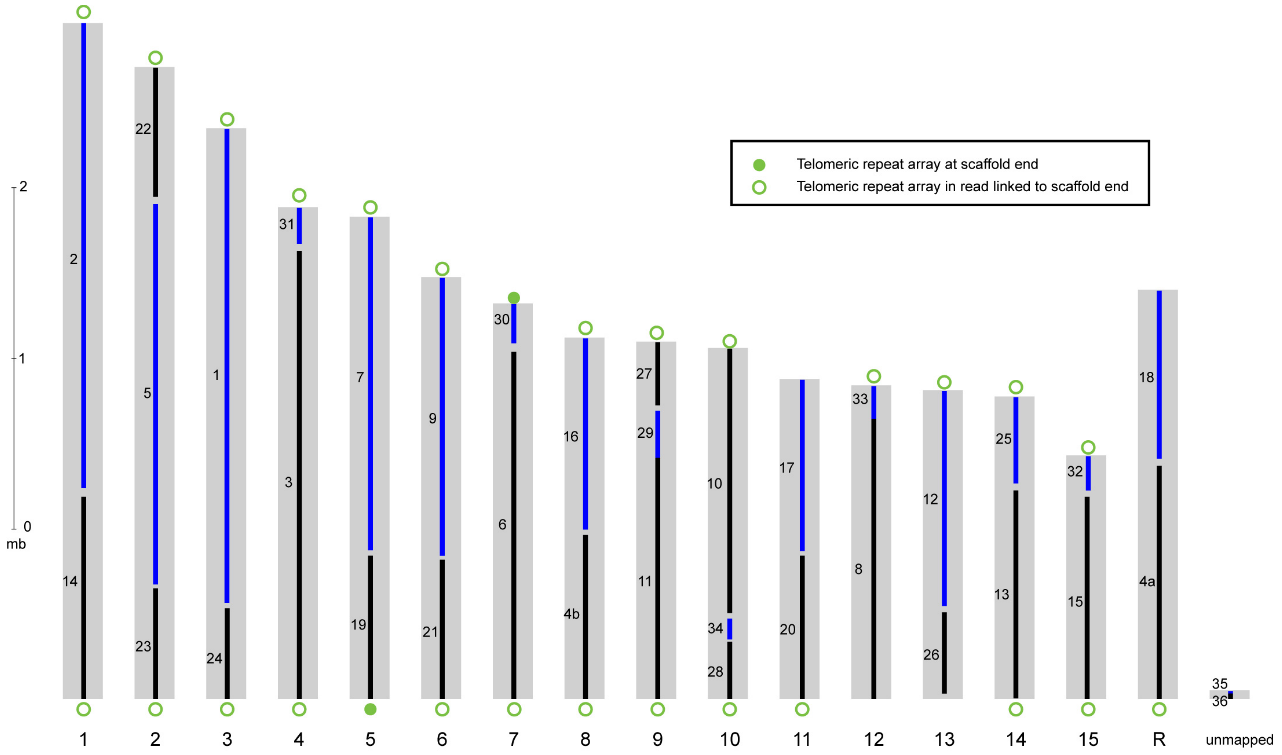

Supplement: Figure S1 — Alignment of S. sclerotiorum scaffolds to optical map and location of telomeres. S. sclerotiorum assembly scaffolds (blue and black numbered rectangles, alternating colors) were aligned to the optical map contigs (grey boxes) based on shared restriction sites (see Materials and Methods). Scaffold 4 is split into 2 pieces (4a and 4b) at a contig gap based on this alignment (see Materials and Methods). The two smallest scaffolds (35 and 36) could not be placed on the optical map due to lack of anchoring restriction sites. Telomeric repeat arrays of TTAGGG were detected specifically in sequence at or linked to scaffold ends, as shown with green circles. (PDF) [file pgen.1002230.s001.pdf]
