# Supplementary material for: Genomic Analysis of the Necrotrophic Fungal Pathogens Sclerotinia sclerotiorum and Botrytis cinerea
Source: PLoS Genet. 2011 Aug 18;7(8):e1002230. doi: 10.1371/journal.pgen.1002230 (PMC3158057; doi:10.1371/journal.pgen.1002230)

Figure S2

GC content distribution of *S. sclerotiorum*, *B. cinerea*, and other representative Pezizomycotina fungi.

Percent GC for 10kb windows is plotted on the x-axis, and percent of all genome windows for each GC bin is plotted on the y-axis. *S. sclerotiorum* (Ss), *B. cinerea* (Bc) and *Blumeria graminis* (Bg) show a shifted lower %GC profile compared to the rest of these genomes. Other genomes shown are *Aspergillus niger* (An), *Pyrenophora teres f. teres* (Pter), *Magnaporthe oryzae* (Mo), *Neurospora crassa* (Nc), *Gibberella zeae* (Gz), and *Phaeosphaeria nodorum* (Pn). *N. crassa* (Nc) is unique in displaying a bimodal distribution of %GC.

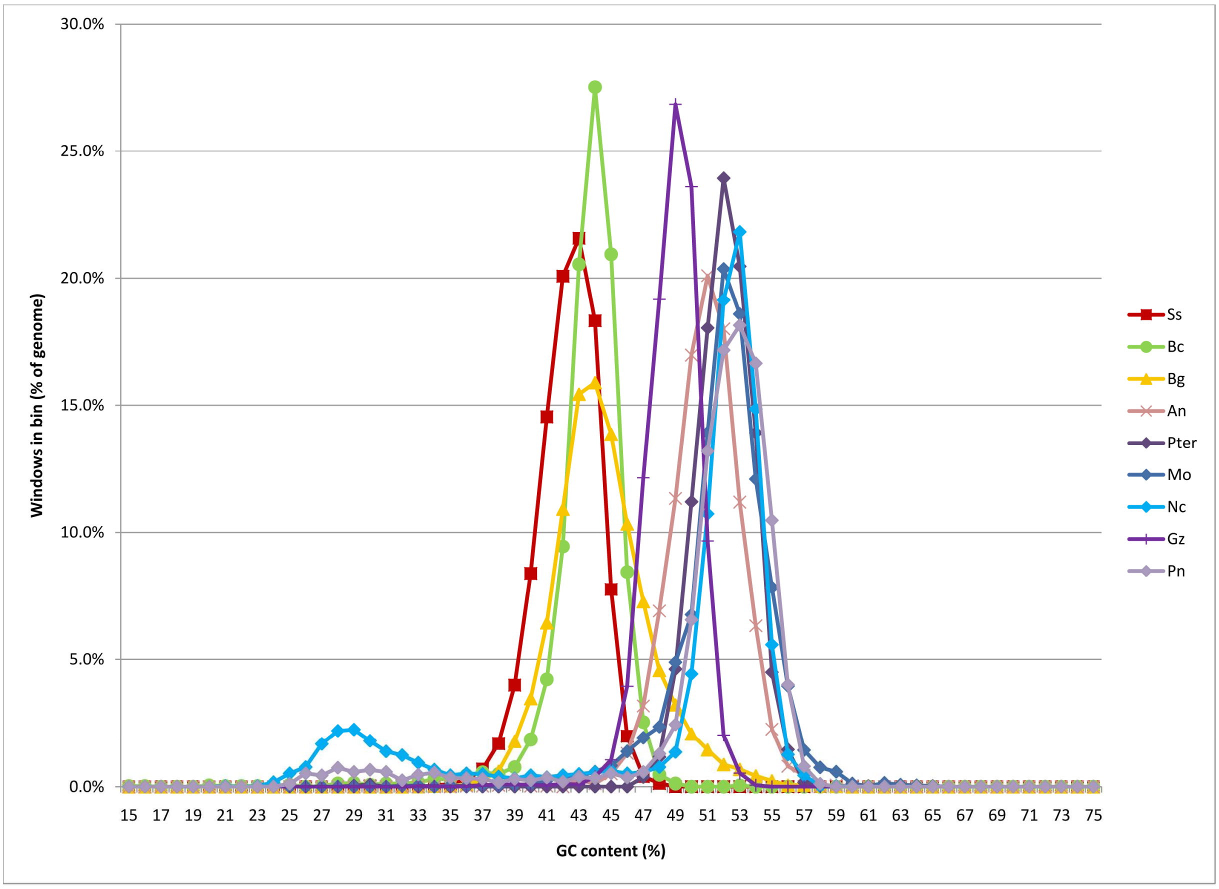

Supplement: Figure S2 — GC content distribution of S. sclerotiorum, B. cinerea, and other representative Pezizomycotina fungi. Percent GC for 10 kb windows is plotted on the x-axis, and percent of all genome windows for each GC bin is plotted on the y-axis. S. sclerotiorum (Ss), B. cinerea (Bc) and B. graminis (Bg) show a shifted lower %GC profile compared to the rest of these genomes. N. crassa (Nc) is unique in displaying a bimodal distribution of %GC. Other genomes shown are Aspergillus fumigatus (Af), Aspergillus nidulans (An), Magnaporthe oryzae (Mo), Gibberella zeae (Gz), and Phaeosphaeria nodorum (Pn). (PDF) [file pgen.1002230.s002.pdf]
