# Supplementary material for: Genomic Analysis of the Necrotrophic Fungal Pathogens Sclerotinia sclerotiorum and Botrytis cinerea
Source: PLoS Genet. 2011 Aug 18;7(8):e1002230. doi: 10.1371/journal.pgen.1002230 (PMC3158057; doi:10.1371/journal.pgen.1002230)

Figure S3

Evidence for accuracy of gene prediction in *S. sclerotiorum* (A), *B. cinerea* T4 (B) and *B. cinerea* B05.10 (C).

Four types of evidence were combined: (i) Functional annotation (at least one domain/motif), (ii) Orthology between *S. sclerotiorum, B. cinerea* and other fungi, (iii) EST support, (iv) Nimblegen Microarrays support.

A. Evidence for the 14522 *S. sclerotiorum* predicted genes:

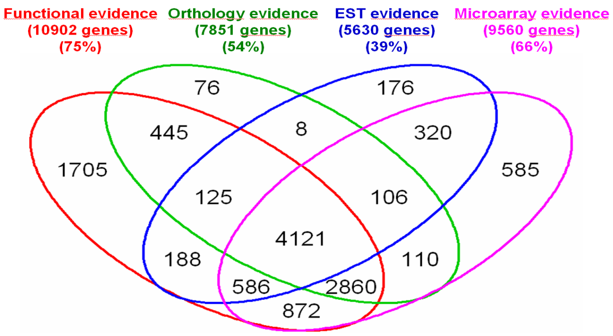

B. Evidence for the 16360 *B. cinerea* T4 predicted genes :

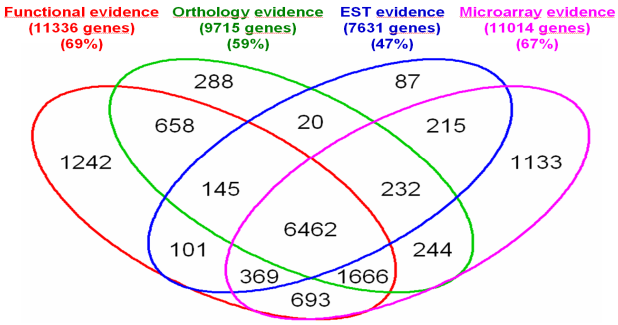

C. Evidence for the 16448 *B. cinerea* B05.10 predicted genes :

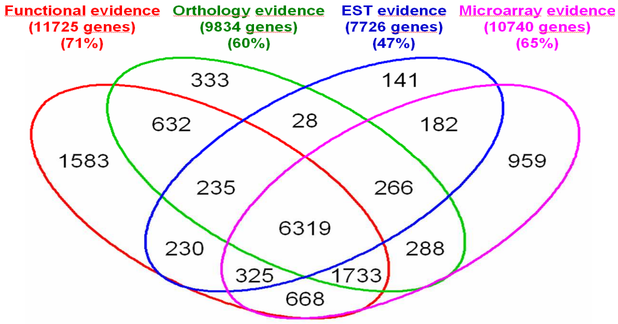

Supplement: Figure S3 — Evidence for accuracy of gene prediction in S. sclerotiorum (A), B. cinerea T4 (B) and B. cinerea B05.10 (C). Four types of evidence were combined: (i) Functional annotation (at least one domain/motif), (ii) Orthology between S. sclerotiorum, B. cinerea and other fungi, (iii) EST support, (iv) Nimblegen Microarrays support. (PDF) [file pgen.1002230.s003.pdf]
