# Supplementary material for: Genomic Analysis of the Necrotrophic Fungal Pathogens Sclerotinia sclerotiorum and Botrytis cinerea
Source: PLoS Genet. 2011 Aug 18;7(8):e1002230. doi: 10.1371/journal.pgen.1002230 (PMC3158057; doi:10.1371/journal.pgen.1002230)

Figure S4

Gene length distribution before and after filtering of dubious gene predictions.
Histogram of gene length for *B. cinerea* (Bc) B05.10 and T4 initial and filtered (high confidence) genes, *S. sclerotiorum* (Ss) initial and filtered (high confidence) genes, *Neurospora crassa* (Nc) genes, and *Gibberella zeae* (Gz) genes.

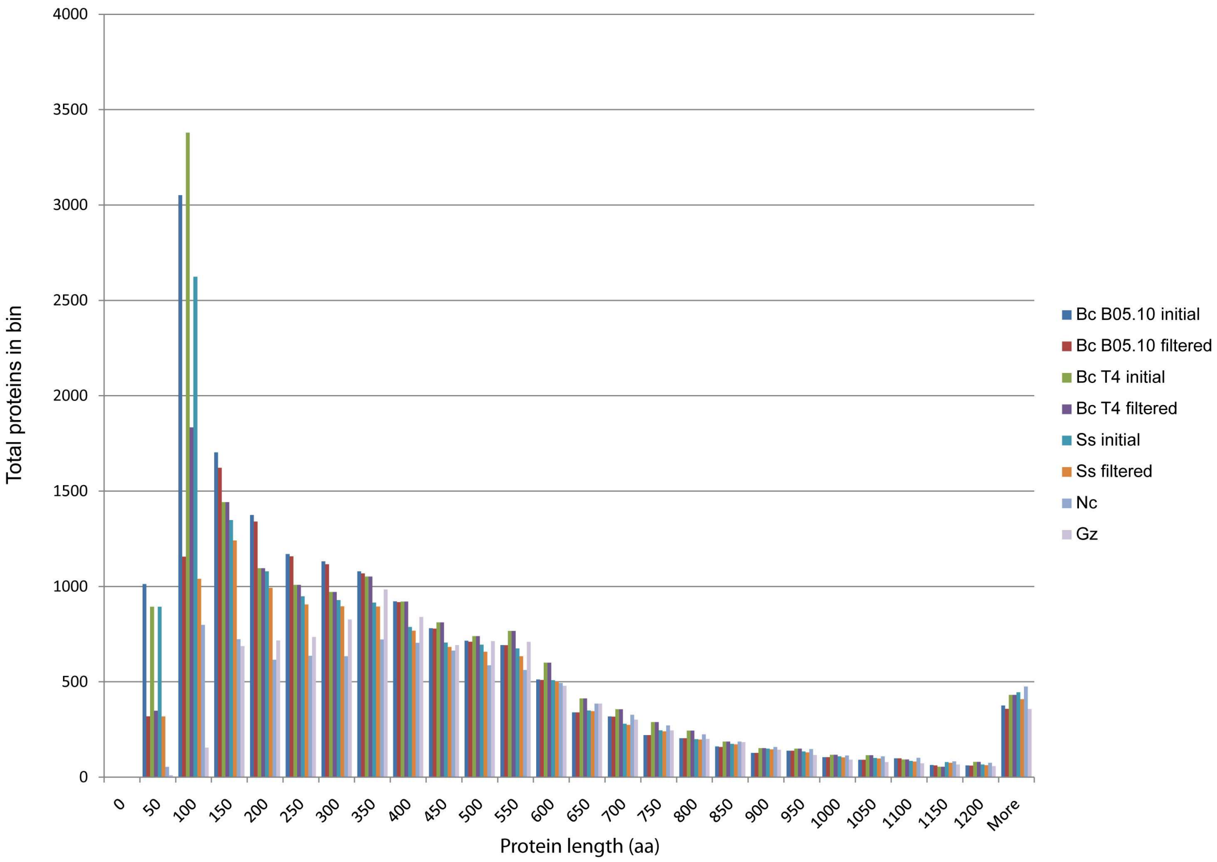

Supplement: Figure S4 — Gene length distribution before and after filtering of dubious gene predictions. Histogram of gene length for B. cinerea (Bc) B05.10 and T4 initial and filtered (high confidence) genes, S. sclerotiorum (Ss) initial and filtered (high confidence) genes, N. crassa (Nc) genes, and G. zeae (Gz) genes. (PDF) [file pgen.1002230.s004.pdf]
