# Supplementary material for: Genomic Analysis of the Necrotrophic Fungal Pathogens Sclerotinia sclerotiorum and Botrytis cinerea
Source: PLoS Genet. 2011 Aug 18;7(8):e1002230. doi: 10.1371/journal.pgen.1002230 (PMC3158057; doi:10.1371/journal.pgen.1002230)

Figure S5

Dotplot view of syntenic regions between genomes.

Syntenic regions were determined by DAGchainer for each pair of genomes; for *B. cinerea* scaffolds are shown sorted by size and for *S. sclerotiorum* chromosomes are shown in numerical order, with ChrR last. Lines are colored red or blue to highlight positive (red) or negative (blue) strand orientation of regions for the genome on the y-axis.

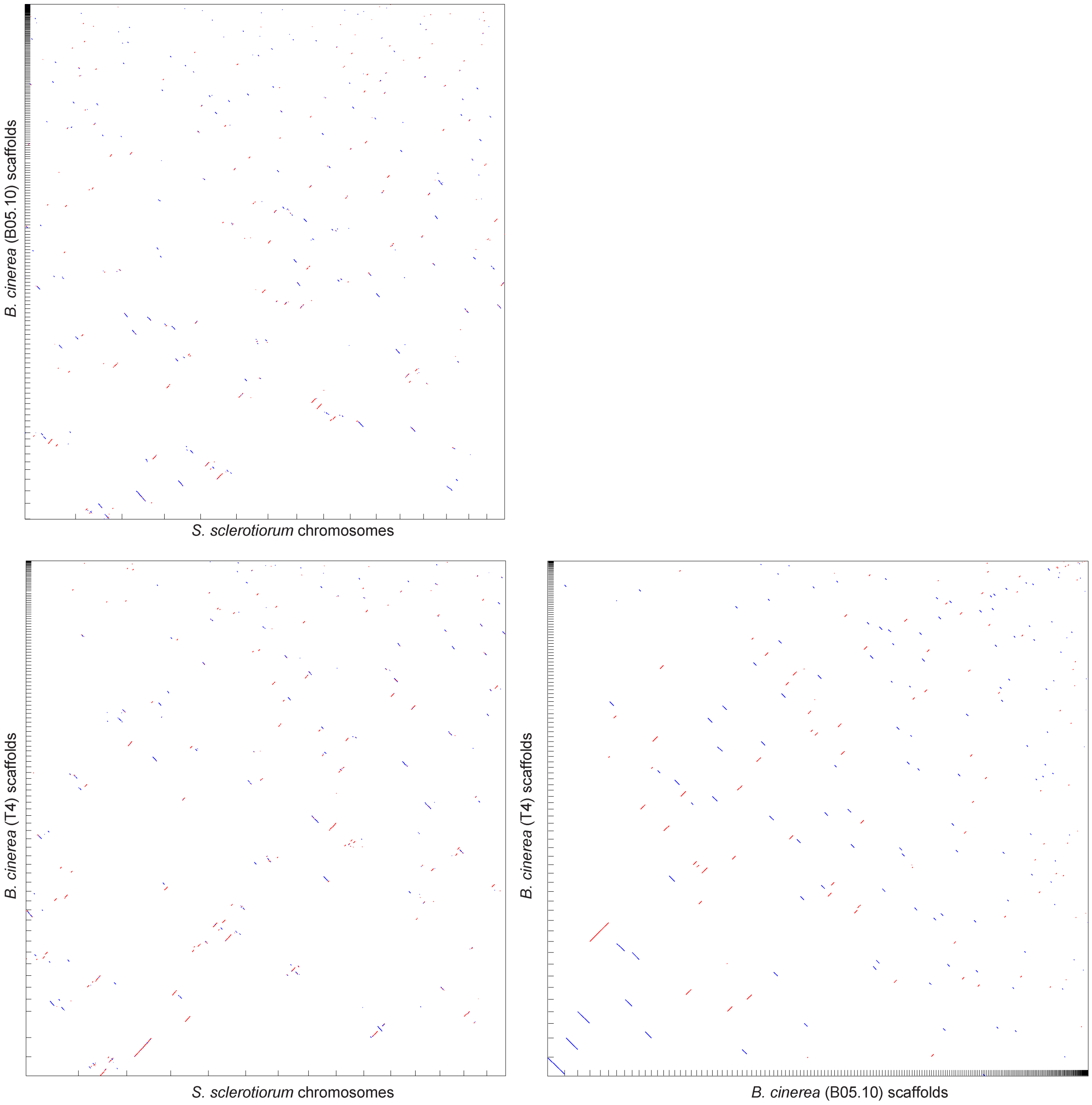

Supplement: Figure S5 — Dotplot view of syntenic regions between genomes. (PDF) [file pgen.1002230.s005.pdf]
