# Supplementary material for: Genomic Analysis of the Necrotrophic Fungal Pathogens Sclerotinia sclerotiorum and Botrytis cinerea
Source: PLoS Genet. 2011 Aug 18;7(8):e1002230. doi: 10.1371/journal.pgen.1002230 (PMC3158057; doi:10.1371/journal.pgen.1002230)

Figure S6

Diversity of ScTIR1 genomic copies.

Full length genomic copies of ScTIR1 were retrieved from the genome using REPET and aligned (clustalW). The aligment was used to construct a phylogenetic tree using Maximum parsimony. Copies with identical sequences are encircled.

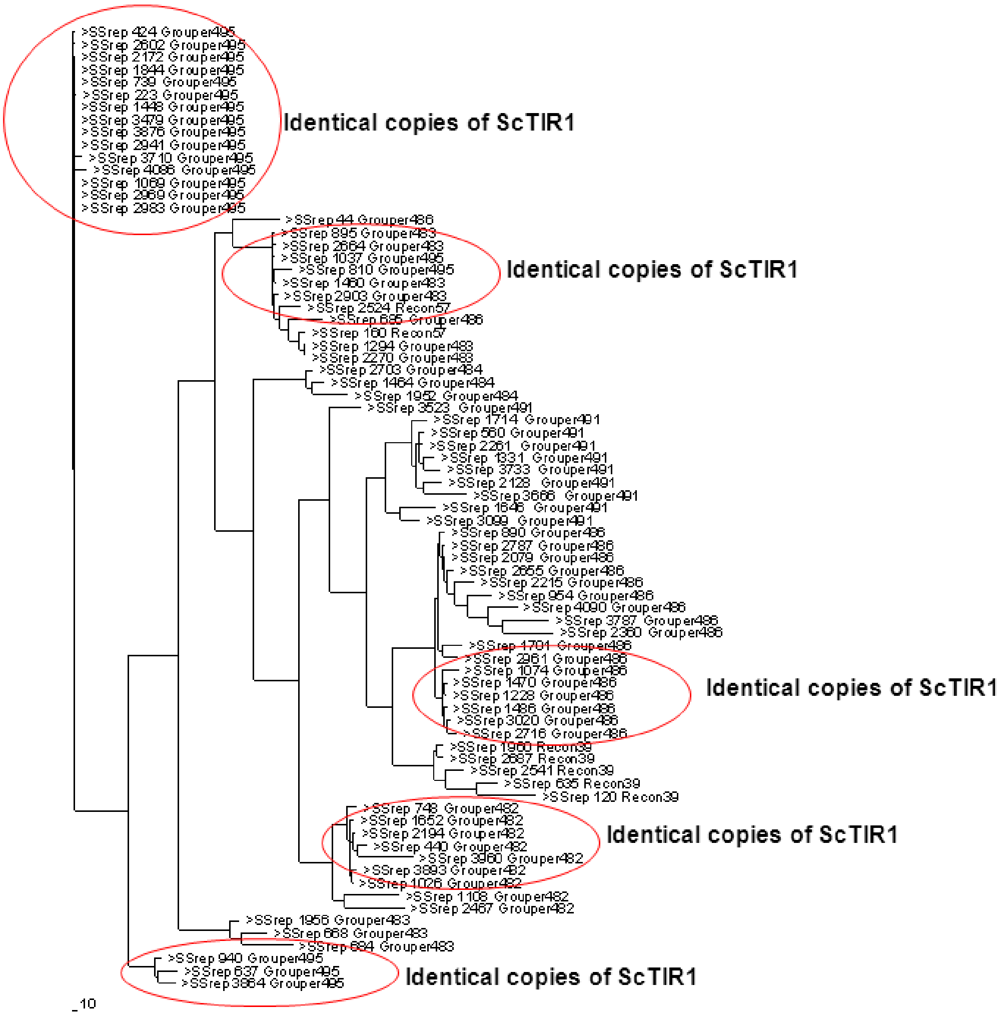

Supplement: Figure S6 — Diversity of ScTIR1 genomic copies. Full-length genomic copies of ScTIR1 were retrieved from the genome using REPET and aligned (clustalW). The aligment was used to construct a phylogenetic tree using Maximum parsimony. Identical sequences copies are circled. (PDF) [file pgen.1002230.s006.pdf]
