# Supplementary material for: Genomic Analysis of the Necrotrophic Fungal Pathogens Sclerotinia sclerotiorum and Botrytis cinerea
Source: PLoS Genet. 2011 Aug 18;7(8):e1002230. doi: 10.1371/journal.pgen.1002230 (PMC3158057; doi:10.1371/journal.pgen.1002230)

Figure S7

Gene count per OrthoMCL family for each species.

Genes were clustered into families using OrthoMCL for *S. sclerotiorum*, both strains of *B. cinerea*, and 7 other fungal genomes. The x-axis bins measure the number of genes per family for each species, where orphan genes not in families are in the 0 bin, genes in single copy in a family are in the 1 bin, and paralogous genes of 2 or more in a family are in the higher bins. The y-axis varies between the subplots, and counts the total number of genes in each bin per species.

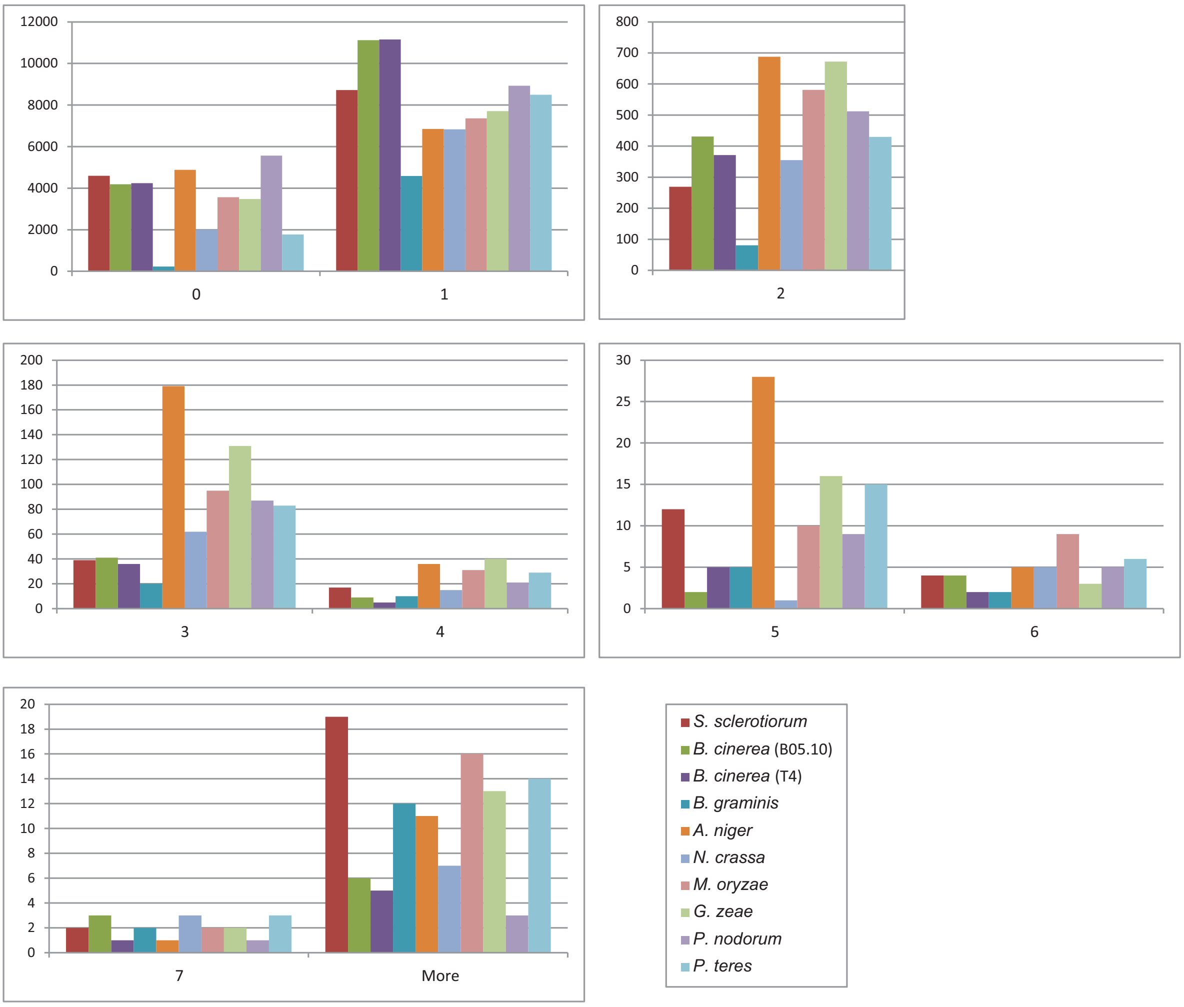

Supplement: Figure S7 — Gene count per OrthoMCL family for each species. Genes were clustered into families using OrthoMCL for S. sclerotiorum, both strains of B. cinerea, and 12 other fungal genomes. The x-axis bins measure the number of genes per family for each species, where orphan genes not in families are in the 0 bin, genes in single copy in a family are in the 1 bin, and paralogous genes of 2 or more in a family are in the higher bins. The y-axis varies between the subplots, and counts the total number of genes in each bin per species. (PDF) [file pgen.1002230.s007.pdf]
