# Supplementary material for: Genomic Analysis of the Necrotrophic Fungal Pathogens Sclerotinia sclerotiorum and Botrytis cinerea
Source: PLoS Genet. 2011 Aug 18;7(8):e1002230. doi: 10.1371/journal.pgen.1002230 (PMC3158057; doi:10.1371/journal.pgen.1002230)

Figure S8

Phylogeny of apoptosis-associated BIR1-homologs in fungi and yeasts.

Dark gray boxes indicate homologs with two BIR-domains in a single protein, light gray boxes indicate homologs with one BIR-domain in a single protein, white boxes indicate homologs lacking a BIR-domain.

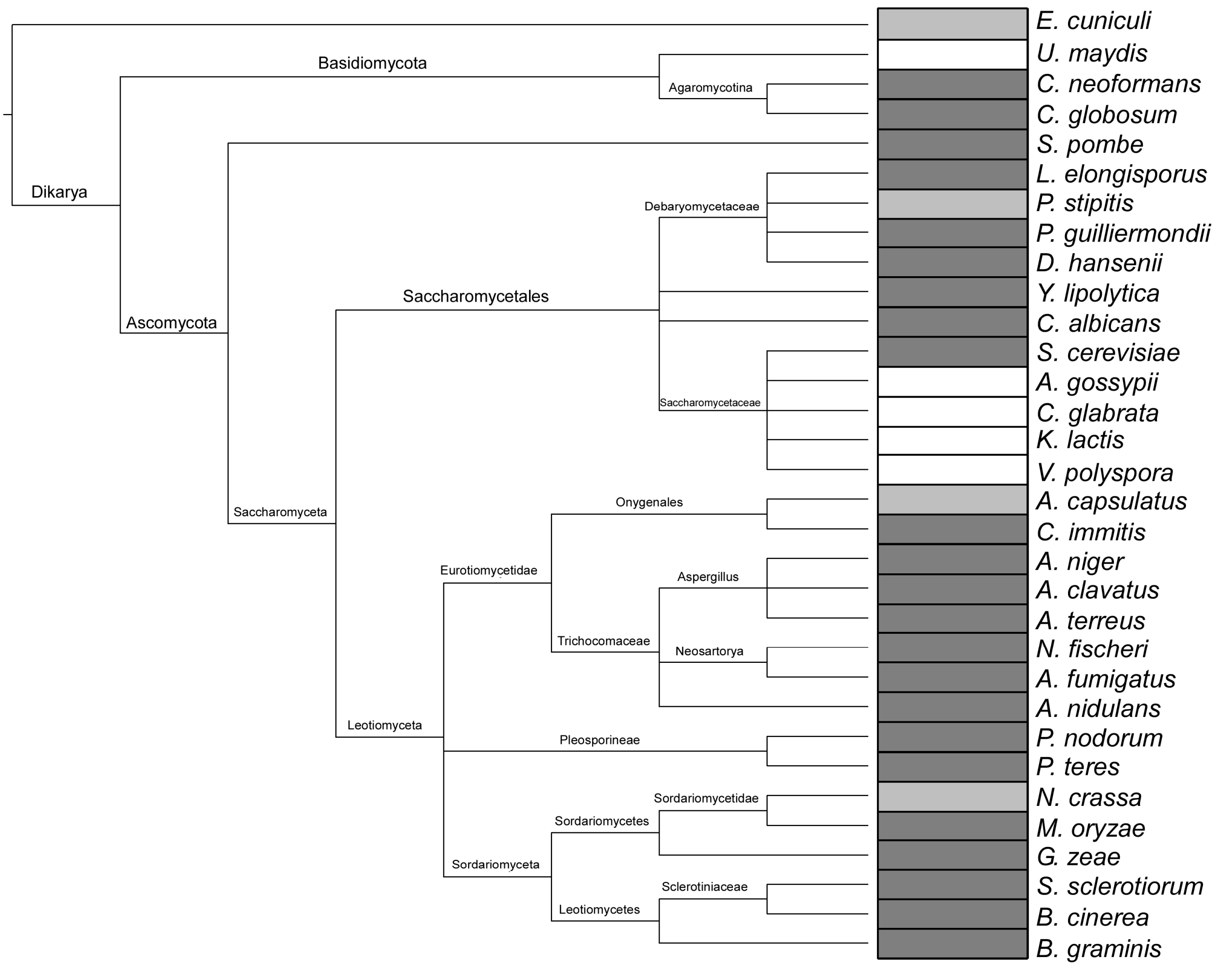

Supplement: Figure S8 — Phylogeny of apoptosis-associated BIR1-homologs in fungi and yeasts. Dark gray boxes indicate homologs with two BIR-domains in a single protein, light gray boxes indicate homologs with one BIR-domain in a single protein, white boxes indicate homologs lacking a BIR-domain. (PDF) [file pgen.1002230.s008.pdf]
