# Supplementary material for: Genomic Analysis of the Necrotrophic Fungal Pathogens Sclerotinia sclerotiorum and Botrytis cinerea
Source: PLoS Genet. 2011 Aug 18;7(8):e1002230. doi: 10.1371/journal.pgen.1002230 (PMC3158057; doi:10.1371/journal.pgen.1002230)

Figure S9

Growth of *S. sclerotiorum* and *B. cinerea* and five other fungi on monosaccharides and simple or complex plant polysaccharides.

Blumeria graminis is an obligate biotroph, and therefore could not be included in the assay.

Pyrenophora teres f. teres was not tested.

More extensive growth profiles for these and other fungi can be found at www.fung-growth.org.

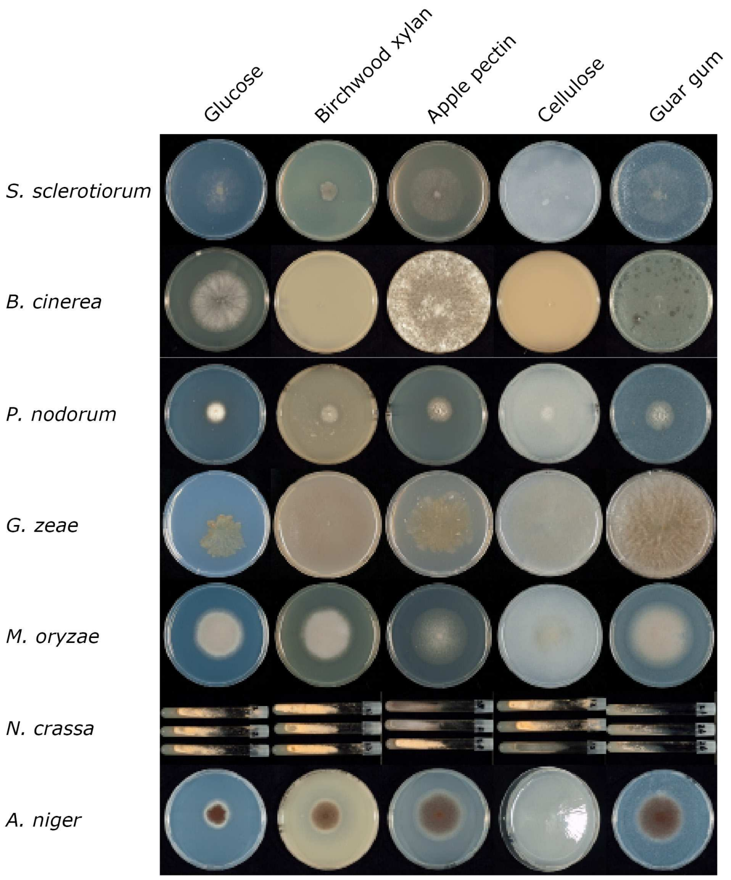

Supplement: Figure S9 — Growth of S. sclerotiorum and B. cinerea and five other Ascomycetes on monosaccharides and simple or complex plant polysaccharides. More extensive growth profiles for these and other fungi can be found at www.fung-growth.org. (PDF) [file pgen.1002230.s009.pdf]
