# Supplementary material for: Genomic Analysis of the Necrotrophic Fungal Pathogens Sclerotinia sclerotiorum and Botrytis cinerea
Source: PLoS Genet. 2011 Aug 18;7(8):e1002230. doi: 10.1371/journal.pgen.1002230 (PMC3158057; doi:10.1371/journal.pgen.1002230)

Figure S10

A. Representation of *S. sclerotiorum* EST libraries (percentage) corresponding to the 63810 clones.

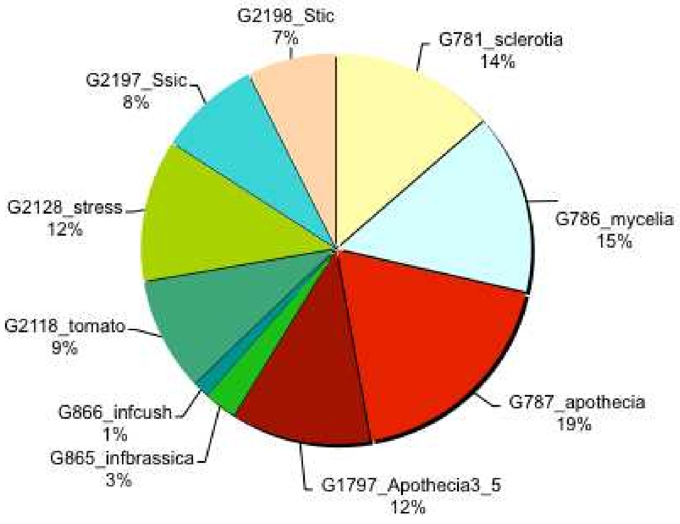

B. Representation of the *B. cinerea* libraries (percentage) corresponding to the 67625 clones (71238 ESTs) mapped and clustered on the *B. cinerea* T4 genome before assembling in contigs.

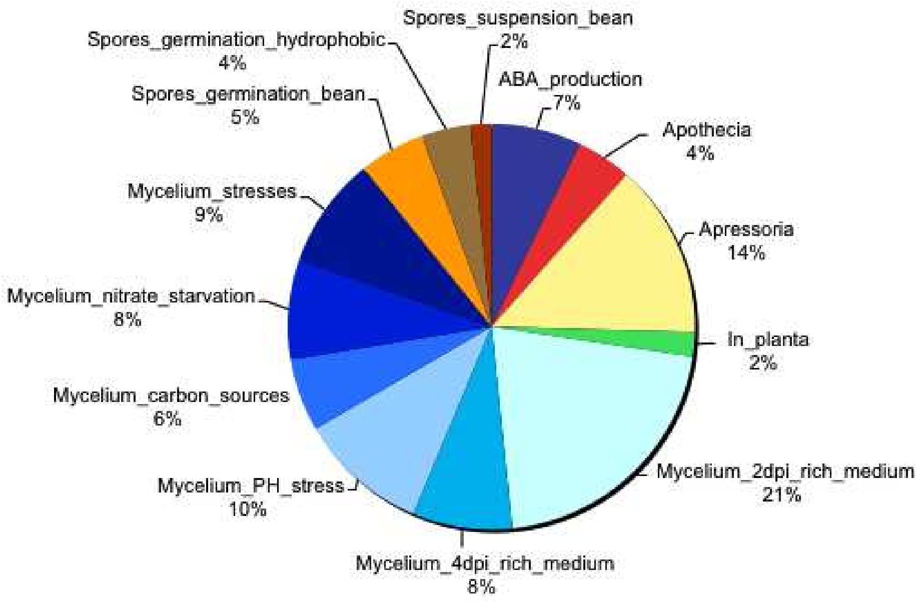

Supplement: Figure S10 — (A). Representation of S. sclerotiorum EST libraries (percentage) corresponding to the 63,810 clones. (B). Representation of each of the B. cinerea libraries (percentage) corresponding to the 67,625 clones (71,238 ESTs) mapped and clustered on the B. cinerea T4 genome before assembling in EST contigs. (PDF) [file pgen.1002230.s010.pdf]
