# Supplementary material for: Genomic Analysis of the Necrotrophic Fungal Pathogens Sclerotinia sclerotiorum and Botrytis cinerea
Source: PLoS Genet. 2011 Aug 18;7(8):e1002230. doi: 10.1371/journal.pgen.1002230 (PMC3158057; doi:10.1371/journal.pgen.1002230)

Figure S11

Localization on the *S. sclerotiorum* genome map of genes encoding secondary metabolism key enzymes.

Regions that show synteny with *B. cinerea* are indicated in red. SB1 to SB19 indicate genes that are shared between the two species whereas S1 to S9 indicate *S. sclerotiorum*-specific genes (see Table S23 for more details). SB1: PHS1,SB2: PKS1, SB3: PKS2, SB4: PKS8, SB5: PKS9, SB6: PKS10, SB7: PKS12, SB8:PKS13, SB9: PKS18, SB10: PKS21, SB11: CHS1, SB12: PKS6, SB13: NRPS1 , SB14:NRPS2, SB15: NRPS3, SB16: NRPS4, SB17: NRPS5, SB18: NRPS6, SB19:DMATS1, S1: SsFUS1, S2: SsPKS3, S3: SsPKS4, S4: SsPKS5, S5:SsPKS7, S6: SsPKS11, S7: SsPKS14, S8: SsPKS15, S9: SsPKS16.

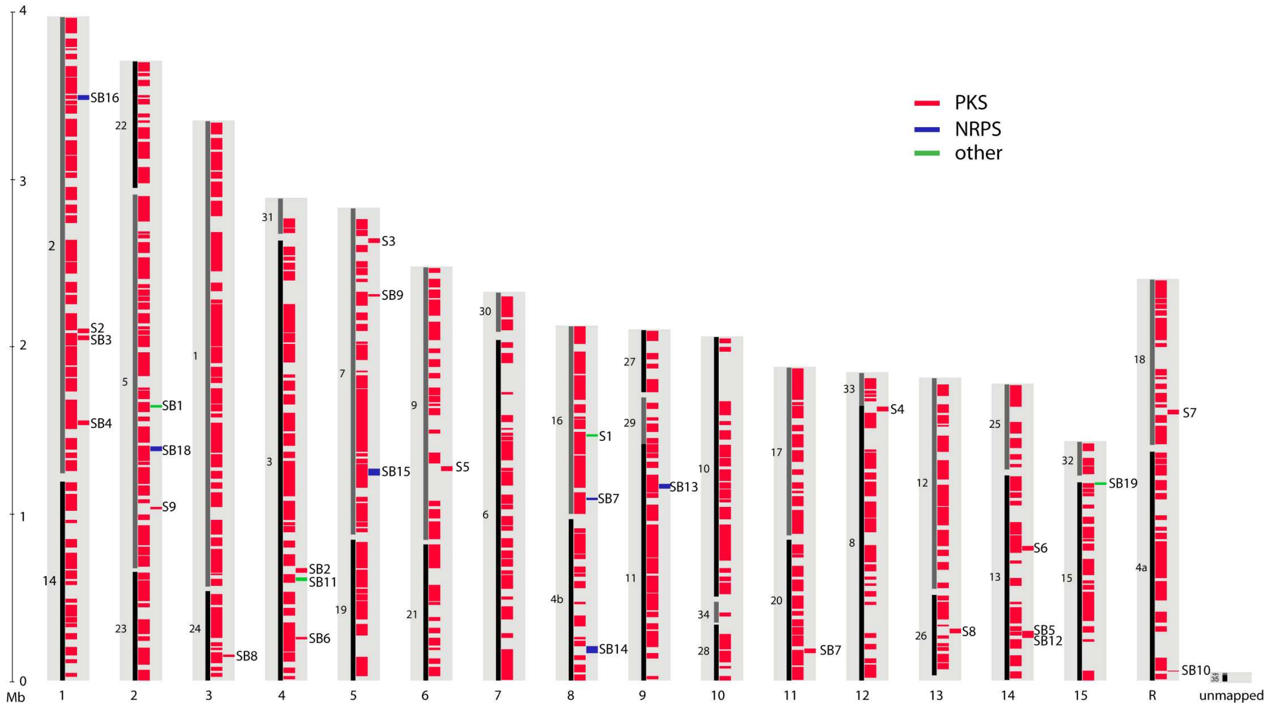

Supplement: Figure S11 — Localization on the S. sclerotiorum genome map of genes encoding secondary metabolism key enzymes. Regions that show synteny with B. cinerea are indicated in red. SB1 to SB19 indicate genes that are shared between the two species whereas S1 to S9 indicate S. sclerotiorum-specific genes (see Table S23 for more details). SB1: PHS1,SB2: PKS1, SB3: PKS2, SB4: PKS8, SB5: PKS9, SB6: PKS10, SB7: PKS12, SB8:PKS13, SB9: PKS18, SB10: PKS21, SB11: CHS1, SB12: PKS6, SB13: NRPS1, SB14:NRPS2, SB15: NRPS3, SB16: NRPS4, SB17: NRPS5, SB18: NRPS6, SB19:DMATS1, S1: SsFUS1, S2: SsPKS3, S3: SsPKS4, S4: SsPKS5, S5:SsPKS7, S6: SsPKS11, S7: SsPKS14, S8: SsPKS15, S9: SsPKS16. (PDF) [file pgen.1002230.s011.pdf]
