# Supplementary material for: Genomic Analysis of the Necrotrophic Fungal Pathogens Sclerotinia sclerotiorum and Botrytis cinerea
Source: PLoS Genet. 2011 Aug 18;7(8):e1002230. doi: 10.1371/journal.pgen.1002230 (PMC3158057; doi:10.1371/journal.pgen.1002230)
Supplement: Table S1 — Linkage groups in B. cinerea based on the analysis of 68 progeny from a cross. (PDF) [file pgen.1002230.s012.pdf]

Table S1

Linkage groups based on the analysis of 68 progeny from a cross between *B. cinerea* strains T4 and 32.

The linkage groups have been ordered to follow roughly the structural map. Coordinates of the genetic markers on the supercontig are given.

Linkage group	Marker	Distance	Supercontig position	Beginning	Ending
group7	Bc361	31.8 cM	bt4_SuperContig_77_1	53457	53692
group7	Bc28	9.3 cM	bt4_SuperContig_50_1	895272	895621
group7	P3SNP22	17.3 cM	bt4_SuperContig_50_1	879278	
group7	Bc136		bt4_SuperContig_50_1	868902	869078
group2	Bc15	26.6 cM	bt4_SuperContig_50_1	61083	61252
group2	Bc219	15.2 cM	bt4_SupSuperContig_213_271_1	233081	232772
group2	P3SNP45		bt4_SupSuperContig_213_271_1	296266	
group33	Bc380	29.7 cM	bt4_SuperContig_173_1	335263	335451
group33	P1SNP32		bt4_SuperContig_173_1	274926	
group36	P2SNP22	13.1 cM	bt4_SuperContig_22_1	788786	
group36	P3SNP10	36.1 cM	bt4_SuperContig_22_1	668078	
group36	P2SNP5		bt4_SuperContig_22_1	436265	
group4	Bc126	21.4 cM	bt4_SuperContig_22_1	2163	2447
group4	Bc71	3.4 cM	bt4_SuperContig_289_1	26954	26769
group4	P2SNP17	20.2 cM	bt4_SuperContig_289_1	58392	
group4	P3SNP36	7.8 cM	bt4_SuperContig_289_1	341327	
group4	BC379	14.9 cM	bt4_SuperContig_289_1	384598	384786
group4	P3SNP40	32.3 cM	bt4_SupSuperContig_101_375_327_1	146576	
group4	Bc171	8.9 cM	bt4_SupSuperContig_101_375_327_1	453427	453427
group4	P3SNP42	36.8 cM	bt4_SupSuperContig_101_375_327_1	547742	
group4	Bc135	3.0 cM	bt4_SupSuperContig_101_375_327_1	837163	836824
group4	Bc63		bt4_SuperContig_87_1	39974	40204
group35	P3SNP4	10.2 cM	bt4_SuperContig_3_1	466315	
group35	P3SNP3	20.8 cM	bt4_SuperContig_3_1	310745	
group35	Bc81	24.3 cM	bt4_SuperContig_3_1	144911	145235
group35	Bc377	11.2 cM	bt4_SuperContig_3_1	1614	1827
group35	P1SNP29	28.4 cM	bt4_SuperContig_153_1	19253	
group3	P2SNP14	22.6 cM	bt4_SuperContig_153_1	394348	
group3	Bc22	39.7 cM	bt4_SuperContig_153_1	605093	605249
group3	Bc308	8.3 cM	bt4_SupSuperContig_106_216_1	26881	27119
group3	Bc397	1.5 cM	bt4_SuperContig_264_1	7242	7457
group3	Bc409_1	8.4 cM	bt4_SuperContig_264_1	7242	7440
group3	Bc86	25.1 cM	bt4_SupSuperContig_106_216_1	119397	119726
group3	P2SNP33	17.3 cM	bt4_SupSuperContig_106_216_1	277496	
group3	Bc307		bt4_SupSuperContig_106_216_1	410030	410198
group10	Bc66	13.4 cM	bt4_SuperContig_237_1	142939	142644
group10	Bc291	6.3 cM	bt4_SupSuperContig_79_291_1	5676	5841
group10	P1SNP34	6.3 cM	bt4_SupSuperContig_79_291_1	97757	
group10	Bc2	9.9 cM	bt4_SuperContig_329_1	35172	35332
group10	Bc330		bt4_SuperContig_53_1	5907	6109

group14	P3SNP43	12.6 cM	bt4_SupSuperContig_110r_56_1	270 494	
group14	Bc309	9.8 cM	bt4_SupSuperContig_110r_56_1	314359	314586
group14	Bc129	1.5 cM	bt4_SupSuperContig_246_31_4_1	85048	85221
group14	MAT	1.6 cM	bt4_SupSuperContig_246_31_4_1	30 143	40423
group14	P2SNP25	12.3 cM	bt4_SupSuperContig_246_31_4_1	34 880	
group24	P3SNP46	14.4 cM	bt4_SupSuperContig_246_31_4_1	874 719	
group24	P3SNP16	4.9 cM	bt4_SuperContig_34_1	149 153	
group24	P3SNP17	24.0 cM	bt4_SuperContig_34_1	311 289	
group24	Bc58		bt4_SuperContig_34_1	542 586	542 420
group6	P1SNP14	25.0 cM	bt4_SuperContig_34_1	840632	
group6	Bc300	24.4 cM	bt4_SuperContig_34_1	1 343 146	1 343 425
group6	P3SNP5	13.0 cM	bt4_SuperContig_9_1	222 215	
group6	Bc141	34.7 cM	bt4_SuperContig_86_1	2 276	1 799
group6	P3SNP28	20.1 cM	bt4_SuperContig_86_1	211 082	
group6	Bc376	13.5 cM	bt4_SuperContig_321_1	55356	55599
group6	Bc249	6.4 cM	bt4_SuperContig_321_1	8 177	8 807
group6	Bc184		bt4_SuperContig_19_1	16 204	15 725
group1	P2SNP34	7.5 cM	bt4_SuperContig_143_1	68819	
group1	Bc51	27.0 cM	bt4_SuperContig_143_1	127717	127926
group1	P1SNP28	26.0 cM	bt4_SuperContig_143_1	270245	
group1	Bc75	13.7 cM	bt4_SuperContig_174_1	80060	80238
group1	P2SNP40	0.0 cM	bt4_SuperContig_174_1	36829	
group1	Bc143	35.7 cM	bt4_SuperContig_103_1	14439	14116
group1	P1SNP24	11.8 cM	bt4_SuperContig_103_1	233550	
group1	Bc12	35.0 cM	bt4_SuperContig_103_1	323984	324105
group1	Bc294	29.8 cM	bt4_SupSuperContig_114_320_122_1	660338	660584
group1	Bc296	8.3 cM	bt4_SuperContig_29_1	590497	590735
group1	Bc395		bt4_SuperContig_29_1	281561	281750
group30	Bc254	20.5 cM	bt4_SuperContig_29_1	7555	8040
group30	P1SNP31	8.1 cM	bt4_SuperContig_171_1	33550	
group30	P3SNP9	10.2 cM	bt4_SuperContig_171_1	41051	
group30	Bc312	15.7 cM	bt4_SuperContig_17_1	44017	44249
group30	Bc332	38.5 cM	bt4_SuperContig_17_1	70034	70237
group30	Bc349		bt4_SuperContig_171_1	65561	65770
group19	P1SNP10	14.3 cM	bt4_SuperContig_30_1	132086	
group19	Bc223P	26.3 cM	bt4_SuperContig_30_1	154 984	154 239
group19	Bc306	10.5 cM	bt4_SuperContig_30_1	453698	453886
group19	Bc353	8.0 cM	bt4_SuperContig_160_1	89614	89854
group19	P3SNP32	8.0 cM	bt4_SuperContig_160_1	74135	
group19	Bc288	18.4 cM	bt4_SupSuperContig_36_28_1	15597	15722
group19	P2SNP11	6.7 cM	bt4_SupSuperContig_36_28_1	255919	
group19	P2SNP16		bt4_SupSuperContig_36_28_1	453170	
group22	Bc321	18.1 cM	bt4_SuperContig_379_1	25717	25911
group22	Bc276		bt4_SupSuperContig_67_197_1	133097	133439
group28	Bc277	11.0 cM	bt4_SupSuperContig_67_197_1	406883	407387
group28	P1SNP25	34.5 cM	bt4_SuperContig_104_1	103519	
group28	Bc54	28.5 cM	bt4_SuperContig_104_1	169 766	169 095
group28	Bc374	40.5 cM	bt4_SuperContig_104_1	154199	154423
group26	Bc297	28.3 cM	bt4_SuperContig_0_1	116222	116471
group26	Bc250	32.7 cM	bt4_SuperContig_0_1	421 918	422 667
group26	Bc382	34.6 cM	bt4_SuperContig_39_1	61952	62252
group26	Bc322	23.1 cM	bt4_SuperContig_39_1	187329	187134
group26	Bc385	6.3 cM	bt4_SuperContig_39_1	202883	203043
group26	Bc384	6.4 cM	bt4_SuperContig_39_1	205549	205744
group26	Bc373	8.2 cM	bt4_SuperContig_164_1	453	642
group26	Bc347		bt4_SuperContig_72_1	8265	8749

group21	P1SNP22	6.6 cM	bt4_SuperContig_100_1	107953	
group21	Bc279	29.1 cM	bt4_SuperContig_100_1	32647	32911
group21	Bc360	32.3 cM	bt4_SuperContig_38_1	13897	14146
group21	Bc261	31.4 cM	bt4_SuperContig_10_1	4793	5030
group21	P1SNP4		bt4_SuperContig_10_1	159943	
group27	Bc301	14.3 cM	bt4_SuperContig_10_1	1443997	1444226
group27	P3SNP8	5.6 cM	bt4_SuperContig_11_1	90883	
group27	Bc278		bt4_SuperContig_48_1	37377	37610
group15	Bc335	32.1 cM	bt4_SuperContig_209_1	46183	46386
group15	Bc342	13.1 cM	bt4_SuperContig_35_1	12603	12832
group15	Bc389	5.1 cM	bt4_SuperContig_2_1	2339	2784
group15	Bc390	7.0 cM	bt4_SuperContig_2_1	56309	56671
group15	P2SNP50	8.9	bt4_SuperContig_2_1	65 699	
group15	Bc246		bt4_SuperContig_2_1	97136	97298
group17	P2SNP7	16.2 cM	bt4_SuperContig_346_1	48591	
group17	P2SNP26	14.9 cM	bt4_SuperContig_5_1	317 453	
group17	Bc233P	27.4 cM	bt4_SuperContig_5_1	309 371	309 056
group17	Bc348	10.4 cM	bt4_SuperContig_33_1	71745	71977
group17	P1SNP37	28.3 cM	bt4_SuperContig_313_1	110258	
group17	Bc369		bt4_SuperContig_97_1	135062	135551
group13	Bc24	13.4 cM	bt4_SuperContig_111_1	99 258	98 614
group13	P3SNP30	14.8 cM	bt4_SuperContig_111_1	75873	
group13	Bc343	8.5 cM	bt4_SuperContig_219_1	85084	85287
group13	P1SNP33		bt4_SuperContig_219_1	60123	
group12	Bc255	3.2 cM	bt4_SuperContig_277_1	88906	89941
group12	P2SNP20	36.8 cM	bt4_SuperContig_277_1	92476	
group32	P2SNP19	17.3 cM	bt4_SuperContig_277_1	282478	
group32	P3SNP35	17.3 cM	bt4_SuperContig_277_1	492059	
group32	P1SNP36	12.2 cM	bt4_SuperContig_277_1	698169	
group32	Bc251P	13.5 cM	bt4_SuperContig_277_1	799884	801018
group32	Bc339		bt4_SuperContig_66_1	62476	62724
group18	P3SNP1	19.1 cM	bt4_SupSuperContig_8_105_1	534506	
group18	Bc268	8.0 cM	bt4_SupSuperContig_8_105_1	667685	669071
group18	Bc351		bt4_SuperContig_177_1	25076	25305
group29	Bc314	12.1 cM	bt4_SuperContig_58_1	3986	4203
group29	Bc232		bt4_SuperContig_58_1	155780	155944
group33	Bc337	29.7 cM	bt4_SupSuperContig_62_54_1	555826	556053
group33	Bc334		bt4_SuperContig_99_1	392350	392567
group31	Bc324	16.1 cM	bt4_SuperContig_59_1	203360	203576
group31	Bc340		bt4_SuperContig_27_1	131244	131427
group9	Bc319	28.4 cM	bt4_SuperContig_78_1	5310	5509
group9	Bc44		bt4_SuperContig_144_1	216038	216240
group20	Bc393	19.1 cM	bt4_SuperContig_151_1	200581	200973
group20	Bc338	9.7 cM	bt4_SuperContig_23_1	39 232	39 469
group20	Bc270		bt4_SuperContig_51_1	35 317	35 540
group11	Bc252	3.1 cM	bt4_SupSuperContig_210_20_1	551149	551387
group11	Bc292		bt4_SupSuperContig_210_20_1	706670	706844
group8	Bc191	3.1 cM	bt4_SupSuperContig_43_186_1	646766	646933
group8	Bc336		bt4_SuperContig_169_1	126148	126381
group25	Bc293	12.6 cM	bt4_SupSuperContig_114_320_122_1	149015	149251
group25	P2SNP45		bt4_SupSuperContig_114_320_122_1	180431	

group5	Bc188	18.0 cM	bt4_SuperContig_32_1	527335	527523
group5	Bc218	5.0 cM	bt4_SuperContig_32_1	174669	174845
group5	Bc387		bt4_SuperContig_32_1	131758	132853
group16	Bc327	13.7 cM	bt4_SuperContig_108_1	125612	125839
group16	Bc142	18.5 cM	bt4_SuperContig_108_1	197363	197170
group16	Bc399	17.7 cM	bt4_SuperContig_312_1	19908	20141
group16	Bc280	26.7 cM	bt4_SuperContig_25_1	365216	365216
group16	Bc375	3.3 cM	bt4_SuperContig_83_1	60592	60684
group16	Bc253	4.7 cM	bt4_SuperContig_29_1	670057	670298
group16	P2SNP31	26.3 cM	bt4_SuperContig_29_1	665551	
group16	Bc394	3.3 cM	bt4_SuperContig_296_1	72615	72784
group16	P2SNP9	18.4 cM	bt4_SuperContig_296_1	103279	
group16	Bc284		bt4_SupSuperContig_145_323r_1	43636	43844
group23	P2SNP48	28.0 cM	bt4_SuperContig_131_1	181193	
group23	Bc79		bt4_SuperContig_131_1	119259	119455
