# Supplementary material for: Genomic Analysis of the Necrotrophic Fungal Pathogens Sclerotinia sclerotiorum and Botrytis cinerea
Source: PLoS Genet. 2011 Aug 18;7(8):e1002230. doi: 10.1371/journal.pgen.1002230 (PMC3158057; doi:10.1371/journal.pgen.1002230)
Supplement: Table S2 — Genome summary of Pezizomycotina fungi. (PDF) [file pgen.1002230.s013.pdf]

Table S2

Genome summary of Pezizomycotina fungi.

The number of genes predicted in *S. sclerotiorum* strain 1980 (14,522 genes) and *B. cinerea* (16,448 and 16,360 genes for strains B05.10 and T4, respectively) is larger than those of other sequenced fungi in Pezizomycotina (average 11,154 genes).

Species	Strain	Genome size (Mb)	Genes	Data source
Aspergillus fumigatus	Af293	28.8	9,630	JCVI
Aspergillus nidulans	FGSC A4	30.1	10,560	Broad Institute
Aspergillus niger	ATCC1015	34.9	11,197	DOE JGI
Aspergillus oryzae	RIB40	37.1	12,079	NITE
Aspergillus terreus	NIH2624	29.3	10,406	Broad Institute
Botrytis cinerea	B05.10	42.7	16,448	Broad Institute
Botrytis cinerea	T4	39.5	16,360	Genoscope
Chaetomium globosum	CBS 148.51	34.9	11,124	Broad Institute
Coccidioides immitis	RS	29.0	9,757	Broad Institute
Fusarium oxysporum f. sp. *lycopersici*	4287	61.4	17,735	Broad Institute
Fusarium verticillioides	7600	41.8	14,179	Broad Institute
Gibberella zeae	PH-1	36.5	13,321	Broad Institute
Histoplasma capsulatum	NAm1	33.0	9,248	Broad Institute
Magnaporthe oryzae	70-15	41.7	11,054	Broad Institute
Microsporum gypseum	CBS118893	23.3	8,907	Broad Institute
Nectria haematococca	77-13-4	54.4	15,707	DOE JGI
Neosartorya fischeri	NRRL181	32.6	10,407	JCVI
Neurospora crassa	OR74A	41.0	9,907	Broad Institute
Paracoccidioides brasiliensis	Pb18	30.0	8,741	Broad Institute
Phaeosphaeria nodorum	SN15	37.2	12,380	Broad Institute
Pyrenophora teres f. teres	0-1	41.2	11,799	Genbank
Pyrenophora tritici-repentis	Pt-1C-BFP	37.8	12,171	Broad Institute
Sclerotinia sclerotiorum	1980	38.3	14,522	Broad Institute
Talaromyces stipitatus	ATCC10500	35.7	12,449	JCVI
Trichoderma atroviride	IMI 206040	36.1	11,100	DOE JGI
Trichoderma reesei	QM6a	34.1	9,129	DOE JGI
Trichophyton equinum	CBS127.97	24.1	8,679	Broad Institute
Uncinocarpus reesii	UAMH 1704	22.3	7,798	Broad Institute
Verticillium dahliae	VdLs.17	33.8	10,535	Broad Institute
