# Supplementary material for: Genomic Analysis of the Necrotrophic Fungal Pathogens Sclerotinia sclerotiorum and Botrytis cinerea
Source: PLoS Genet. 2011 Aug 18;7(8):e1002230. doi: 10.1371/journal.pgen.1002230 (PMC3158057; doi:10.1371/journal.pgen.1002230)
Supplement: Table S3 — Duplicated blocks within the genomes of B. cinerea strain B05.10 and S. sclerotiorum. (PDF) [file pgen.1002230.s014.pdf]

Table S3

Duplicated blocks within the genome of *S. sclerotiorum* and *B. cinerea* strain B05.10. *B.cinerea* strain T4 lacks duplicated blocks. Blocks were identified using DAGchainer, using similar pairs identified by Blastp as input, and selecting regions of at least 4 genes. Regions may contain unaligned pairs; gene ranges are not necessarily inclusive.

Region 1 genes	Region 2 genes	Region 1 (bp)	Region 2 (bp)	Aligned pairs	Annotation
SS1G_05623-SS1G_05627	SS1G_11509-SS1G_11512	8,994	13,258	4	predicted proteins; HET-E-1; kinesin light chain 1 (Het); RT
SS1G_06637-SS1G_06640	SS1G_07150-SS1G_07154	10,872	8,668	4	predicted proteins; HET-E-1
SS1G_07990-SS1G_07998	SS1G_05532-SS1G_05538	11,741	11,269	6	predicted proteins; HET-E-1
SS1G_10115-SS1G_10120	SS1G_02418-SS1G_02423	12,435	14,219	4	predicted proteins; HET-E-1; RT
SS1G_08056-SS1G_08069	SS1G_11513-SS1G_11527	25,897	25,920	12	predicted proteins
SS1G_08022-SS1G_08026	SS1G_02835-SS1G_02831	8,437	11,233	5	predicted proteins; ATP-dependent chaperone
SS1G_09355-SS1G_09358	SS1G_12423-SS1G_12420	5,667	5,759	4	predicted proteins; serine/threonine protein kinase
SS1G_14499-SS1G_14505	SS1G_14508-SS1G_14514	14,606	17,146	6	rDNA region; spurious genes
BC1G_13988-BC1G_14000	BC1G_09697-BC1G_09713	44,806	38,400	8	predicted proteins
BC1G_02169-BC1G_02172	BC1G_12335-BC1G_12338	10,514	10,110	4	predicted proteins
