# Supplementary material for: Genomic Analysis of the Necrotrophic Fungal Pathogens Sclerotinia sclerotiorum and Botrytis cinerea
Source: PLoS Genet. 2011 Aug 18;7(8):e1002230. doi: 10.1371/journal.pgen.1002230 (PMC3158057; doi:10.1371/journal.pgen.1002230)
Supplement: Table S4 — Repeats identified de novo by cross-match alignments in S. sclerotiorum and B. cinerea genomes. (PDF) [file pgen.1002230.s015.pdf]

Table S4

Repeats identified *de novo* by cross-match alignment in *S. sclerotiorum* and *B. cinerea* genomes.

	S. sclerotiorum	*B. cinerea* B05.10	*B. cinerea* T4
Total repeats*	23,036	5,341	3,408
Average length	574	778	749
Repeat families#	1,984	1,484	1,122
Repeat regions^	2,694	1,778	1,415
Repeat region length (bp)	1,043	949	871
Total repeat (bp)	2,940,585	1,712,362	1,237,959
Assembly size (bp)	38,001,451	38,786,820	37,887,277
% repeat of assembly	7.7%	4.4%	3.3%

*Total repeats are the repeat units identified by cross_match
#Repeat families are identity-clustered repeat units
^Repeat regions merge overlapping repeat units into a single region
