# Supplementary material for: Genomic Analysis of the Necrotrophic Fungal Pathogens Sclerotinia sclerotiorum and Botrytis cinerea
Source: PLoS Genet. 2011 Aug 18;7(8):e1002230. doi: 10.1371/journal.pgen.1002230 (PMC3158057; doi:10.1371/journal.pgen.1002230)
Supplement: Table S5 — Repeats identified in S. sclerotiorum and B. cinerea genomes by RepeatMasker. (PDF) [file pgen.1002230.s016.pdf]

Table S5

Repeats identified by RepeatMasker.

	S. sclerotiorum	*B. cinerea* B05.10	*B. cinerea* T4
Simple repeats	0.73%	0.76%	0.88%
Low complexity	0.94%	0.65%	0.52%
All Transposons	2.07%	0.36%	0.28%
Total Repeats	3.74%	1.77%	1.68%
