# Supplementary material for: Genomic Analysis of the Necrotrophic Fungal Pathogens Sclerotinia sclerotiorum and Botrytis cinerea
Source: PLoS Genet. 2011 Aug 18;7(8):e1002230. doi: 10.1371/journal.pgen.1002230 (PMC3158057; doi:10.1371/journal.pgen.1002230)
Supplement: Table S6 — Number of TE families and TE copies identified in S. sclerotiorum and B. cinerea genomes using REPET. (PDF) [file pgen.1002230.s017.pdf]

Table S6

Number of TE families and TE copies identified in *S. sclerotiorum* and *B. cinerea* genomes using REPET.

	S. sclerotiorum		*B. cinerea* B05.10		*B. cinerea* T4	
	#Families	#Copies	#Families	#Copies	#Families	#Copies
LTR	5	2506	5	248	2	211
LINE	5	437	0	0	0	0
MITE	2	252	2	37	1	23
TIR	7	585	2	28	2	98
UNK	21	363	0	0	2	41
**Total**	**41**	**4143**	**9**	**313**	**10**	**373**

UNK: TE not assigned to known family.
