# Supplementary material for: Genomic Analysis of the Necrotrophic Fungal Pathogens Sclerotinia sclerotiorum and Botrytis cinerea
Source: PLoS Genet. 2011 Aug 18;7(8):e1002230. doi: 10.1371/journal.pgen.1002230 (PMC3158057; doi:10.1371/journal.pgen.1002230)
Supplement: Table S7 — Number of solo-LTRs and full-length copies of Gypsy/Copia-like retroelements identified in the genomes of S. sclerotiorum (Sclery) and B. cinerea (Boty). (PDF) [file pgen.1002230.s018.pdf]

Table S7

Number of solo LTRs and full length copies of Gypsy/Copia-like retroelements identified in the genomes of *S. sclerotiorum* (Sclery) and *B. cinerea* (Boty).

TE family	# solo-LTRs	# full-length copies	ratio solo-LTR/ full-length copy
Boty1	67	40	2
Boty2	8	16	0.5
Boty3	25	12	2
Boty4	40	10	4
Sclery1	972	24	40
Sclery2	78	9	9
Sclery3	526	18	29
Sclery4	633	7	90
