# Supplementary material for: Genomic Analysis of the Necrotrophic Fungal Pathogens Sclerotinia sclerotiorum and Botrytis cinerea
Source: PLoS Genet. 2011 Aug 18;7(8):e1002230. doi: 10.1371/journal.pgen.1002230 (PMC3158057; doi:10.1371/journal.pgen.1002230)
Supplement: Table S8 — Conservation of S. sclerotiorum and B. cinerea genes in OrthoMCL families. (PDF) [file pgen.1002230.s019.pdf]

Table S8

Conservation of *S. sclerotiorum* and *B. cinerea* genes in OrthoMCL families.*

	Families (#)			Genes (#)		
	S. sclerotiorum	*B. cinerea* B05.10	*B. cinerea* T4	*S. sclerotiorum*	*B. cinerea* B05.10	*B. cinerea* T4
All fungi, single copy	2,879	2,879	2,879	2,879	2,879	2,879
All fungi, with paralogs	527	527	527	631	703	669
Highly conserved (7-9 species)	2,877	2,881	2,857	3,242	3,200	3,161
Conserved (4-6 species)	968	1,200	1,215	1,036	1,244	1,253
Weakly conserved (1-3 species)	129	248	271	155	256	275
Specific to Leotiomycetes (*S. sclerotiorum*, *B. cinerea*, and *B. graminis*)	57	62	58	58	68	58
Specific to *S. sclerotiorum* and *B. cinerea*	1,562	1,451	1,433	1,600	1,474	1,454
Specific to *B. cinerea* (both genomes)	-	2,331	2,331	-	2,356	2,339
Strain specific, in families	80	34	11	329	80	32
Total in families				9,930	12,260	12,120
Not in families	-	-	-	4,592	4,188	4,240
**Total**	**9,079**	**11,613**	**11,582**	**14,522**	**16,448**	**16,360**

*Proteins from 10 genomes were clustered into families using OrthoMCL version 1.4.
