# Supplementary material for: Genomic Analysis of the Necrotrophic Fungal Pathogens Sclerotinia sclerotiorum and Botrytis cinerea
Source: PLoS Genet. 2011 Aug 18;7(8):e1002230. doi: 10.1371/journal.pgen.1002230 (PMC3158057; doi:10.1371/journal.pgen.1002230)
Supplement: Table S9 — GO Term enrichment for genes specific to S. sclerotiorum and B. cinerea. (PDF) [file pgen.1002230.s020.pdf]

Table S9

GO Term enrichment for genes specific to *S. sclerotiorum* and *B. cinerea**.

	GO Term	Name	FDR	single test p-Value	# in test group	# in reference group
Ss/Bc specific	GO:0046914	transition metal ion binding	2.90E-05	7.10E-08	84	774
Ss/Bc specific	GO:0008270	zinc ion binding	6.30E-05	3.40E-07	62	521
Ss/Bc specific	GO:0043167	ion binding	1.50E-04	1.50E-06	95	987
Ss/Bc specific	GO:0043169	cation binding	1.50E-04	1.50E-06	95	987
Ss/Bc specific	GO:0046872	metal ion binding	1.50E-04	2.00E-06	90	924
Ss/Bc specific	GO:0003700	transcription factor activity	5.50E-04	7.60E-06	26	155
Ss/Bc specific	GO:0004497	monooxygenase activity	1.40E-03	1.90E-05	24	144
Ss/Bc specific	GO:0016712	oxidoreductase activity, acting on paired donors, with incorporation or reduction of molecular oxygen, reduced flavin or flavoprotein as one donor, and incorporation of one atom of oxygen	3.10E-03	5.70E-05	5	4
Ss/Bc specific	GO:0046906	tetrapyrrole binding	3.70E-03	9.50E-05	21	130
Ss/Bc specific	GO:0020037	heme binding	3.70E-03	9.50E-05	21	130
Ss/Bc specific	GO:0016798	hydrolase activity, acting on glycosyl bonds	2.00E-02	5.40E-04	23	172
Ss/Bc specific	GO:0030528	transcription regulator activity	2.00E-02	5.50E-04	28	230
Ss/Bc specific	GO:0016705	oxidoreductase activity, acting on paired donors, with incorporation or reduction of molecular oxygen	3.60E-02	7.40E-04	9	36
Ss/Bc specific	GO:0004553	hydrolase activity, hydrolyzing O-glycosyl compounds	3.60E-02	1.10E-03	21	159
Ss/Bc specific	GO:0009055	electron carrier activity	3.70E-02	1.50E-03	18	130
Ss specific	GO:0046906	tetrapyrrole binding	9.60E-09	9.00E-12	31	120
Ss specific	GO:0020037	heme binding	9.60E-09	9.00E-12	31	120
Ss specific	GO:0004523	ribonuclease H activity	9.60E-09	1.20E-11	14	16
Ss specific	GO:0009055	electron carrier activity	9.60E-09	2.50E-11	29	119
Ss specific	GO:0003964	RNA-directed DNA polymerase activity	9.80E-09	7.90E-11	12	11
Ss specific	GO:0004497	monooxygenase activity	1.20E-08	1.30E-10	30	138
Ss specific	GO:0016891	endoribonuclease activity, producing 5'-phosphomonoesters	2.70E-08	4.30E-10	14	23

Ss specific	GO:0016893	endonuclease activity, active with either ribo- or deoxyribonucleic acids and producing 5'-phosphomonoesters	7.40E-08	2.10E-09	14	27
Ss specific	GO:0004521	endoribonuclease activity	9.40E-08	3.00E-09	14	28
Ss specific	GO:0005506	iron ion binding	4.40E-07	1.30E-08	33	205
Ss specific	GO:0034061	DNA polymerase activity	5.30E-07	1.60E-08	13	27
Ss specific	GO:0004540	ribonuclease activity	3.90E-06	1.40E-07	14	41
Ss specific	GO:0004519	endonuclease activity	7.20E-06	2.30E-07	14	43
Ss specific	GO:0004518	nuclease activity	5.20E-04	2.10E-05	15	77
Ss specific	GO:0016779	nucleotidyltransferase activity	4.60E-03	1.30E-04	13	71
Ss specific	GO:0016491	oxidoreductase activity	6.60E-03	2.20E-04	61	778
Ss specific	GO:0003677	DNA binding	1.90E-02	7.00E-04	39	452
Ss specific	GO:0046914	transition metal ion binding	1.90E-02	7.30E-04	60	798
Ss specific	GO:0003676	nucleic acid binding	3.10E-02	1.20E-03	63	866
Ss specific	GO:0046872	metal ion binding	3.60E-02	1.60E-03	67	947
Ss specific	GO:0005488	binding	3.80E-02	1.80E-03	174	3020
B. cinerea specific	GO:0046914	transition metal ion binding	9.90E-04	2.60E-06	71	917
B. cinerea specific	GO:0016491	oxidoreductase activity	1.10E-03	4.10E-06	72	947
B. cinerea specific	GO:0004497	monooxygenase activity	1.40E-03	8.10E-06	24	186
B. cinerea specific	GO:0046906	tetrapyrrole binding	2.20E-03	2.00E-05	21	158
B. cinerea specific	GO:0020037	heme binding	2.20E-03	2.00E-05	21	158
B. cinerea specific	GO:0009055	electron carrier activity	4.70E-03	5.10E-05	20	156
B. cinerea specific	GO:0008270	zinc ion binding	1.40E-02	1.70E-04	48	622
B. cinerea specific	GO:0046872	metal ion binding	1.70E-02	2.60E-04	73	1098
B. cinerea specific	GO:0043167	ion binding	3.40E-02	5.80E-04	75	1168
B. cinerea specific	GO:0043169	cation binding	3.40E-02	5.80E-04	75	1168
Bc (B05.10) specific	GO:0003682	chromatin binding	7.00E-04	6.20E-06	8	12
Bc (B05.10) specific	GO:0003824	catalytic activity	2.70E-02	3.80E-04	239	3483
Bc (B05.10) specific	GO:0003964	RNA-directed DNA polymerase activity	3.40E-02	5.60E-04	4	4
Bc (B05.10) specific	GO:0008519	ammonium transmembrane transporter activity	4.20E-02	6.70E-04	3	1
Bc (B05.10) specific	GO:0015101	organic cation transmembrane transporter activity	4.20E-02	6.70E-04	3	1

*Enrichment analysis measured using Fisher's exact test as implemented by the Gossip package in Blast2GO. Test and reference set total GO annotated proteins as follows: Ss/Bc specific (324 test, 5432 reference); Ss specific (271 test, 5485 reference); *B. cinerea* specific (274 test, 6163 reference); Bc (B05.10 specific) (360 test, 6077 reference). Enriched terms in the molecular function ontology are listed.
