# Supplementary material for: Genomic Analysis of the Necrotrophic Fungal Pathogens Sclerotinia sclerotiorum and Botrytis cinerea
Source: PLoS Genet. 2011 Aug 18;7(8):e1002230. doi: 10.1371/journal.pgen.1002230 (PMC3158057; doi:10.1371/journal.pgen.1002230)
Supplement: Table S10 — PFAM domain enrichment in S. sclerotiorum and B. cinerea. (PDF) [file pgen.1002230.s021.pdf]

Table S10

PFAM domain enrichment in *S. sclerotiorum* and *B. cinerea*.

Species are abbreviated as: Ss: *S. sclerotiorum*; Bc: *B. cinerea* (strain B05.10 or T4); Bg : *B. graminis*; Pn: *P. nodorum*.; Pt : *P. teres f. teres*; Gz: *G. zeae*; Mo: *M. oryzae*; Nc: *N. crassa*; An: *A. niger*. Plant pathogenic species are marked in grey shading, with necrotrophs in dark grey, and (hemi)-biotrophs in light grey.

domain	description	Ss	BcB05.10	BcT4	Bg	Pn	Pt	Gz	Mo	Nc	An	**Total**	pval	qval
PF03659.8	Glycosyl hydrolase family 71	10	9	8	0	0	1	2	0	0	1	**31**	4.30E-09	1.98E-06
PF00295.11	Glycosyl hydrolases family 28	17	19	13	0	3	4	2	3	6	8	**75**	1.46E-07	5.98E-05
PF11807.2	Domain of unknown function (DUF3328)	20	26	23	2	8	12	5	13	5	5	**119**	4.92E-07	0.000181
PF12224.2	Putative amidoligase enzyme	8	8	6	0	0	1	1	3	4	0	**31**	6.33E-05	0.020907
PF00651.25	BTB/POZ domain	18	31	22	5	12	18	5	10	14	2	**137**	6.80E-05	0.020907
PF09260.5	Domain of unknown function (DUF1966)	5	5	5	0	0	0	0	1	0	3	**19**	0.00014	0.039656
PF00732.13	GMC oxidoreductase	26	34	31	1	12	24	6	16	21	16	**187**	0.000152	0.040025
PF10250.3	GDP-fucose protein O-fucosyltransferase	4	5	3	2	0	0	0	0	0	0	**14**	0.000166	0.040904
