# Supplementary material for: Genomic Analysis of the Necrotrophic Fungal Pathogens Sclerotinia sclerotiorum and Botrytis cinerea
Source: PLoS Genet. 2011 Aug 18;7(8):e1002230. doi: 10.1371/journal.pgen.1002230 (PMC3158057; doi:10.1371/journal.pgen.1002230)
Supplement: Table S11 — PFAM domain depletion in S. sclerotiorum and B. cinerea. (PDF) [file pgen.1002230.s022.pdf]

## Table S11

**PFAM domain depletion in *S. sclerotiorum* and *B. cinerea*. ***

Species are abbreviated as: Ss: *S. sclerotiorum*; Bc: *B. cinerea* (strain B05.10 or T4); Bg : *B. graminis*; Pn: *P. nodorum*.; Pt : *P. teres f. teres*; Gz: *G. zeae*; Mo: *M. oryzae*; Nc: *N. crassa*; An: *A. niger*. Plant pathogenic species are marked in grey shading, with necrotrophs in dark grey, and (hemi)-biotrophs in light grey.

| domain | description | Ss | BcB05.10 | BcT4 | Bg | Pn | Pt | Gz | Mo | Nc | An | **Total** | pval | qval |
|---|---|---|---|---|---|---|---|---|---|---|---|---|---|---|
| PF00035.19 | Double-stranded RNA binding | 0 | 0 | 0 | 1 | 3 | 1 | 1 | 1 | 2 | 1 | **10** | 7 | 0 |
| PF08574.4 | Domain of unknown function DUF1762 | 0 | 0 | 0 | 1 | 1 | 1 | 2 | 1 | 1 | 1 | **8** | 7 | 0 |
| PF11374.2 | Domain of unknown function DUF3176 | 0 | 0 | 0 | 0 | 2 | 4 | 3 | 5 | 6 | 3 | **23** | 6 | 0 |
| PF11785.2 | Aft1 osmotic stress response | 0 | 0 | 0 | 1 | 0 | 1 | 1 | 1 | 1 | 1 | **6** | 6 | 0 |
| PF01436.15 | NHL repeat | 0 | 0 | 0 | 0 | 1 | 1 | 1 | 0 | 1 | 1 | **5** | 5 | 0 |
| PF02417.9 | Chromate transporter | 0 | 0 | 0 | 0 | 0 | 1 | 1 | 1 | 1 | 1 | **5** | 5 | 0 |
| PF02615.8 | Malate/L-lactate dehydrogenase | 0 | 0 | 0 | 0 | 1 | 1 | 0 | 1 | 1 | 1 | **5** | 5 | 0 |
| PF03088.10 | Strictosidine synthase | 0 | 0 | 0 | 0 | 1 | 1 | 1 | 1 | 1 | 0 | **5** | 5 | 0 |
| PF04182.6 | B-block binding subunit of TFIIIC | 0 | 0 | 0 | 0 | 1 | 0 | 1 | 1 | 1 | 1 | **5** | 5 | 0 |
| PF04303.7 | PrpF protein | 0 | 0 | 0 | 0 | 4 | 2 | 1 | 0 | 2 | 1 | **10** | 5 | 0 |
| PF04927.6 | Seed maturation protein | 0 | 0 | 0 | 0 | 1 | 1 | 0 | 1 | 1 | 1 | **5** | 5 | 0 |
| PF05406.9 | WGR domain | 0 | 0 | 0 | 0 | 1 | 1 | 1 | 0 | 1 | 1 | **5** | 5 | 0 |
| PF05572.7 | Pregnancy-associated plasma protein-A Merops family M43 | 0 | 0 | 0 | 0 | 0 | 3 | 2 | 1 | 2 | 1 | **9** | 5 | 0 |
| PF06632.6 | DNA double-strand break repair and V(D)J recombination protein XRCC4 | 0 | 0 | 0 | 0 | 1 | 1 | 1 | 1 | 0 | 1 | **5** | 5 | 0 |
| PF07335.5 | Fungal chitosanase | 0 | 0 | 0 | 0 | 1 | 1 | 1 | 1 | 1 | 0 | **5** | 5 | 0 |

| | | | | | | | | | | | | | | |
|---|---|---|---|---|---|---|---|---|---|---|---|---|---|---|
| PF09056.5 | Prokaryotic phospholipase A2 | 0 | 0 | 0 | 0 | 0 | 1 | 2 | 1 | 1 | 1 | **6** | 5 | 0 |
| PF09347.4 | Domain of unknown function DUF1989 | 0 | 0 | 0 | 0 | 1 | 2 | 1 | 0 | 2 | 2 | **8** | 5 | 0 |
| PF09724.3 | Domain of unknown function DUF2036 | 0 | 0 | 0 | 1 | 1 | 1 | 2 | 0 | 1 | 0 | **6** | 5 | 0 |
| PF10306.3 | Hypothetical protein FLILHELTA | 0 | 0 | 0 | 0 | 0 | 1 | 1 | 1 | 1 | 1 | **5** | 5 | 0 |
| PF10346.3 | Conidiation protein 6 | 0 | 0 | 0 | 0 | 0 | 1 | 1 | 1 | 1 | 1 | **5** | 5 | 0 |
| PF10496.3 | SNARE-complex protein Syntaxin-18 N-terminus | 0 | 0 | 0 | 1 | 1 | 1 | 1 | 0 | 0 | 1 | **5** | 5 | 0 |
| PF01048.14 | Phosphorylase superfamily | 1 | 0 | 1 | 2 | 14 | 10 | 9 | 3 | 1 | 2 | **43** | 9 | 1.56E-07 |
| PF00082.16 | Subtilase family | 8 | 7 | 4 | 5 | 8 | 30 | 26 | 7 | 11 | 11 | **117** | 10 | 1.05E-06 |
| PF02225.16 | PA domain | 2 | 2 | 1 | 1 | 0 | 11 | 12 | 4 | 6 | 6 | **45** | 9 | 4.32E-05 |
| PF04082.12 | Fungal specific transcription factor domain | 69 | 80 | 81 | 16 | 129 | 187 | 74 | 46 | 52 | 52 | **786** | 10 | 7.88E-05 |
| PF00703.15 | Glyco_hydro_2 | 1 | 2 | 1 | 0 | 1 | 10 | 6 | 4 | 7 | 5 | **37** | 9 | 0.000134 |
| PF05922.10 | Peptidase inhibitor I9 | 2 | 2 | 0 | 2 | 1 | 11 | 6 | 5 | 3 | 4 | **36** | 9 | 0.000192 |
| PF02837.12 | Glycosyl hydrolases family 2, sugar binding | 2 | 2 | 1 | 0 | 1 | 10 | 6 | 4 | 7 | 5 | **38** | 9 | 0.000481 |
| PF06280.6 | Fn3-like domain (DUF1034) | 1 | 1 | 0 | 0 | 0 | 2 | 12 | 1 | 1 | 3 | **21** | 7 | 0.001246 |
| PF04616.8 | Glycosyl hydrolases family 43 | 5 | 6 | 4 | 0 | 4 | 17 | 16 | 3 | 10 | 8 | **73** | 9 | 0.001776 |
| PF00172.12 | Fungal Zn(2)-Cys(6) binuclear cluster domain | 70 | 85 | 114 | 20 | 117 | 204 | 109 | 58 | 36 | 53 | **866** | 10 | 0.002387 |
| PF12874.1 | Zinc-finger of C2H2 type | 4 | 4 | 1 | 3 | 4 | 7 | 5 | 8 | 8 | 5 | **49** | 10 | 0.00258 |
| PF02655.8 | ATP-grasp domain | 0 | 0 | 1 | 1 | 1 | 2 | 3 | 3 | 1 | 1 | **13** | 8 | 0.003323 |
| PF00001.15 | 7 transmembrane receptor (rhodopsin family) | 0 | 1 | 0 | 1 | 1 | 5 | 4 | 1 | 0 | 0 | **13** | 6 | 0.003323 |
| PF03936.10 | Terpene synthase family, metal binding domain | 0 | 2 | 1 | 0 | 5 | 6 | 8 | 0 | 0 | 1 | **23** | 6 | 0.003729 |

* Domains listed which are found in at least 5 of the searched species.
