# Supplementary material for: Genomic Analysis of the Necrotrophic Fungal Pathogens Sclerotinia sclerotiorum and Botrytis cinerea
Source: PLoS Genet. 2011 Aug 18;7(8):e1002230. doi: 10.1371/journal.pgen.1002230 (PMC3158057; doi:10.1371/journal.pgen.1002230)
Supplement: Table S12 — Signaling pathway components in S. sclerotiorum and B. cinerea as compared to orthologs in three filamentous fungi and S. cerevisiae. (PDF) [file pgen.1002230.s023.pdf]

Table S12

Signaling pathway components in *S. sclerotiorum* and *B. cinerea* as compared to orthologs in three filamentous fungi and *S. cerevisiae*.

Function	*S. sclerotiorum*	*B. cinerea* B05.10	*B. cinerea* T4	Given name	*A. nidulans*	*N. crassa*	*M. oryzae*	*S. cerevisiae*
**G protein-coupled receptors (GPCRs)**								
STE2 (pheromone receptor)	SS1G_10310.1	BC1G_13582.1	BofuT4_P092750.1		AN2520 (GprA)	NCU05758 (PRE-2)	MGG_04711	Ste2p
STE3 (pheromone receptor)	SS1G_07464.1	BC1G_07387.1	BofuT4_P103670/80.1		AN7743 (GprB)	NCU00138 (PRE-1)	MGG_06452	Ste3p
Git3, Git3_C	SS1G_08243.1	BC1G_03450.1	BofuT4_P133070.1	BcGPR1[B]	-	NCU06312 (GPR-4)	MGG_08803	Gpr1p
Git3	SS1G_07511.1	BC1G_05052.1	BofuT4_P129750.1		AN3765 (GprC)	-	-	-
GPCR, secretin-like	SS1G_09369.1	BC1G_02359.1	BofuT4_P141110.1		-	NCU00786 (GPR-1)	-	-
GPCR, secretin-like	SS1G_14220.1	BC1G_02195.1	BofuT4_P142730.1		AN6680	NCU03253	-	-
GPCR, family 2-like	SS1G_08113.1	BC1G_04448.1	BofuT4_P143710.1		AN8262 (GprH)	NCU04626 (GPR-2)	-	-
GPCR, family 2-like	SS1G_11756.1	BC1G_06905.1	BofuT4_P088050.1		-	NCU09427 (GPR-3)	-	-
PQ-loop	SS1G_07709.1	BC1G_02874.1	BofuT4_P004540.1		-	NCU00300 (GPR-5)	MGG_04698	Rtc2p
PQ-loop	SS1G_03605.1	BC1G_08371.1	BofuT4_P010450.1		AN5720 (GprJ)	NCU09195 (GPR-6)	MGG_02855	-
PQ-loop	SS1G_04557.1	BC1G_08288.1	BofuT4_P014060.1		AN7766	NCU02168	-	-
PQ-loop	SS1G_01814.1	BC1G_02579.1	BofuT4_P108920.1		AN8217	NCU09543	MGG_08175	-
Microbial opsin	SS1G_01614.1	BC1G_02456.1	BofuT4_P110210.1	BOP1[B]	AN3361 (NopA)	NCU10055 (NOP-1)	-	-
Microbial opsin	SS1G_04339.1	BC1G_13906.1	BofuT4_P163470.1	BOP2	-	NCU01735 (ORP-1)	MGG_09015	Mrh1p
**Heterotrimeric G protein subunits and regulatory proteins**								
Gα subunit	SS1G_12343.1	BC1G_01681.1	BofuT4_P134310.1	BCG1[B]	AN0651 (FadA)	NCU06493 (GNA-1)	MGG_00365 (MAGB)	--
Gα subunit	SS1G_10286.1	BC1G_08985.1	BofuT4_P059220.1	BCG2[B]	AN3090 (GanA)	NCU06729 (GNA-2)	MGG_04204 (MAGC)	Gpa1p
Gα subunit	SS1G_07597.1	BC1G_03006.1	BofuT4_P003150.1	BCG3[B]	AN1016 (GanB)	NCU05206 (GNA-3)	MGG_01818	Gpa2p
Gβ subunit	SS1G_03482.1	*supercontig 1.46* [C]	BofuT4_P043580.1	BcGB1[B]	AN0081 (SfaD)	NCU00440 (GNB-1)	MGB1	Ste4p
Gβ-like protein	SS1G_00900.1	BC1G_10054.1	BofuT4_P057460.1	BcGBL1	AN4163 (CpcB)	NCU05810 (CPC-2)	MGG_04719	Cpc2p
Gγ subunit	SS1G_12567.1	BC1G_15399.1	BofuT4_P096910.1	BcGG1[B]	AN2742 (GpgA)	NCU00041 (GNG-1)	MGG_10193	Ste18p
Regulator of G protein (RGS)	SS1G_09971.1	BC1G_07268.1	BofuT4_P138310.1		AN5893 (FlbA)	NCU08319	MGG_14517	Sst2p
Regulator of G protein (RGS)	SS1G_12176.1	BC1G_01765.1	BofuT4_P135240.1		AN3622 (RgsB)	NCU08343	MGG_03726	Rax1p
Regulator of G protein (RGS)	SS1G_03881.1	BC1G_04470.1	BofuT4_P143950/60.1		AN5755 (RgsA)	NCU05435	MGG_03146	-
Regulator of G protein (RGS)	SS1G_14426.1	BC1G_09636/7.1	BofuT4_P026060/70.1		AN1377 (RgsC)	NCU03937	MGG_00990	Mdm1p
Regulator of G protein (RGS)	SS1G_01379.1	BC1G_08584.1	BofuT4_P122700.1		-	NCU07268	MGG_08735	Rgs2p
Regulator of G protein (RGS)	SS1G_10234.1	BC1G_08485.1	BofuT4_P058760.1		-	NCU03153	MGG_03209	-
Regulator of G protein (RGS)	SS1G_05421.1	BC1G_09930.1	BofuT4_P055930.1		-	-	MGG_02753	-
Phosducin	SS1G_03480.1	BC1G_12682.1	BofuT4_P009640.1		AN0082 (PhnA)	NCU00441	MGG_12918	-
Phosducin	SS1G_09422.1	BC1G_05790.1	BofuT4_P110440.1		AN4561 (PhnB)	NCU00617	MGG_12576	Plp2p
Phosducin	SS1G_00429.1	BC1G_00680.1	BofuT4_P020410.1		AN8847 (PhnC)	-	-	Plp1p

**Components of the cAMP cascade**								
Adenylate cyclase (AC)	SS1G_07715.1	BC1G_02865.1	BofuT4_P004620.1	BAC[B]	AN3913 (CyaA)	NCU08377 (CR-1)	MGG_09898 (MAC1)	Cyr1p
AC-associated protein	SS1G_13327.1	BC1G_14507.1	BofuT4_P077100.1		AN0999 (CapA)	NCU08008	MGG_01722	Srv2p
PKA regulatory subunit	SS1G_10536.1	BC1G_10590.1	BofuT4_P115900.1	BcPKAR[B]	AN4987 (PkaR)	NCU01166 (MCB)	MGG_07335 (SUM1)	Bcy1p
PKA catalytic subunit	SS1G_13577.1	BC1G_10410.1	BofuT4_P083080.1	BcPKA1[B]	AN6305 (PkaA)	NCU06240 (PKAC-1)	MGG_06368 (CPKA)	Tpk2p
PKA catalytic subunit	SS1G_03171.1	BC1G_07542.1	BofuT4_P084160.1	BcPKA2[B]	AN4717 (PkaB)	NCU0068	MGG_02832	Tpk3p
cAMP phosphodiesterase	SS1G_07412.1	BC1G_07334.1	BofuT4_P071720.1	BcPDE1[B]	AN0829 (PdeA)	NCU00237	MGG_07707	Pde1p
cAMP phosphodiesterase	SS1G_12594.1	BC1G_08417.1	BofuT4_P010960.1	BcPDE2[B]	AN2740 (PdeB)	NCU00478	MGG_05664	Pde2p
cNMP-binding protein	SS1G_13049.1	BC1G_14332.1	BofuT4_P154620.1		AN2481	NCU08276	MGG_06143	Nte1p
cNMP-binding protein	SS1G_05838.1	BC1G_01623.1	BofuT4_P043070.1		AN4510	NCU08642	MGG_09408	-
Sch9-like protein kinase	SS1G_01124.1	BC1G_14873.1	BofuT4_P090340.1	BPK2[B]	AN4238 (SchA)	NCU03200 (SCK-1)	MGG_01251	Sch9p
CLK1-like protein kinase	SS1G_11231.1	BC1G_05432.1	BofuT4_P073120.1	BPK3[B]	AN1632	NCU00188 (ATG-1)	MGG_06393 (ATG1)	Atg1p
Ran1-like protein kinase	SS1G_06542.1	BC1G_04819.1	BofuT4_P149160.1	BPK4[B]	AN4935	NCU04990	MGG_05074	Sks1p
Snf1-like protein kinase	SS1G_10426.1	BC1G_12009.1	BofuT4_P032520.1	BcSNF1[B]	AN7695	NCU04566	MGG_00803	Snf1p
**Monomeric GTPase modules (Ras and Rho family)**								
Ras-like GTPase	SS1G_13801.1	BC1G_10176.1	*bt4exctg_0063* [C]	BcRAS1[B]	AN0182	NCU08823 (RAS-1)	MGG_06154	Ras2p
Ras-like GTPase	SS1G_03860.1	BC1G_04437.1	BofuT4_P143580.1	BcRAS2[B]	AN5832 (RasB)	NCU03616 (RAS-2)	MGG_09499	-
Ras-like GTPase	SS1G_03294.1	*19866915874266* [C]	BofuT4_P131410/20.1		AN4685	NCU02167 (KREV-1)	-	Rsr1p
RasGAP protein	SS1G_12053.1	BC1G_13130.1	BofuT4_P103170.1		AN9463	NCU03116	MGG_03700	Iqg1p
RasGAP protein	SS1G_13541.1	BC1G_13387.1	BofuT4_P079010.1		-	NCU01642	MGG_11425	Ira1/2p
RasGAP protein	SS1G_11299.1	BC1G_05354.1	BofuT4_P073910.1		AN4998	NCU06122	MGG_03846	-
RasGAP protein	SS1G_04989.1	BC1G_13602.1	BofuT4_P159270.1		AN3735	NCU03852	MGG_08105	Bud2p
RasGEF protein	SS1G_12228.1	BC1G_01830/1.1	BofuT4_P135860.1		AN3092	NCU03379	MGG_02419	Lte1p
RasGEF protein	SS1G_03113.1	BC1G_07606.1	BofuT4_P084890.1		AN2130	NCU06500	MGG_00371	Cdc25p
RasGEF protein	SS1G_10918.1	BC1G_03942.1	BofuT4_P009000.1		AN4369	NCU09758	MGG_00199	-
RasGEF protein	SS1G_10502.1	BC1G_15362.1	BofuT4_P098600.1		AN0449	NCU01782	MGG_11784	-
Rac-like GTPase	SS1G_13183.1	-	BofuT4_P147150.1	BcRAC[B]	AN4743 (RacA)	NCU02160 (RAC-1)	MGG_02731 (RAC1)	-
Cdc42-like GTPase	SS1G_13726.1	BC1G_15519.1	*bt4exctg_0388* [C]	BcCDC42[B]	AN7487 (Cdc42)	NCU06454 (CDC-42)	MGG_00466	Cdc42p
Rho-like GTPase	SS1G_03867.1	BC1G_04486.1	BofuT4_P144140.1		AN5740	NCU01484 (RHO-1)	MGG_07176	Rho1p
Rho-like GTPase	SS1G_06571.1	BC1G_04850.1	BofuT4_P149460.1		AN4953	NCU08683 (RHO-2)	MGG_02457	Rho2p
Rho-like GTPase	SS1G_02769.1	BC1G_02425.1	BofuT4_P140420.1		AN4782	NCU00600 (RHO-3)	MGG_10323	Rho3p
Rho-like GTPase	SS1G_02182.1	BC1G_04274.1	BofuT4_P125500.1		AN2687	NCU03407 (RHO-4)	MGG_03901	Rho4p
RhoGEF protein	SS1G_03178.1	BC1G_07524.1	BofuT4_P083980/90.1		AN4719	NCU00668	MGG_03064	Rom1/2p
RhoGEF protein	SS1G_05721.1	BC1G_09394.1	BofuT4_P148340.1	BcCDC24	AN5592	NCU06067	MGG_09697	Cdc24p
RhoGEF protein	SS1G_00697.1	BC1G_00562/3.1	BofuT4_P021580.1		AN7783	NCU02131	MGG_12644	Tus1p
RhoGEF protein	SS1G_13780/1.1	BC1G_10162/3.1	*bt4exctg_0066* [C]		AN0113	NCU06579	MGG_01695	Bud3p
RhoGEF protein	SS1G_04527.1	BC1G_00381.1	BofuT4_P046280.1		AN5481	NCU02764	MGG_09954	-
RhoGEF protein	SS1G_04044.1	BC1G_00322.1	BofuT4_P046910.1		AN3754	NCU10282	MGG_11178	-

RhoGAP protein	SS1G_03088.1	BC1G_06319.1	BofuT4_P070310.1		AN5677	NCU09537	MGG_09531	Rgd2p
RhoGAP protein	SS1G_07736.1	BC1G_13633.1	BofuT4_P006110.1		AN7576	NCU02689	MGG_04377	Lrg1p
RhoGAP protein	SS1G_01323.1	BC1G_09755.1	BofuT4_P116760.1		AN1025	NCU07688	MGG_04186	Rga1p
RhoGAP protein	SS1G_04496.1	BC1G_00408.1	BofuT4_P046000.1		AN5787	NCU02524	MGG_09275	Bem3p
RhoGAP protein	SS1G_03990.1	BC1G_15133.1	BofuT4_P160190.1		AN4745	NCU00553	MGG_03048	Rgd1p
RhoGAP protein	SS1G_12350.1	BC1G_01690.1	BofuT4_P134380.1		AN7650	NCU00196	MGG_06390	Sac7p
RhoGAP protein	SS1G_03234.1	BC1G_06793.1	BofuT4_P132020.1		AN6850	NCU01472	MGG_04006	Rga2p
RhoGAP protein	SS1G_12768.1	BC1G_05255.1	BofuT4_P069410.1		AN3746	NCU10647	MGG_09303	Bem3p
RhoGDI protein	SS1G_08512.1	BC1G_08670.1	BofuT4_P001160.1		AN0163	NCU06561	MGG_01689	Rdi1p
**Histidine kinases and related proteins (two-component signal transduction)** [A]								
Histidine kinase - group I	SS1G_11201.1	BC1G_09793.1	BofuT4_P116260.1	4MPY[A]	AN6820	-	-	-
Histidine kinase - group I	SS1G_10091.1	BC1G_12549.1	BofuT4_P012000.1	M3M1[A]	AN4818	NCU09520	-	-
Histidine kinase - group I	SS1G_12461.1	BC1G_03653.1	BofuT4_P022820.1	M3EW[A]	AN4113	-	MGG_12530	-
Histidine kinase - group I	SS1G_09016.1	BC1G_10680.1	BofuT4_P152210/20.1	M3R8[A]	AN2363	-	-	-
Histidine kinase - group I	SS1G_01288.1	BC1G_12076.1	BofuT4_P090970.1	M4GK[A]	AN9048	-	-	-
Histidine kinase - group III	SS1G_12694.1	BC1G_00374.1	BofuT4_P046300.1	BOS1[A,B]	AN4479 (NikA)	NCU02815 (NIK-1)	MGG_11174 (HIK1)	-
Histidine kinase - group V	SS1G_05695.1	BC1G_09381.1	BofuT4_P148190.1	HHK2[A]	AN3101	NCU01833 (CHK-1)	MGG_11882	-
Histidine kinase - group VI	SS1G_10260.1	BC1G_08461.1	BofuT4_P059010.1	HHK5[A,B]	AN1800	NCU04615	MGG_07312	Sln1p
Histidine kinase - group VII	SS1G_04489.1	BC1G_00418.1	BofuT4_P045870.1	HHK17[A]	AN4447	-	-	-
Histidine kinase - group VIII	SS1G_13512.1	BC1G_13369.1	BofuT4_P078780.1	PHY1[A]	AN9008 (FphA)	NCU04834 (PHY-1)	MGG_12377	-
Histidine kinase - group VIII	SS1G_12828.1	BC1G_08283.1	BofuT4_P014010.1	PHY2[A]	-	-	-	-
Histidine kinase - group VIII	SS1G_02650.1	BC1G_01106.1	BofuT4_P030530.1	PHY3[A]	-	-	-	-
Histidine kinase - group IX	SS1G_06305	BC1G_15679/80.1	*bt4exctg_0601* [C]	HHK6[A]	-	NCU02057	MGG_01342	-
Histidine kinase - group IX	SS1G_07900.1	BC1G_07772.1	BofuT4_P006790.1	M4WV[A]	-	-	-	-
Histidine kinase - group X	SS1G_05712.1	BC1G_09356/7.1	BofuT4_P147920.1	HHK1[A,B]	AN3102	NCU01823	MGG_06696	-
Histidine kinase - group XI	SS1G_13101.1	BC1G_15817.1	BofuT4_P085840/50.1	M3PY[A]	-	-	MGG_02665	-
Histidine kinase - group XI	SS1G_06181.1	BC1G_13015.1	BofuT4_P137390.1	M4R8[A]	AN7945	NCU00939	MGG_01227	-
Histidine kinase - group XI	SS1G_07801.1	BC1G_11971.1	BofuT4_P005300.1	M2QJp[A]	-	NCU03164	MGG_13891	-
Histidine kinase - group XI	SS1G_08636.1	BC1G_16263.1	BofuT4_P001590.1	M40J[A]	-	-	-	-
Histidine kinase - group XI	SS1G_08302.1	BC1G_11490.1	BofuT4_P133790.1	M3C8[A]	-	-	-	-
Response regulator	SS1G_03429.1	BC1G_07872.1	BofuT4_P044020.1	BRRG1[A,B]	AN7697 (SskA)	NCU01895 (RRG-1)	MGG_02897	Ssk1p
Response regulator	SS1G_08085.1	BC1G_12394.1	BofuT4_P024120.1	RIM15[A]	AN7572	NCU07378	MGG_00345	Rim15p
Response regulator	SS1G_11812.1	BC1G_06857.1	BofuT4_P088540.1	BRRG2[A]	AN3688 (SrrA)	NCU02413 (RRG-2)	MGG_03516	Skn7p
His-phosphotransfer (HPt)	SS1G_10394.1	BC1G_12045.1	BofuT4_P032950.1		AN2005	NCU01489	MGG_07173	Ypd1p
**Mitogen-activated protein kinase (MAPK) modules**								
p21-activated kinase (PAK)	SS1G_02450.1	BC1G_05000.1	BofuT4_P038920.1	BcSTE20	AN2067 (Ste20)	NCU03894	MST20	Ste20p
p21-activated kinase (PAK)	SS1G_00637.1	BC1G_06517.1	BofuT4_P051820.1	BcCLA4	AN8836	NCU00406	MGG_06320 (CHM1)	Cla4p
MAPK	SS1G_11866.1	BC1G_13966.1	BofuT4_P067340.1	BMP1[B]	AN3719 (MpkB)	NCU02393 (MAK-2)	MGG_09565 (PMK1)	Fus3p

MAPKK	SS1G_10792.1	BC1G_03809.1	BofuT4_P007690.1	BcSTE7[B]	AN3422	NCU04612	MGG_00800	Ste7p
MAPKKK	SS1G_00606.1	BC1G_06557.1	BofuT4_P052210.1	BcSTE11[B]	AN2269 (SteC)	NCU06182 (NRC-1)	MGG_12855	Ste11p
Scaffold protein	SS1G_03194.1	BC1G_07505.1	BofuT4_P083760.1	BcSTE50[B]	AN7252	NCU00455	MGG_05199	Ste50p
MAPK	SS1G_07590.1	BC1G_03001.1	BofuT4_P003200.1	BcSAK1[B]	AN1017 (HogA)	NCU07024 (OS-2)	MGG_01822 (OSM1)	Hog1p
MAPKK	SS1G_14143.1	BC1G_07633.1	BofuT4_P025670.1	BOS5[B]	AN0931 (PbsA)	NCU00587	MGG_10268	Pbs2p
MAPKKK	SS1G_06598.1	BC1G_04606.1	BofuT4_P080980.1	BOS4	AN1180	NCU03071 (OS-4)	MGG_00183	Ssk2p
MAPK	SS1G_05445.1	BC1G_07144.1	BofuT4_P053550.1	BMP3[B]	AN5666 (MpkA)	NCU11376 (MKC-1)	MGG_04943 (MPS1)	Slt2p
MAPKK	SS1G_00059.1	BC1G_11713.1	BofuT4_P016030.1	BcMKK1	AN4189	NCU06419	MGG_06482 (MKK1)	Mkk1p
MAPKKK	SS1G_10983.1	BC1G_11345.1	BofuT4_P153830.1	BcBCK1	AN4887	NCU02234	MGG_00883	Bck1p
**Ca2+-mediated signalling (including Ca2+-binding proteins, Ca2+ -permeable channels, Ca2+ transporters and cation pumps)**								
Phospholipase C	SS1G_05754.1	BC1G_01559.1	BofuT4_P042430.1	BcPLC1[B]	AN0664	NCU01266 (PLC)	MGG_02444 (PLC1)	Plc1p
Phospholipase C	SS1G_03548.1	BC1G_15592.1	BofuT4_P099650.1	BcPLC2	AN2947	NCU02175 (PLC-1)	MGG_02682	-
Protein kinase C	SS1G_14026.1	BC1G_07478.1	BofuT4_P001810.1	BcPKC1	AN0106	NCU06544	MGG_08689 (MPKC)	Pkc1p
Calmodulin (CaM)	SS1G_09670.1	BC1G_09287.1	BofuT4_P158310.1		AN2047	NCU04120 (CMD-1)	MGG_06884 (CaM)	Cmd1p
Calmodulin (CaM)	SS1G_01240.1	BC1G_03973.1	BofuT4_P035780.1		-	-	-	-
Calmodulin-like (plant)	SS1G_05131.1	BC1G_11227.1	BofuT4_P159960.1		-	-	-	-
Calcineurin A (CNA)	SS1G_01788.1	BC1G_02606.1	BofuT4_P108660/70..1	BcCNA[B]	AN8820	NCU03804 (CNA-1)	MGG_07456	Cna1p
Calcineurin B (CNB)	SS1G_00214.1	BC1G_06074.1	BofuT4_P017800.1		AN6566	NCU03833 (CNB-1)	MGG_06933	Cnb1p
CN-regulated TF	SS1G_04676.1	BC1G_00093.1	BofuT4_P049220.1	BcCRZ1[B]	AN5726 (CrzA)	NCU07952	MGG_05133	Crz1p
Regulator of calcineurin	SS1G_05345.1	BC1G_07084.1	BofuT4_P054220.1	BcRCN1[B]	AN6249	NCU01504	MGG_03218	Rcn1p
Neuronal Ca2+ sensor (NCS)	SS1G_01921.1	BC1G_12603.1	BofuT4_P139690.1		AN5341	NCU04379	MGG_01550 (NCS1)	Frq1p
Mechanosensitive ion channel	SS1G_01437.1	BC1G_07960.1	BofuT4_P122110.1		AN7571	NCU04207	MGG_08304	--------
Myosin regulatory light chain	SS1G_13945.1	BC1G_12804.1	BofuT4_P103950.1		AN6732	NCU06617	MGG_09470	Mlc1p
NADH dehydrogenase	SS1G_08382.1	BC1G_08718.1	BofuT4_P000630.1		AN7500	NCU05225	MGG_04140	Nde2p
Actin cytoskeleton- protein	SS1G_01315.1	BC1G_12578.1	BofuT4_P139440.1		AN1023	NCU06347 (END-3)	MGG_06180	End3p
Protein of spindle pole body	SS1G_00342.1	BC1G_00791.1	BofuT4_P019370.1		AN5618	NCU09871	MGG_00458	Cdc31p
No function predicted	SS1G_07304.1	BC1G_04731/ 2.1	BofuT4_P128780.1		AN0267	NCU00171	MGG_12886	-
Mitochondrial carrier protein	SS1G_11574.1	BC1G_11026.1	BofuT4_P155100.1		AN8785	NCU01241	MGG_07066	Agc1p
Mitochondrial carrier protein	SS1G_02257.1	BC1G_04379/ 80.1	BofuT4_P126560.1		AN2173	NCU01564	MGG_01072	Sal1p
Penta-EF-hand protein	SS1G_02888.1	BC1G_05708.1	BofuT4_P111170.1		AN2986	NCU02738	MGG_04818	Pef1p
Zinc finger, ZZ type	SS1G_12974.1	BC1G_05842.1	BofuT4_P145930.1		AN5197	NCU02115	MGG_00654	-
Calpactin heavy chain	SS1G_06532.1	BC1G_04812.1	BofuT4_P149090.1		AN2427	NCU04421 (ANX-14)	MGG_06847	-
Calreticulin/ calnexin	SS1G_04685.1	BC1G_00082.1	BofuT4_P049370.1		AN3592	NCU09265 (CNX-1)	MGG_01607	Cne1p
Ca2+/CaM-dependent kinase	SS1G_10021.1	BC1G_07228.1	BofuT4_P137810.1	BcCMK1	AN2412 (CmkA)	CAMK-1	MGG_09912	Cmk2p
Ca2+/CaM-dependent kinase	SS1G_11871.1	BC1G_15259.1	BofuT4_P067170.1	BcCMK2	AN3065 (CmkB)	NCU02283	MGG_00925	Cmk1p
Ca2+/CaM-dependent kinase	SS1G_00591.1	BC1G_06577.1	BofuT4_P052420.1	BcCMK3	AN8827 (CmkC)	NCU06177	MGG_06421	-
Protein kinase	SS1G_06203.1	BC1G_13037.1	BofuT4_P137080.1		AN4483	NCU09212	MGG_08547	Clk1p
Protein kinase	SS1G_12333.1	BC1G_13227.1	BofuT4_P106040.1		AN0822	NCU00914	MGG_01196	Kin4p

Protein kinase	SS1G_04069.1	BC1G_00346.1	BofuT4_P046640.1		AN4279	NCU02814 (PRD-4)	MGG_01596	Dun1p
RasGAP protein	SS1G_12053.1	BC1G_13130.1	BofuT4_P103170.1		AN9463	NCU03116	MGG_03700	Iqg1p
Myosin	SS1G_00204.1	BC1G_06083.1	BofuT4_P017710.1		AN8862	NCU11354 (MYO-2)	MGG_06923	Myo2p
Myosin	SS1G_05662.1	BC1G_10821.1	BofuT4_P147690.1		AN1558	NCU02111 (MYO-5)	MGG_00748	Myo5p
Ubiquitin-protein ligase	SS1G_00365.1	BC1G_00767.1	BofuT4_P019590.1		AN3999	NCU04800	MGG_09504	Hul5p
Ca2+ channel protein	SS1G_00577.1	BC1G_06592.1	BofuT4_P052520.1	BcMID1[B]	AN8842	NCU06703	MGG_12128	Mid1p
Ca2+ channel protein	SS1G_01169.1	BC1G_12101/2/3	BofuT4_P090740.1	BcCCH1[B]	AN1168	NCU02762	MGG_05643	Cch1p
Ca2+ channel protein	SS1G_08141.1	BC1G_03504.1	BofuT4_P119960/70.1	BcYVC1	AN3155	NCU11361	MGG_09828	Yvc1p
Ca2+/H+ antiporter	SS1G_12337.1	BC1G_01673.1	BofuT4_P134210/20.1		AN7173	NCU00916	MGG_08710	-
Ca2+/H+ antiporter	SS1G_13308.1	BC1G_06733.1	BofuT4_P077390.1		-	-	-	-
Ca2+/H+ antiporter	SS1G_00328.1	BC1G_00803.1	BofuT4_P019260.1		AN0471	NCU07075 (CAX)	-	Vcx1p
Ca2+/H+ antiporter	SS1G_09474.1	BC1G_01285.1	BofuT4_P028830.1		AN5821	NCU06366	MGG_13224	-
Ca2+/H+ antiporter	SS1G_01642/ 3.1	BC1G_14764.1	*bt4exctg_0111* [C]		AN6986	NCU05360	MGG_01381	Vnx1p
Ca2+/H+ antiporter	SS1G_14108.1	BC1G_13713.1	*bt4exctg_0041* [C]		-	-	-	-
Ca2+/H+ antiporter	SS1G_09377.1	BC1G_02347.1	BofuT4_P141220.1		AN7510	NCU07711	MGG_04159	-
Ca2+/Na+ antiporter	SS1G_12741.1	BC1G_05221.1	BofuT4_P069690.1		AN4266	NCU02826	MGG_01638	Ecm27p
Ca2+/Na+ antiporter	SS1G_06268.1	BC1G_01723.1	BofuT4_P134730.1		-	NCU08490	-	-
Ca2+-translocating ATPase	SS1G_12923.1	BC1G_08297.1	BofuT4_P014170.1		AN5743	NCU03305 (NCA-1)	MGG_04550	-
Ca2+-translocating ATPase	SS1G_06668.1	BC1G_03385.1	BofuT4_P080150.1		AN1189	NCU04736 (NCA-2)	MGG_02487	Pmc1p
Ca2+-translocating ATPase	-	BC1G_11540.1	-		AN6642	-	MGG_10730	Ena6p
Ca2+-translocating ATPase	SS1G_06551.1	BC1G_04830.1	BofuT4_P149250.1		AN1628	NCU05046 (ENA-1)	MGG_02074	Ena5p
Ca2+-translocating ATPase	SS1G_09885.1	BC1G_02758.1	BofuT4_P118380.1		AN7464	NCU03292 (PMR-1)	MGG_11727	Pmr1p
**Serine/threonine-protein phosphatases (catalytic subunits only)**								
Protein phosphatase PP2A	SS1G_08489.1	BC1G_08696.1	BofuT4_P000880.1		AN6391	NCU06630	MGG_06099	Pph21p
Protein phosphatase PP2A	SS1G_08513.1	BC1G_14387.1	BofuT4_P001170.1		AN0164	NCU06563	MGG_01690	Ppg1p
Protein phosphatase PP2B	SS1G_01788.1	BC1G_02606.1	BofuT4_P108660/70.1	BcCNA[B]	AN8820	NCU03804 (CNA-1)	MGG_07456	Cna1p
Protein phosphatase 1	SS1G_12553.1	BC1G_15413.1	BofuT4_P096770.1		AN0410	NCU00043	MGG_10195	Glc7p
Protein phosphatase 5	SS1G_13212.1	BC1G_15048.1	BofuT4_P105220.1		AN10281	NCU01433	MGG_06941	Ppt1p
Protein phosphatase 6	SS1G_13474.1	BC1G_06465.1	BofuT4_P035040.1		AN0504	NCU03436	MGG_03911	Sit4p
Protein phosphatase X	SS1G_08420.1	BC1G_03106.1	BofuT4_P081490.1		AN0103	NCU08301	MGG_01528	Pph3p
Protein phosphatase PP-Z	SS1G_02547.1	BC1G_01244.1	BofuT4_P029220.1		AN3793	NCU07489	MGG_00149	Ppz1p
Protein phosphatase 2C	SS1G_00528.1	BC1G_01996.1	BofuT4_P061930.1		AN6892	NCU00434	MGG_05207	Ptc1p
Protein phosphatase 2C	SS1G_10886.1	BC1G_03897.1	BofuT4_P008590.1		AN5722	NCU01767	MGG_03154	Ptc5p
Protein phosphatase 2C	SS1G_08167.1	BC1G_16333.1	BofuT4_P084940.1		AN1358	NCU04600	MGG_01351	Ptc2p
Protein phosphatase 2C	SS1G_01633.1	BC1G_02433.1	*bt4exctg_0105* [C]		AN0914	NCU03495	MGG_03918	Ptc6p

[A] Histidine kinases in *B. cinerea* B05.10 were identified and grouped into 11 classes by Catlett et al. (2003).
[B] Genes whose functions have been characterized in *B. cinerea* by gene replacement approaches (reviewed by Tudzynski and Kokkelink, 2009)
[C] Genes without gene call (contig information is given).
