# Supplementary material for: Genomic Analysis of the Necrotrophic Fungal Pathogens Sclerotinia sclerotiorum and Botrytis cinerea
Source: PLoS Genet. 2011 Aug 18;7(8):e1002230. doi: 10.1371/journal.pgen.1002230 (PMC3158057; doi:10.1371/journal.pgen.1002230)
Supplement: Table S13 — S. sclerotiorum and B. cinerea homologs of apoptosis-associated genes found in other fungi. (PDF) [file pgen.1002230.s024.pdf]

Table S13

S. sclerotiorum and _B. cinerea_ homologs of apoptosis-associated genes found in other fungal species.

S. sclerotiorum	*B. cinerea* B05.10	*B. cinerea* T4	Similarity
SS1G_13569.1	BC1G_10418.1	BofuT4_P083010.1	Endonuclease G (EndoG/Nuc1)
SS1G_00860.1	BC1G_09030.1	BofuT4_P059720.1	Apoptosis inducing factor (AIF)
SS1G_07596.1	BC1G_14521.1	--	Inhibitor of apoptosis (IAP)
SS1G_07244.1	BC1G_05107.1	BofuT4_P130390.1	BAG1
SS1G_01944.1	BC1G_15876.1	BofuT4_P139910.1	Omi/Htr2A
SS1G_10992.1	BC1G_11354.1	BofuT4_P153750.1	Metacaspase 1
SS1G_00007.1	--	BofuT4_P015340.1	RAD9
SS1G_12522.1	--	BofuT4_P022120.1	TCTP (translationally Controlled Tumor Protein)
SS1G_03316.1	BC1G_09605.1	BofuT4_P131190.1	BI-1 (Bax Inhibitor 1)
SS1G_12766.1	BC1G_05253.1	BofuT4_P069430.1	Ygl129c (DAP3)
SS1G_05532.1	BC1G_07009.1	BofuT4_P085550.1	Apaf1 (putative)
SS1G_05994.1	BC1G_09407.1	BofuT4_P148490.1	Poly(ADP-ribose) polymerase
