# Supplementary material for: Genomic Analysis of the Necrotrophic Fungal Pathogens Sclerotinia sclerotiorum and Botrytis cinerea
Source: PLoS Genet. 2011 Aug 18;7(8):e1002230. doi: 10.1371/journal.pgen.1002230 (PMC3158057; doi:10.1371/journal.pgen.1002230)
Supplement: Table S14 — S. sclerotiorum and B. cinerea orthologs of Aspergillus nidulans genes involved in mating and fruiting body development. (PDF) [file pgen.1002230.s025.pdf]

Table S14

S. sclerotiorum and *B. cinerea* orthologs of *Aspergillus nidulans* genes involved in mating and fruiting body development.

S.sclerotiorum	*B. cinerea* B05.10	*B. cinerea T4*	*A. nidulans*	Function	Role
SS1G_01435.1	BC1G_07957.1	BofuT4_P122140.1	AN1539 (*csnD*)	Signalosome subunit 4, regulation of sexual development	Fruiting Body Development
SS1G_03348.1	BC1G_09577.1	BofuT4_P130860.1	AN2129 (*csnE*)	Signalosome subunit 5, regulation of sexual development	Fruiting Body Development
SS1G_01477.1	BC1G_05510.1	BofuT4_P106710.1	AN8211	*U. maydis rum1*, repressor of b mating type regulated genes	Fruiting Body Development
SS1G_05588.1	Not found	BofuT4_P114070.1	AN8676	*U. maydis* umc1, MADS-box TF, modulator of pheromone-inducible gene expression	Fruiting Body Development
SS1G_09861.1	BC1G_02738.1	BofuT4_P118690.1	AN7349 (*mutA*)	Mutanase, cell wall turnover during sexual development	Fruiting Body Development
SS1G_13426.1	BC1G_06397.1	BofuT4_P062810.1	AN0135	*P. anserina cro1*, regulator of myosin function; required for syncytial to cellular transition	Fruiting Body Development
SS1G_02971.1	BC1G_02217.1-BC1G_02218.1	BofuT4_P142510.1	AN2094	Nc_DopA; NCU09219.2; cellular morphogenesis regulator	Fruiting Body Development
SS1G_07526.1	BC1G_05076.1	BofuT4_P129990.1	AN2330 (*lsdA*)	Late sexual development	Fruiting Body Development
SS1G_01657.1	BC1G_14780.1	Not found	AN1967 (*ppoA*)	Fatty acid oxygenase, sexual-asexual development balance	Fruiting Body Development
SS1G_10705.1	BC1G_04254.1	BofuT4_P094090.1	AN1967 (*ppoA*)	Fatty acid oxygenase, sexual-asexual development balance	Fruiting Body Development
SS1G_08963.1	BC1G_12309.1	BofuT4_P151640.1	AN6505	rcoA; WD domain, G-beta repeat; *Aspergillus* homolog maybe downstream of veA	Fruiting Body Development
SS1G_10366.1	BC1G_10441.1	BofuT4_P093440.1	AN3152 (*nsdD*)	GATA-transcription factor	Fruiting Body Development
SS1G_12699.1	BC1G_05192.1	BofuT4_P101330.1	AN1848&AN5170 (*prot1* & *rosA*)	Cys6-Zn2 transcriptional activator	Fruiting Body Development
SS1G_00169.1	BC1G_06118.1	BofuT4_P017330.1	AN2002	Sc_RAM1/STE16; CAAX-farnesyl-transferase β subunit; a-factor modification	Mating Process
SS1G_00428.1	BC1G_00681.1	Not found	AN6528	Sc_RCE1; CAAX prenyl protease a-factor C-terminal processing	Mating Process

SS1G_04003.1	BC1G_15147.1	Not found	No ortholog	Mating-type locus associated gene of unknown function	Mating Process
SS1G_04004.1	BC1G_15148.1	BofuT4_P160330.1 (truncated)	AN2755 (*matB*)	Mating-type (α-box domain transcriptional activator)	Mating Process
SS1G_04006.1	Not found	BofuT4_P160320.1	AN4734 (*matA*)	Mating-type (HMG: high mobility group transcriptional activator)	Mating Process
SS1G_04584.1	BC1G_00012.1	BofuT4_P050100.1	AN2885	HMG (high mobility group) box, SAM domain (Sterile α motif); Non-mating type associated HMG.	Uncharacterized
SS1G_05340.1	BC1G_07079.1	BofuT4_P054270.1	AN1962	HMG (high mobility group) box	Fruiting Body Development
SS1G_06027.1	BC1G_01499.1	BofuT4_P041860.1	AN3583 (*kexB*)	Endoprotease for α-factor processing	Mating Process
SS1G_06080.1	BC1G_01430.1	BofuT4_P041150.1	AN3867	Sc_RAM2; CAAX-farnesyl transferase α subunit	Mating Process
SS1G_07287.1	BC1G_15198.1	BofuT4_P129120.1	AN2300 (*atrD*)	Sc_STE6; ATP-dependent efflux pump for a-factor pheromone	Mating Process
SS1G_07744.1	BC1G_13641.1	BofuT4_P006030.1	AN2946	Sc_STE13; dipeptidyl amino peptidase α-factor processing	Mating Process
SS1G_04155.1	BC1G_00201.1	BofuT4_P048160.1	No ortholog	*N. crassa ccg-4* NCU02500.2 clock-controlled pheromone precursor	Mating Process
SS1G_06124.1	BC1G_01388.1	BofuT4_P040740.1	AN2984	SRF-type transcription factor (DNA-binding and dimerisation domain); (MADS Box)	Mating Process
SS1G_00606.1	BC1G_06557.1	BofuT4_P052210.1	AN2269 (*steC*)	Sc_STE11; Serine/threonine protein kinase MKKK	Mating Signalling
SS1G_03482.1	BC1G_07009.1	BofuT4_P043580.1	AN0081 (*sfaD*)	Sc_STE 4; G protein β-subunit	Mating Signalling
SS1G_07136.1	BC1G_10211.1	BofuT4_P086790.1	AN2290 (*steA*)	Sc_Ste12; Transcriptional Activator. Homeodomain DNA binding	Mating Signalling
SS1G_07464.1	BC1G_07387.1	BofuT4_P103670.1	AN7743 (*preA*)	Sc_STE 3; Pheromone Receptor (for a-factor like pheromone)	Mating Signalling
SS1G_10310.1	BC1G_13582.1	BofuT4_P092750.1	AN2520 (*preB*)	Sc_STE 2; Pheromone Receptor (for alpha-factor like pheromone)	Mating Signalling
SS1G_11866.1	BC1G_13966.1	BofuT4_P067340.1	AN3719 (*mpkB*)	Sc_FUS3; MAP kinase	Mating Signalling
SS1G_12343.1	BC1G_01681.1	BofuT4_P134310.1	AN0651 (*fadA*)	Sc_GPA1; G protein α-subunit	Mating Signalling
