# Supplementary material for: Genomic Analysis of the Necrotrophic Fungal Pathogens Sclerotinia sclerotiorum and Botrytis cinerea
Source: PLoS Genet. 2011 Aug 18;7(8):e1002230. doi: 10.1371/journal.pgen.1002230 (PMC3158057; doi:10.1371/journal.pgen.1002230)
Supplement: Table S15 — Orthologous genes shared between S. sclerotiorum, B. cinerea and sclerotia-producing Aspergilli, A.flavus and A. oryzae. (PDF) [file pgen.1002230.s026.pdf]

Table S15

Orthologous genes shared between *S.* sclerotiorum, *B. cinerea* and sclerotia-producing Aspergilli, *A.flavus* and *A. oryzae.*

S. sclerotiorum	*B. cinerea* B05.10	*A. flavus*	*A. oryzae*	Superfamily description
SS1G_00009.1	BC1G_15446.1	AFL2G_08060	AO090120000022	alpha/beta-Hydrolases
SS1G_00125.1	BC1G_06172.1	AFL2G_04399	AO090023000561	Protein kinase-like (PK-like)
SS1G_00256.1	BC1G_00904.1	AFL2G_04296	AO090023000454	Mitochondrial carrier
SS1G_00276.1	BC1G_00884.1	AFL2G_02646	AO090003000333	No conserved domain identified
SS1G_00315.1	BC1G_00824.1	AFL2G_04138	AO090023000271	MFS general substrate transporter
SS1G_00345.1	BC1G_00788.1	AFL2G_02122	AO090003000902	No conserved domain identified
SS1G_00422.1	BC1G_00691.1	AFL2G_04933	AO090011000145	MFS general substrate transporter
SS1G_00509.1	BC1G_00576.1	AFL2G_07437	AO090001000208	(Trans)glycosidases
SS1G_00550.1	BC1G_01936.1	AFL2G_11292	AO090010000025	alpha/beta-Hydrolases
SS1G_00595.1	BC1G_06573.1	AFL2G_10113	AO090009000613	No conserved domain identified
SS1G_00617.1	BC1G_06548.1	AFL2G_03002	AO090012000070	alpha/beta-Hydrolases
SS1G_00646.1	BC1G_06494.1	AFL2G_05436	AO090011000701	NAD(P)-binding Rossmann-fold domains
SS1G_00702.1	BC1G_02405.1	AFL2G_04690	AO090023000878	SAM-dependent methyltransferases, DNA-binding domain
SS1G_00805.1	BC1G_02106.1	AFL2G_07466	AO090001000234	FAD-binding domain
SS1G_00814.1	BC1G_02108.1	AFL2G_07506	AO090001000275	Zn2/Cys6 DNA-binding domain
SS1G_00855.1	BC1G_01825.1	AFL2G_06018	AO090701000392	No conserved domain identified
SS1G_00859.1	BC1G_09031.1	AFL2G_10419	AO090009000249	No conserved domain identified
SS1G_00864.1	BC1G_09027.1	AFL2G_04840	AO090011000034	No conserved domain identified
SS1G_01029.1	BC1G_13339.1	AFL2G_04375	AO090023000535	No conserved domain identified
SS1G_01111.1	BC1G_12897.1	AFL2G_11522	AO090010000345	No conserved domain identified
SS1G_01113.1	BC1G_12899.1	AFL2G_03057	AO090012000126	Composite domain of metallo-dependent hydrolases
SS1G_01145.1	BC1G_14852.1	AFL2G_01281	AO090005001365	MFS general substrate transporter
SS1G_01210.1	BC1G_03263.1	AFL2G_12155	AO090103000232	Acid phosphatase/Vanadium-dependent haloperoxidase
SS1G_01261.1	BC1G_03953.1	AFL2G_02995	AO090012000062	No conserved domain identified
SS1G_01304.1	BC1G_12058.1	AFL2G_11920	AO090103000499	Cytochrome P450
SS1G_01361.1	BC1G_08599.1	AFL2G_00993	AO090005001031	LigB subunit of an aromatic-ring-opening dioxygenase LigAB
SS1G_01389.1	BC1G_08574.1	AFL2G_10656	AO090020000698	Concanavalin A-like lectins/glucanases
SS1G_01397.1	BC1G_08564.1	AFL2G_02430	AO090003000572	No conserved domain identified

SS1G_01458.1	BC1G_05499.1	AFL2G_00071	AO090005000051	FAD/NAD(P)-binding domain
SS1G_01463.1	BC1G_05503.1	AFL2G_10795	AO090020000540	No conserved domain identified
SS1G_01478.1	BC1G_05525.1	AFL2G_03588	AO090012000713	SAM-dependent methyltransferases
SS1G_01500.1	BC1G_05545.1	AFL2G_12179	AO090103000206	NAD(P)-binding Rossmann-fold domains
SS1G_01503.1	BC1G_05548.1	AFL2G_12272	AO090103000097	Ankyrin repeat
SS1G_01509.1	BC1G_05556.1	AFL2G_05964	AO090701000333	alpha/beta-Hydrolases
SS1G_01558.1	BC1G_11179.1	AFL2G_10308	AO090009000373	(Trans)glycosidases
SS1G_01623.1	BC1G_02448.1	AFL2G_02625	AO090003000361	No conserved domain identified
SS1G_01984.1	BC1G_02687.1	AFL2G_02671	AO090003000309	FAD-linked reductases
SS1G_01992.1	BC1G_02694.1	AFL2G_03253	AO090012000343	No conserved domain identified
SS1G_01996.1	BC1G_02703.1	AFL2G_00828	AO090005000849	No conserved domain identified
SS1G_01997.1	BC1G_02704.1	AFL2G_11104	AO090020000186	GroES-like, NAD(P)-binding Rossmann-fold domains, FabD/lysophospholipase-like, Thiolase-like, ACP-binding domain of malonyl-CoA ACP transacylase
SS1G_02020.1	BC1G_02723.1	AFL2G_05115	AO090011000328	Acetyl-CoA synthetase-like
SS1G_02126.1	BC1G_06256.1	AFL2G_08517	AO090113000025	No conserved domain identified
SS1G_02174.1	BC1G_06065.1	AFL2G_06690	AO090026000609	Protein kinase-like (PK-like)
SS1G_02193.1	BC1G_04287.1	AFL2G_02788	AO090003000178	No conserved domain identified
SS1G_02207.1	BC1G_04303.1	AFL2G_04109	AO090023000229	No conserved domain identified
SS1G_02228.1	BC1G_04332.1	AFL2G_12217	AO090103000159	MFS general substrate transporter
SS1G_02249.1	BC1G_04369.1	AFL2G_10584	AO090009000066	No conserved domain identified
SS1G_02329.1	BC1G_06040.1	AFL2G_05123	AO090011000334	No conserved domain identified
SS1G_02331.1	BC1G_06038.1	AFL2G_10831	AO090020000500	No conserved domain identified
SS1G_02336.1	BC1G_06034.1	AFL2G_00114	AO090005000097	NAD(P)-binding Rossmann-fold domains
SS1G_02338.1	BC1G_06032.1	AFL2G_08004	AO090124000040	Thiolase-like
SS1G_02340.1	BC1G_06026.1	AFL2G_11285	AO090010000015	Cytochrome P450
SS1G_02454.1	BC1G_04987.1	AFL2G_10174	AO090009000538	NAD(P)-linked oxidoreductase
SS1G_02457.1	BC1G_04984.1	AFL2G_10544	AO090009000109	Calcium ATPase
SS1G_02612.1	BC1G_01160.1	AFL2G_06463	AO090701000905	No conserved domain identified
SS1G_02678.1	BC1G_05674.1	AFL2G_04192	AO090023000341	Zn2/Cys6 DNA-binding domain
SS1G_02686.1	BC1G_05662.1	AFL2G_08567	AO090113000082	Protein kinase-like (PK-like)
SS1G_02991.1	BC1G_14243.1	AFL2G_04286	AO090023000445	SAM-dependent methyltransferases
SS1G_02995.1	BC1G_14237.1	AFL2G_11521	AO090010000344	No conserved domain identified

SS1G_03023.1	BC1G_14669.1	AFL2G_08515	AO090113000021	NAD(P)-binding Rossmann-fold domains
SS1G_03023.1	BC1G_14669.1	AFL2G_08515	AO090113000021	FAD/NAD(P)-binding domain
SS1G_03042.1	BC1G_01100.1	AFL2G_04204	AO090023000352	No conserved domain identified
SS1G_03052.1	BC1G_06283.1	AFL2G_04139	AO090023000272	No conserved domain identified
SS1G_03080.1	BC1G_06310.1	AFL2G_08649	AO090113000178	No conserved domain identified
SS1G_03087.1	BC1G_06320.1	AFL2G_04274	AO090023000428	Metallo-dependent hydrolases
SS1G_03120.1	BC1G_07598.1	AFL2G_07536	AO090001000312	Clavaminate synthase-like
SS1G_03320.1	BC1G_09601.1	AFL2G_10840	AO090020000489	No conserved domain identified
SS1G_03387.1	BC1G_07822.1	AFL2G_01726	AO090003001342	(Trans)glycosidase, cellulose-binding domain
SS1G_03387.1	BC1G_07822.1	AFL2G_01726	AO090003001342	E set domains
SS1G_03412.1	BC1G_07846.1	AFL2G_00377	AO090005000386	No conserved domain identified
SS1G_03518.1	BC1G_08354.1	AFL2G_07153	AO090026000083	Subtilisin-like
SS1G_03620.1	BC1G_03587.1	AFL2G_00427	AO090005000437	No conserved domain identified
SS1G_03705.1	BC1G_07057.1	AFL2G_08733	AO090138000049	No conserved domain identified
SS1G_03763.1	BC1G_04109.1	AFL2G_10635	AO090009000009	NAD(P)-binding Rossmann-fold domains
SS1G_03838.1	BC1G_14765.1	AFL2G_11592	AO090010000424	NAD(P)-binding Rossmann-fold domains
SS1G_03889.1	BC1G_04459.1	AFL2G_09521	AO090102000069	No conserved domain identified
SS1G_04062.1	BC1G_00338.1	AFL2G_10873	AO090020000447	UDP-Glycosyltransferase/glycogen phosphorylase
SS1G_04092.1	BC1G_00311.1	AFL2G_03044	AO090012000109	No conserved domain identified
SS1G_04184.1	BC1G_00194.1	AFL2G_08604	AO090113000124	No conserved domain identified
SS1G_04209.1	BC1G_00237.1	AFL2G_07961	AO090124000086	Nucleotidyltransferase
SS1G_04284.1	BC1G_12203.1	AFL2G_04640	AO090023000829	No conserved domain identified
SS1G_04286.1	BC1G_12206.1	AFL2G_12188	AO090103000196	No conserved domain identified
SS1G_04299.1	BC1G_12219.1	AFL2G_10366	AO090009000308	MurD-like peptide ligases
SS1G_04337.1	BC1G_13903.1	AFL2G_08622	AO090113000144	Alkaline phosphatase-like
SS1G_04340.1	BC1G_13907.1	AFL2G_11126	AO090020000158	FAD/NAD(P)-binding domain
SS1G_04341.1	BC1G_13908.1	AFL2G_11125	AO090020000159	Terpenoid synthases
SS1G_04342.1	BC1G_13909.1	AFL2G_11124	AO090020000160	No conserved domain identified
SS1G_04355.1	BC1G_13324.1	AFL2G_04578	AO090023000767	No conserved domain identified
SS1G_04372.1	BC1G_13307.1	AFL2G_06795	AO090026000485	MFS general substrate transporter
SS1G_04486.1	BC1G_00422.1	AFL2G_08832	AO090038000014	No conserved domain identified
SS1G_04710.1	BC1G_00065.1	AFL2G_06391	AO090701000829	alpha/beta-Hydrolases
SS1G_04747.1	BC1G_00173.1	AFL2G_12441	AO090206000034	ARM repeat

SS1G_04786.1	BC1G_11826.1	AFL2G_11562	AO090010000387	Plant lectins/antimicrobial peptides
SS1G_04790.1	BC1G_11835.1	AFL2G_00892	AO090005000912	Phosphoglycerate mutase-like
SS1G_04833.1	BC1G_09059.1	AFL2G_00921	AO090005000944	No conserved domain identified
SS1G_04850.1	BC1G_09077.1	AFL2G_11356	AO090010000096	Six-hairpin glycosidases
SS1G_04864.1	BC1G_09098.1	AFL2G_08645	AO090113000175	No conserved domain identified
SS1G_04881.1	BC1G_09122.1	AFL2G_04417	AO090023000581	Periplasmic binding protein-like II
SS1G_04885.1	BC1G_09125.1	AFL2G_10933	AO090020000381	FAD/NAD(P)-binding domain
SS1G_05083.1	BC1G_07151.1	AFL2G_09507	AO090102000054	Cytidine deaminase-like
SS1G_05122.1	BC1G_11237.1	AFL2G_04926	AO090011000138	Amidase signature (AS) enzymes
SS1G_05156.1	BC1G_11198.1	AFL2G_07750	AO090038000410	Acyl-CoA N-acyltransferases (Nat)
SS1G_05166.1	BC1G_08140.1	AFL2G_10708	AO090020000639	MFS general substrate transporter
SS1G_05168.1	BC1G_08138.1	AFL2G_06208	AO090701000596	Ankyrin repeat
SS1G_05211.1	BC1G_13005.1	AFL2G_07418	AO090001000187	No conserved domain identified
SS1G_05223.1	BC1G_12991.1	AFL2G_12013	AO090103000391	SAM-dependent methyltransferases
SS1G_05262.1	BC1G_15956.1	AFL2G_09815	AO090102000404	No conserved domain identified
SS1G_05322.1	BC1G_12160.1	AFL2G_06911	AO090026000348	No conserved domain identified
SS1G_05481.1	BC1G_15225.1	AFL2G_06132	AO090701000509	No conserved domain identified
SS1G_05612.1	BC1G_09791.1	AFL2G_08081	AO090120000043	alpha/beta-Hydrolases
SS1G_05613.1	BC1G_09790.1	AFL2G_11143	AO090020000140	FAD/NAD(P)-binding domain
SS1G_05808.1	BC1G_13082.1	AFL2G_08523	AO090113000035	Zn2/Cys6 DNA-binding domain
SS1G_05901.1	BC1G_12475.1	AFL2G_04884	AO090011000086	UDP-Glycosyltransferase/glycogen phosphorylase
SS1G_05980.1	BC1G_09422.1	AFL2G_00228	AO090005000220	Cytochrome P450
SS1G_05996.1	BC1G_09406.1	AFL2G_11730	AO090010000582	Carbonic anhydrase
SS1G_06040.1	BC1G_01480.1	AFL2G_11441	AO090010000185	alpha/beta-Hydrolases
SS1G_06068.1	BC1G_01444.1	AFL2G_09761	AO090102000342	No conserved domain identified
SS1G_06133.1	BC1G_01378.1	AFL2G_06896	AO090026000363	NAD(P)-binding Rossmann-fold domains
SS1G_06158.1	BC1G_01352.1	AFL2G_00347	AO090005000353	Nucleotide-diphospho-sugar transferases
SS1G_06186.1	BC1G_13021.1	AFL2G_02610	AO090003000380	Heme-dependent catalases
SS1G_06243.1	BC1G_01918.1	AFL2G_00939	AO090005000967	NAD(P)-binding Rossmann-fold domains
SS1G_06268.1	BC1G_01723.1	AFL2G_06853	AO090026000414	No conserved domain identified
SS1G_06295.1	BC1G_15670.1	AFL2G_12351	AO090103000005	Thioredoxin-like
SS1G_06297.1	BC1G_15672.1	AFL2G_12099	AO090103000291	No conserved domain identified
SS1G_06349.1	BC1G_11288.1	AFL2G_06892	AO090026000367	Nucleoside hydrolase

SS1G_06550.1	BC1G_04829.1	AFL2G_09426	AO090166000076	DHS-like NAD/FAD-binding domain, Thiamin diphosphate-binding fold
SS1G_06610.1	BC1G_04626.1	AFL2G_11659	AO090010000495	Acyl-CoA N-acyltransferases (Nat)
SS1G_06665.1	BC1G_03382.1	AFL2G_00345	AO090005000351	SAM-dependent methyltransferases
SS1G_06904.1	BC1G_14747.1	AFL2G_09017	AO090038000213	FAD/NAD(P)-binding domain
SS1G_07093.1	BC1G_09883.1	AFL2G_03383	AO090012000481	alpha/beta-Hydrolases
SS1G_07096.1	BC1G_02285.1	AFL2G_11572	AO090010000401	alpha/beta-Hydrolases
SS1G_07229.1	BC1G_12300.1	AFL2G_08833	AO090038000015	No conserved domain identified
SS1G_07244.1	BC1G_05107.1	AFL2G_10224	AO090009000475	BAG domain
SS1G_07266.1	BC1G_14823.1	AFL2G_03925	AO090023000023	MFS general substrate transporter
SS1G_07398.1	BC1G_07321.1	AFL2G_06995	AO090026000255	No conserved domain identified
SS1G_07416.1	BC1G_07341.1	AFL2G_08623	AO090113000145	No conserved domain identified
SS1G_07460.1	BC1G_05522.1	AFL2G_00206	AO090005000192	No conserved domain identified
SS1G_07463.1	BC1G_07386.1	AFL2G_08707	AO090138000014	alpha/beta-Hydrolases
SS1G_07516.1	BC1G_05062.1	AFL2G_10142	AO090009000580	Actin-like ATPase domain
SS1G_07533.1	BC1G_16008.1	AFL2G_11712	AO090010000560	No conserved domain identified
SS1G_07699.1	BC1G_02886.1	AFL2G_09637	AO090102000202	POZ domain
SS1G_07750.1	BC1G_13646.1	AFL2G_00607	AO090005000611	ARM repeat
SS1G_07846.1	BC1G_14163.1	AFL2G_11571	AO090010000400	No conserved domain identified
SS1G_07889.1	BC1G_07782.1	AFL2G_09826	AO090102000417	Radical SAM enzymes
SS1G_07925.1	BC1G_07738.1	AFL2G_02585	AO090003000406	No conserved domain identified
SS1G_08210.1	BC1G_03477.1	AFL2G_08210	AO090120000189	Glutathione S-transferase (GST), Thioredoxin-like
SS1G_08252.1	BC1G_11527.1	AFL2G_00485	AO090005000484	MFS general substrate transporter
SS1G_08253.1	BC1G_11530.1	AFL2G_12094	AO090103000299	Zn-dependent exopeptidases
SS1G_08375.1	BC1G_08724.1	AFL2G_00494	AO090005000493	MFS general substrate transporter
SS1G_08525.1	BC1G_14398.1	AFL2G_03762	AO090012000898	FAD-linked reductase, nucleotide-binding domain
SS1G_08564.1	BC1G_07446.1	AFL2G_08641	AO090113000169	Zn2/Cys6 DNA-binding domain
SS1G_08565.1	BC1G_07447.1	AFL2G_07286	AO090001000041	Cytochrome P450
SS1G_08605.1	BC1G_03224.1	AFL2G_04355	AO090023000517	RNA-binding domain, RBD
SS1G_08612.1	BC1G_03215.1	AFL2G_11858	AO090010000720	PHP domain-like
SS1G_08645.1	BC1G_07482.1	AFL2G_06873	AO090026000388	FAD-binding domain
SS1G_08659.1	BC1G_15825.1	AFL2G_09795	AO090102000381	Cytochrome P450
SS1G_08713.1	BC1G_14966.1	AFL2G_01243	AO090005001319	alpha/beta-Hydrolases

SS1G_08838.1	BC1G_09209.1	AFL2G_03023	AO090012000087	No conserved domain identified
SS1G_08843.1	BC1G_09195.1	AFL2G_11841	AO090010000701	No conserved domain identified
SS1G_08844.1	BC1G_09194.1	AFL2G_11842	AO090010000702	Clavaminate synthase-like
SS1G_08869.1	BC1G_09173.1	AFL2G_04019	AO090023000125	alpha/beta-Hydrolases
SS1G_09046.1	BC1G_11401.1	AFL2G_04873	AO090011000074	No conserved domain identified
SS1G_09111.1	BC1G_13481.1	AFL2G_00668	AO090005000676	Homeodomain-like, Ada DNA repair protein
SS1G_09159.1	BC1G_01081.1	AFL2G_08731	AO090138000045	No conserved domain identified
SS1G_09183.1	BC1G_01051.1	AFL2G_11977	AO090103000429	Xylose isomerase-like
SS1G_09194.1	BC1G_01040.1	AFL2G_09823	AO090102000413	FAD/NAD(P)-binding domain
SS1G_09239.1	BC1G_15836.1	AFL2G_05423	AO090011000681	FAD/NAD(P)-binding domain
SS1G_09240.1	BC1G_15838.1	AFL2G_07094	AO090026000149	GroES-like, NAD(P)-binding Rossmann-fold domains, FabD/lysophospholipase-like, Thiolase-like
SS1G_09242.1	BC1G_15841.1	AFL2G_11675	AO090010000517	No conserved domain identified
SS1G_09252.1	BC1G_01004.1	AFL2G_03349	AO090012000442	UDP-Glycosyltransferase/glycogen phosphorylase
SS1G_09313.1	BC1G_02380.1	AFL2G_02232	AO090003000787	MFS general substrate transporter
SS1G_09437.1	BC1G_01329.1	AFL2G_08997	AO090038000189	PLP-binding barrel
SS1G_09449.1	BC1G_07752.1	AFL2G_00677	AO090005000688	Acetyl-CoA synthetase-like
SS1G_09511.1	BC1G_04904.1	AFL2G_12229	AO090103000146	Protein kinase-like (PK-like)
SS1G_09537.1	BC1G_14080.1	AFL2G_02727	AO090003000250	alpha/beta-Hydrolases
SS1G_09568.1	BC1G_05932.1	AFL2G_06622	AO090026000689	Ribosomal protein S19
SS1G_09576.1	BC1G_12838.1	AFL2G_11405	AO090010000144	No conserved domain identified
SS1G_09722.1	BC1G_04083.1	AFL2G_03577	AO090012000701	alpha/beta-Hydrolases
SS1G_09723.1	BC1G_16166.1	AFL2G_04300	AO090023000458	No conserved domain identified
SS1G_09782.1	BC1G_15786.1	AFL2G_07319	AO090001000075	Phospholipase C/P1 nuclease
SS1G_09823.1	BC1G_15015.1	AFL2G_05194	AO090011000422	No conserved domain identified
SS1G_09978.1	BC1G_07275.1	AFL2G_06196	AO090701000579	ClpP/crotonase
SS1G_09980.1	BC1G_07264.1	AFL2G_11063	AO090020000232	Sulfite oxidase
SS1G_10043.1	BC1G_07205.1	AFL2G_01263	AO090005001344	Concanavalin A-like lectins/glucanases
SS1G_10056.1	BC1G_12498.1	AFL2G_01128	AO090005001194	MFS general substrate transporter
SS1G_10090.1	BC1G_12546.1	AFL2G_00199	AO090005000184	Zn2/Cys6 DNA-binding domain
SS1G_10148.1	BC1G_11166.1	AFL2G_07161	AO090026000077	No conserved domain identified
SS1G_10165.1	BC1G_11144.1	AFL2G_03618	AO090012000749	Pectin lyase-like
SS1G_10410.1	BC1G_14656.1	AFL2G_07323	AO090001000084	No conserved domain identified

SS1G_10457.1	BC1G_14270.1	AFL2G_08001	AO090124000045	No conserved domain identified
SS1G_10484.1	BC1G_15353.1	AFL2G_05260	AO090011000500	No conserved domain identified
SS1G_10524.1	BC1G_15920.1	AFL2G_09741	AO090102000322	No conserved domain identified
SS1G_10547.1	BC1G_10603.1	AFL2G_04017	AO090023000123	D-aminoacid aminotransferase-like PLP-dependent enzymes
SS1G_10821.1	BC1G_03837.1	AFL2G_04940	AO090011000152	Phosphoglycerate mutase-like
SS1G_11027.1	BC1G_14917.1	AFL2G_12278	AO090103000091	alpha/beta-Hydrolases
SS1G_11142.1	BC1G_16176.1	AFL2G_10513	AO090009000138	MFS general substrate transporter
SS1G_11245.1	BC1G_05418.1	AFL2G_03457	AO090012000566	No conserved domain identified
SS1G_11272.1	BC1G_05387.1	AFL2G_12223	AO090103000152	No conserved domain identified
SS1G_11279.1	BC1G_05375.1	AFL2G_06034	AO090701000408	Clavaminate synthase-like
SS1G_11281.1	BC1G_05374.1	AFL2G_04867	AO090011000065	No conserved domain identified
SS1G_11315.1	BC1G_05338.1	AFL2G_07382	AO090001000142	No conserved domain identified
SS1G_11316.1	BC1G_05337.1	AFL2G_04280	AO090023000438	ClpP/crotonase
SS1G_11356.1	BC1G_08806.1	AFL2G_03210	AO090012000290	No conserved domain identified
SS1G_11462.1	BC1G_08937.1	AFL2G_08863	AO090038000051	NTF2-like
SS1G_11578.1	BC1G_11019.1	AFL2G_04692	AO090023000880	FAD/NAD(P)-binding domain
SS1G_11649.1	BC1G_07612.1	AFL2G_08093	AO090120000055	Metallo-hydrolase/oxidoreductase
SS1G_11853.1	BC1G_13960.1	AFL2G_04758	AO090023000947	alpha/beta-Hydrolases
SS1G_11854.1	BC1G_03055.1	AFL2G_04393	AO090023000556	Zinc beta-ribbon
SS1G_11919.1	BC1G_10320.1	AFL2G_09629	AO090102000192	MFS general substrate transporter
SS1G_11930.1	BC1G_10331.1	AFL2G_08946	AO090038000122	alpha/beta-Hydrolases
SS1G_11949.1	BC1G_13502.1	AFL2G_08615	AO090113000137	Zn2/Cys6 DNA-binding domain
SS1G_11960.1	BC1G_13515.1	AFL2G_00553	AO090005000551	HAD-like
SS1G_12064.1	BC1G_13146.1	AFL2G_06443	AO090701000880	No conserved domain identified
SS1G_12128.1	BC1G_11077.1	AFL2G_07047	AO090026000199	MFS general substrate transporter
SS1G_12200.1	BC1G_01791.1	AFL2G_07475	AO090001000243	FAD-binding domain
SS1G_12235.1	BC1G_01837.1	AFL2G_06169	AO090701000549	ClpP/crotonase
SS1G_12264.1	BC1G_01875.1	AFL2G_08985	AO090038000176	No conserved domain identified
SS1G_12281.1	BC1G_01892.1	AFL2G_03218	AO090012000299	No conserved domain identified
SS1G_12449.1	BC1G_03639.1	AFL2G_00514	AO090005000511	MFS general substrate transporter
SS1G_12679.1	BC1G_12936.1	AFL2G_02703	AO090003000277	NAD(P)-binding Rossmann-fold domains
SS1G_12746.1	BC1G_05225.1	AFL2G_04791	AO090023000987	No conserved domain identified
SS1G_12935.1	BC1G_11893.1	AFL2G_08383	AO090120000379	NAD(P)-linked oxidoreductase

SS1G_12936.1	BC1G_11892.1	AFL2G_07353	AO090001000116	No conserved domain identified
SS1G_13001.1	BC1G_05809.1	AFL2G_03945	AO090023000040	Zn2/Cys6 DNA-binding domain
SS1G_13060.1	BC1G_14040.1	AFL2G_12466	AO090206000002	No conserved domain identified
SS1G_13103.1	BC1G_15814.1	AFL2G_11286	AO090010000016	No conserved domain identified
SS1G_13186.1	BC1G_15028.1	AFL2G_01554	AO090003001537	Ferredoxin reductase-like, C-terminal NADP-linked domain
SS1G_13249.1	BC1G_06654.1	AFL2G_02991	AO090012000054	Ankyrin repeat
SS1G_13269.1	BC1G_06687.1	AFL2G_00016	AO090308000003	FAD/NAD(P)-binding domain
SS1G_13361.1	BC1G_10291.1	AFL2G_09505	AO090102000052	alpha/beta-Hydrolases
SS1G_13386.1	BC1G_06369.1	AFL2G_01586	AO090003001507	alpha/beta-Hydrolases
SS1G_13392.1	BC1G_06375.1	AFL2G_04827	AO090011000020	Galactose oxidase
SS1G_13411.1	BC1G_06396.1	AFL2G_08022	AO090124000020	No conserved domain identified
SS1G_13482.1	BC1G_06473.1	AFL2G_04012	AO090023000119	SAM-dependent methyltransferases
SS1G_13500.1	BC1G_14378.1	AFL2G_02817	AO090003000142	PH domain-like
SS1G_13556.1	BC1G_10423.1	AFL2G_06319	AO090701000742	No conserved domain identified
SS1G_13635.1	BC1G_10343.1	AFL2G_06948	AO090026000311	No conserved domain identified
SS1G_13668.1	BC1G_15542.1	AFL2G_11040	AO090020000258	No conserved domain identified
SS1G_13819.1	BC1G_07402.1	AFL2G_03207	AO090012000288	MFS general substrate transporter
SS1G_13820.1	BC1G_07404.1	AFL2G_06198	AO090701000581	No conserved domain identified
SS1G_13822.1	BC1G_07406.1	AFL2G_10336	AO090009000343	No conserved domain identified
SS1G_13845.1	BC1G_13878.1	AFL2G_08759	AO090138000081	MFS general substrate transporter
SS1G_13864.1	BC1G_13857.1	AFL2G_01985	AO090003001050	Cytochrome P450
SS1G_13909.1	BC1G_12765.1	AFL2G_04072	AO090023000192	Cytochrome P450
SS1G_14065.1	BC1G_03185.1	AFL2G_07878	AO090038000546	No conserved domain identified
SS1G_14127.1	BC1G_07620.1	AFL2G_04378	AO090023000537	No conserved domain identified
SS1G_14132.1	BC1G_01112.1	AFL2G_06975	AO090026000278	NAD(P)-binding Rossmann-fold domains
SS1G_14165.1	BC1G_07663.1	AFL2G_01650	AO090003001428	MFS general substrate transporter
SS1G_14167.1	BC1G_07666.1	AFL2G_01651	AO090003001427	No conserved domain identified
SS1G_14404.1	BC1G_09662.1	AFL2G_10166	AO090009000552	No conserved domain identified
SS1G_14424.1	BC1G_09640.1	AFL2G_10515	AO090009000136	Glyceraldehyde-3-phosphate dehydrogenase-like
