# Supplementary material for: Genomic Analysis of the Necrotrophic Fungal Pathogens Sclerotinia sclerotiorum and Botrytis cinerea
Source: PLoS Genet. 2011 Aug 18;7(8):e1002230. doi: 10.1371/journal.pgen.1002230 (PMC3158057; doi:10.1371/journal.pgen.1002230)
Supplement: Table S16 — S. sclerotiorum and B. cinerea orthologs of conidiation-associated genes in Aspergillus nidulans and Neurospora crassa. (PDF) [file pgen.1002230.s027.pdf]

Table S16

S. sclerotiorum and *B. cinerea* orthologs of conidiation-associated genes in *Aspergillus nidulans* and *Neurospora crassa*.

S. sclerotiorum	*B. cinerea* B05.10	*B. cinerea* T4	*Aspergillus nidulans*	*Neurospora crassa*
SS1G_12343.1	BC1G_01681.1	BofuT4_P134310.1	FadA (AN0651.3)	NCU06493.3 (*gna-1*)
SS1G_02651.1	BC1G_01105.1	BofuT4_P030540.1	FluG (AN4819.3)	NCU04264.2
SS1G_02974.1	BC1G_02209.1	BofuT4_P142610.1	FlbC (AN2421)	NCU03043.2
SS1G_12488.1	BC1G_03699.1	BofuT4_P022380.1	FlbD (AN0279)	NCU01312.2 (*rca-1*)
No ortholog	No ortholog	No ortholog	BrlA (AN0973)	No ortholog
SS1G_06192.1	BC1G_13028.1	BofuT4_P137230.1	AbaA (AN0422)	NCU02612.2
SS1G_10147.1	BC1G_11167.1	BofuT4_P089590.1	WetA (AN1937.3)	No ortholog
SS1G_08156.1	BC1G_13179.1	BofuT4_P105010.1	StuA (AN5836.3)	NCU01414.3 (*asm-1*)
SS1G_03665.1	BC1G_03545.1	BofuT4_P120390.1	MedA (AN6230.3)	NCU07617.3
SS1G_07626.1	BC1G_02976.1	BofuT4_P003460.1	VeA (AN1052.3)	NCU01731.3
SS1G_04810.1	BC1G_11858.1	BofuT4_P161180.1	VelB (AN0363.3)	NCU02775.2
SS1G_09707.1	BC1G_11619.1	BofuT4_P157800.1	VelC (AN2059.3)	NCU07553.3
SS1G_00156.1	BC1G_06127.1	BofuT4_P017230.1	VosA (AN1959.3)	NCU05964.2
SS1G_10705.1	BC1G_04254.1	BofuT4_P094090.1	PpoA (AN1967.3)	NCU05858.2
SS1G_01657.1	BC1G_14780.1	BofuT4_P094090.1	PpoA-like (AN1967.3)	Not found
No ortholog	No ortholog	No ortholog	No ortholog	NCU08726.3 (*fl*)
SS1G_11464.1	BC1G_08935.1/08936.1	BofuT4_P075180.1	No ortholog	NCU09205.3 (vad-6)
