# Supplementary material for: Genomic Analysis of the Necrotrophic Fungal Pathogens Sclerotinia sclerotiorum and Botrytis cinerea
Source: PLoS Genet. 2011 Aug 18;7(8):e1002230. doi: 10.1371/journal.pgen.1002230 (PMC3158057; doi:10.1371/journal.pgen.1002230)
Supplement: Table S17 — S. sclerotiorum and B. cinerea orthologs of appressorium-associated genes in Magnaporthe oryzae. (PDF) [file pgen.1002230.s028.pdf]

Table S17

S. sclerotiorum and *B. cinerea* orthologs of appressorium-associated genes in *Magnaporthe oryzae*.

S. sclerotiorum	*B. cinerea* B05.10	*B. cinerea* T4	*M. oryzae*	Function
SS1G_05586.1	BC1G_09439.1	BofuT4_P114010.1	*pls1* (MGG_12594.6)	appressorium penetration; tetraspanin
No ortholog	No ortholog	No ortholog	*mpg1* (MGG_10315.6)	adhesion; hydrophobin
No ortholog	No ortholog	No ortholog	*pth11* (MGG_05871.6)	appressorium differentiation; integral membrane protein
SS1G_11636.1& SS1G_11637.1	BC1G_10952.1	BofuT4_P155910.1	*pde1* (MGG_00111.6)	no penetration hyphae; aminophospholipid translocase (APT) family of P-type ATPase
SS1G_01851.1	BC1G_02517.1	BofuT4_P109590.1	*emp1* (MGG_00527.6)	appressorium formation; extracellular matrix protein
SS1G_01602.1	BC1G_02467.1	BofuT4_P110100.1	*atg8* (MGG_01062.6)	penetration hyphae; autophagy
SS1G_13339.1	BC1G_14516.1	BofuT4_P076990.1	*pth2* (MGG_01721.6)	penetration hyphae; carnitine O-acetyl transferase
SS1G_07136.1	BC1G_10211.1	BofuT4_P086790.1	*mst12* (MGG_12958.6)	appressorium maturation; *steA* transcription factor
SS1G_00637.1	BC1G_06518.1	BofuT4_P051820.1	*chm1* (MGG_06320.6)	appressorium formation and penetration; PAK protein kinase
No ortholog	No ortholog	No ortholog	*mas1*[#] (*asg1*;*gas1*) (MGG_12337.6)	appressorium penetration
SS1G_10311.1	BC1G_13581.1	No ortholog	*mas2*[#] (MGG_04202.6)	appressorium penetration
SS1G_11468.1 & SS1G_04934.1	BC1G_08931.1 & BC1G_14711.1	BofuT4_P075230.1 & BofuT4_P017380.1	*mas3*[#] (MGG_11595.6)	uncharacterized
SS1G_00173.1	BC1G_06114.1	BofuT4_P142900.1	*mas3*[#] (MGG_00703.6)	uncharacterized
No ortholog	No ortholog	No ortholog	*cas1* (MGG_09875.6)	uncharacterized
SS1G_14237.1	BC1G_12619.1	BofuT4_P142900.1	*mas*-related (MGG_00992.6)	uncharacterized
No ortholog	No ortholog	No ortholog	*mas*-related (MGG_02253.6)	uncharacterized

[#]These genes are all annotated as "*mas3"* in the Broad genome site. The *mas1* and *mas2* designation were taken from Xue et al 2002. Plant Cell 14:2107-2119
