# Supplementary material for: Genomic Analysis of the Necrotrophic Fungal Pathogens Sclerotinia sclerotiorum and Botrytis cinerea
Source: PLoS Genet. 2011 Aug 18;7(8):e1002230. doi: 10.1371/journal.pgen.1002230 (PMC3158057; doi:10.1371/journal.pgen.1002230)
Supplement: Table S18 — Total Carbohydrate-active enzyme and associated (CAZy) modules of S. sclerotiorum and B. cinerea compared to seven other Ascomycetes. (PDF) [file pgen.1002230.s029.pdf]

Table S18

Total Carbohydrate-active enzyme and associated (CAZy) modules of *B. cinerea* and *S. sclerotiorum* compared to seven other Ascomycetes.

The number of putative modules for each CAZy family (GH, GT, PL, CE, CBM, EXPN) in each species is represented, as well as their total number. Enzymes: GH = glycoside hydrolase, GT = glycosyltransferase, PL = polysaccharide lyase; CE =carbohydrate esterases. Auxiliary modules: CBM = carbohydrate-binding module; EXPN = plant expansin-like proteins.

Fungal species	GH	GT	PL	CE	CBM	EXPN	Enzyme modules	GH+PL+CE modules	Auxilliary modules	Total CAZY modules
Sclerotinia sclerotiorum	220	89	5	32	65	4	346	257	69	415
Botrytis cinerea T4	229	95	9	34	68	6	367	272	74	441
Blumeria graminis	61	56	0	10	14	1	127	71	15	142
Phaeosphaeria nodorum	284	95	10	53	75	4	442	347	79	521
Pyrenophora teres	249	98	10	40	60	4	397	299	64	461
Gibberella zeae	251	103	21	42	69	4	417	314	73	490
Magnaporthe oryzae	268	105	5	53	86	4	431	326	90	521
Neurospora crassa	177	76	4	22	42	1	279	203	43	322
Aspergillus niger	245	118	8	23	44	1	394	276	45	439
