# Supplementary material for: Genomic Analysis of the Necrotrophic Fungal Pathogens Sclerotinia sclerotiorum and Botrytis cinerea
Source: PLoS Genet. 2011 Aug 18;7(8):e1002230. doi: 10.1371/journal.pgen.1002230 (PMC3158057; doi:10.1371/journal.pgen.1002230)
Supplement: Table S19 — Comparison of plant cell wall (PCW) and fungal cell wall (FCW) degrading and modifying CAZyme family subsets between S. sclerotiorum and B. cinerea and seven other ascomycetes. (PDF) [file pgen.1002230.s030.pdf]

Table S19

Comparison of plant cell wall (PCW) and fungal cell wall (FCW) degrading and modifying CAZyme family subsets between *S. sclerotiorum* and *B. cinerea* and other ascomycetes. Families attributed to PCW and FCW metabolism correspond to those where the majority of family members are inferred to perform this activity. Also included are families involved in energy storage and exchange. Families like GH1, GH2, GH3 and GH5, containing members that act both on PCW and on FCW were assigned to a cell wall (CW) group. The numbers in the table are the total number of genes encoding proteins from the enzyme families whose legend is: GH = glycoside hydrolase, PL = polysaccharide lyase, CE =carbohydrate esterase. The inferred substrate preferences by CAZome analysis: C - cellulose; H - hemicellulose; HP - hemicellulose or pectin side chains; P – pectin ; PCW = C + H + HP + P. No detail is given for FCW, CW and energy components.

Fungal species	Plant cell wall degrading enzymes																																									
	cellulose							hemicellulose																HP							pectin											
	GH							GH										CE						GH						CE	GH						PL					CE
	6	7	12	45	61	74	94	10	11	26	27	29	31	35	36	39	67	1	2	3	5	15	16	43	51	53	54	62	93	12	28	78	88	95	105	115	1	3	4	9	11	8
Sclerotinia sclerotiorum	1	3	5	2	9	0	0	2	3	1	3	0	6	4	2	0	0	3	0	1	8	1	6	4	2	2	1	0	1	3	17	4	0	1	1	1	4	0	0	0	0	5
Botrytis cinerea T4	1	2	4	2	9	0	0	2	3	2	4	0	4	4	2	1	0	3	0	1	10	0	5	4	3	2	1	1	1	3	18	8	1	2	1	1	6	2	0	0	0	5
Blumeria graminis	0	0	0	0	1	0	0	0	0	0	0	0	1	0	0	0	0	2	0	1	2	0	1	0	0	0	0	0	1	0	0	1	0	0	0	0	0	0	0	0	0	0
Phaeosphaeria nodorum	4	5	4	3	30	0	1	7	7	0	3	0	11	4	2	1	1	11	1	4	11	1	2	15	2	1	1	3	3	3	4	4	1	2	3	2	4	2	4	0	0	6
Pyrenophora teres	3	3	2	3	26	0	1	4	4	0	4	4	8	4	2	0	1	5	1	3	11	1	3	16	2	1	1	2	2	3	5	1	1	2	3	1	3	3	4	0	0	2
Gibberella zeae	1	2	4	1	15	1	0	5	3	0	2	1	8	3	3	2	1	5	1	5	12	0	2	17	2	1	1	1	2	3	6	7	1	2	3	2	9	7	3	1	0	6
Magnaporthe oryzae	3	5	3	1	23	1	1	7	5	0	4	4	6	0	2	1	1	10	1	7	18	1	1	20	3	1	1	3	1	3	3	3	1	1	3	3	2	1	1	0	0	1
Neurospora crassa	3	5	1	1	14	1	1	4	2	1	0	0	5	2	1	0	1	7	0	3	3	1	1	7	1	1	1	0	2	1	2	0	0	0	1	1	1	1	1	0	0	1
Aspergillus niger	2	2	4	0	7	1	0	1	4	1	5	1	7	5	2	0	1	3	0	1	5	0	2	10	4	2	1	1	0	2	21	8	1	2	2	0	6	0	2	0	0	3

Fungal species	Fungal cell wall degrading enzymes														Fungal or plant cell wall degrading enzymes				Energy				
	GH													CE	GH				GH				
	16	17	18	20	55	64	71	72	75	76	81	85	92	4	1	2	3	5	13	15	32	37	65
Sclerotinia sclerotiorum	19	6	13	1	4	2	9	6	0	11	1	0	5	4	3	2	13	14	10	4	1	1	2
Botrytis cinerea T4	21	6	10	1	4	2	9	6	0	11	1	0	5	5	3	2	16	15	10	4	1	1	2
Blumeria graminis	9	7	8	1	1	0	0	3	0	7	1	0	2	4	0	0	1	3	4	0	0	1	0
Phaeosphaeria nodorum	18	6	17	3	3	1	1	7	1	8	2	0	7	12	2	10	16	18	7	3	4	2	1
Pyrenophora teres	15	8	11	3	4	1	0	7	0	7	2	1	6	9	3	6	13	14	8	2	3	2	1
Gibberella zeae	21	6	19	2	3	2	0	3	1	8	1	0	0	7	3	10	22	15	8	3	5	2	0
Magnaporthe oryzae	18	7	15	3	5	2	1	5	1	8	2	0	6	10	2	8	20	13	10	2	4	2	0
Neurospora crassa	14	4	12	0	6	2	6	5	1	10	1	0	2	4	1	5	9	6	10	2	1	2	0
Aspergillus niger	13	5	14	3	3	0	7	7	2	11	1	0	5	6	3	6	17	10	18	2	6	1	1

Summary

Fungal species	Plant cell wall						Fungal cell wall	Fungal or plant cell wall	Energy
	Total	C	H	HP	P	H/HP/P			
Sclerotinia sclerotiorum	106	20	40	13	33	86	81	32	18
Botrytis cinerea T4	118	18	41	15	44	100	81	36	18
Blumeria graminis	10	1	7	1	1	9	43	4	5
Phaeosphaeria nodorum	173	47	66	28	32	126	86	46	17
Pyrenophora teres	145	38	55	27	25	107	74	36	16
Gibberella zeae	151	24	53	27	47	127	73	50	18
Magnaporthe oryzae	156	37	68	32	19	119	83	43	18
Neurospora crassa	78	26	31	13	8	52	67	22	15
Aspergillus niger	119	16	38	20	45	103	77	36	28
