# Supplementary material for: Genomic Analysis of the Necrotrophic Fungal Pathogens Sclerotinia sclerotiorum and Botrytis cinerea
Source: PLoS Genet. 2011 Aug 18;7(8):e1002230. doi: 10.1371/journal.pgen.1002230 (PMC3158057; doi:10.1371/journal.pgen.1002230)
Supplement: Table S20 — Comparison of plant cell wall (PCW) and fungal cell wall (FCW) sets of CBM families between S. sclerotiorum and B. cinerea and seven ascomycetes. (PDF) [file pgen.1002230.s031.pdf]

Table S20

Comparison of plant cell wall (PCW) and fungal cell wall (FCW) sets of CBM families between *S. sclerotiorum* and *B. cinerea* and seven ascomycetes. Families attributed to binding PCW and FCW components correspond to those where the majority of the family members are inferred to perform this activity. Also presented are the families involved in Energy. The numbers in the table are the total number of genes encoding proteins from the enzyme families whose legend is: CBM = carbohydrate-binding module. The inferred substrate preferences by CAZome analysis: C - cellulose; H - hemicellulose; HP - hemicellulose and pectin side chains; PCW = C + H + HP. No detail is given for FCW or Energy.

Fungal species	Plant cell wall						Fungal cell wall							Energy		
	CBM1	CBM13	CBM63	CBM6	CBM35	CBM42	CBM12	CBM14	CBM18	CBM19	CBM24	CBM43	CBM52	CBM20	CBM21	CBM48
Sclerotinia sclerotiorum	19	1	1	0	1	1	0	0	18	0	15	1	0	3	1	3
Botrytis cinerea T4	18	1	1	0	3	1	0	0	18	0	15	1	0	3	1	3
Blumeria graminis	0	0	0	0	0	0	0	0	10	0	0	1	0	0	1	2
Phaeosphaeria nodorum	13	0	1	1	3	1	0	0	46	0	0	2	0	3	1	2
Pyrenophora teres	12	0	1	1	4	1	0	0	2	1	0	0	2	0	2	1
Gibberella zeae	12	2	2	1	2	1	0	0	35	0	1	1	0	2	2	2
Magnaporthe oryzae	22	0	1	2	3	1	0	0	43	0	0	2	1	3	1	3
Neurospora crassa	19	2	0	0	0	0	0	0	3	0	8	1	1	2	1	1
Aspergillus niger	8	1	1	0	2	1	0	1	13	0	6	4	0	1	1	3

Summary

Fungal species	Plant cell wall				Fungal cell wall	Energy
	**Total**	C	H	HP		
Sclerotinia sclerotiorum	**23**	21	0	2	34	7
Botrytis cinerea T4	**24**	20	0	4	34	7
Blumeria graminis	**0**	0	0	0	11	3
Phaeosphaeria nodorum	**19**	14	1	4	48	6
Pyrenophora teres	**19**	13	1	5	23	6
Gibberella zeae	**20**	16	1	3	37	6
Magnaporthe oryzae	**29**	23	2	4	46	7
Neurospora crassa	**21**	21	0	0	13	4
Aspergillus niger	**13**	10	0	3	24	5
