# Supplementary material for: Genomic Analysis of the Necrotrophic Fungal Pathogens Sclerotinia sclerotiorum and Botrytis cinerea
Source: PLoS Genet. 2011 Aug 18;7(8):e1002230. doi: 10.1371/journal.pgen.1002230 (PMC3158057; doi:10.1371/journal.pgen.1002230)
Supplement: Table S21 — Polysaccharide degradation-related genes that are induced upon infection. (PDF) [file pgen.1002230.s032.pdf]

Table S21

Polysaccharide degradation-related genes differentially expressed upon plant infection.

Expression levels were quantified from microarray hybridization. Comparison was made between RNA isolated from sunflower cotyledons colonized by *B. cinerea* strain T4 and RNA isolated from mycelium of *B. cinerea* strain T4 grown *in vitro*. Only genes that were at least 2-fold statistically increased or decreased in at least one fungus were listed. The different substrate categories are described in Tables S19 and S20.

**CAZy genes overexpressed in both fungi**					
**Gene ID *B.cinerea***	**Gene ID *S. sclerotiorum***	**CAZy family** [a]	**Substrate category** [b]	**Fold change infection *B. cinerea***	**Fold change infection *S. sclerotiorum***
BofuT4_P047740.1	SS1G_04207.1	GH28	P	24.4 *	41.9 *
BofuT4_P005990.1	SS1G_07749.1	GH11	H	14.6	30.5 *
BofuT4_P120850.1	SS1G_03618.1	GH11	H	6.6 *	29.4 *
BofuT4_P014350.1	SS1G_12907.1	CE5	H	43.3 *	25.9 *
BofuT4_P102210.1	SS1G_11992.1	CE12	HP	4.9 *	25.5 *
BofuT4_P118670.1	SS1G_09866.1	GH5	HP[†]	8.9 *	18.5 *
BofuT4_P026900.1	SS1G_04551.1	CE8	P	5.8	15.6 *
BofuT4_P078070.1	SS1G_13255.1	GH3	C or FCW[†]	10.0	15.2 *
BofuT4_P023220.1	SS1G_07039.1	GH28	P	2.1	14.8 *
BofuT4_P023980.1	SS1G_08104.1	CE5	HP	7.1	13.1 *
BofuT4_P026710.1	SS1G_04541.1	GH78	P	4.6 *	12.3 *
BofuT4_P061530.1	SS1G_00746.1	GH5	H[†]	154.2 *	10.6 *
BofuT4_P155280.1	SS1G_11585.1	GH53	HP	10.9 *	9.5
BofuT4_P029340.1	SS1G_02553.1	GH28	P	3.8	9.3 *
BofuT4_P114590.1	SS1G_03387.1	GH5	C or H[†]	9.4 *	8.7 *
BofuT4_P026920.1	SS1G_04552.1	GH28	P	2.2	7.9 *
BofuT4_P059250.1	SS1G_00892.1	GH6	C	60.5 *	7.5 *
BofuT4_P141390.1	SS1G_09392.1	CBM20	E	15.4 *	7.0
BofuT4_P140330.1	SS1G_02760.1	CE5	H	66.6 *	6.6 *
BofuT4_P070230.1	SS1G_03093.1	GH78	P	2.7	6.6 *
BofuT4_P125090.1	SS1G_02334.1	GH7	C	138.5 *	6.5 *
BofuT4_P030130.1	SS1G_02620.1	GH79		2.5	6.1 *
BofuT4_P008190.1	SS1G_10842.1	GH35	H	4.3 *	5.2 *
BofuT4_P067660.1	SS1G_11842.1	GH93	H	3.4	5.0 *
BofuT4_P011980.1	SS1G_10092.1	GH11	H	2.2	4.7 *
BofuT4_P062500.1	SS1G_13386.1	CE5	H	7.6 *	4.6 *
BC1G_01483.1	SS1G_06037.1	GH5	FCW or H[†]	23.7 *	4.0 *
BofuT4_P132770.1	SS1G_08208.1	GH5	H[†]	28.2 *	4.0 *
BofuT4_P035890.1	SS1G_01229.1	GH55	FCW	23.0 *	3.9
BofuT4_P047700.1	SS1G_04200.1	GH92		6.9 *	3.9
BofuT4_P038970.1	SS1G_02462.1	GH54	HP	2.4	3.6 *
BofuT4_P150570.1	SS1G_09020.1	GH7	C	16.7 *	3.5
BofuT4_P078550.1	SS1G_13501.1	GH78	P	4.4	3.3 *
BofuT4_P010470.1	SS1G_03602.1	GH51	HP	2.8	3.1 *
BofuT4_P107060.1	SS1G_01493.1	GH3	HP[†]	3.2	3.0 *
BofuT4_P006540.1	SS1G_07863.1	CBM1	PCW	24.1 *	2.7
BofuT4_P059260.1	SS1G_00891.1	GH5	C	27.0 *	2.6
BofuT4_P103120.1	SS1G_12048.1	CE12	HP	8.6 *	2.6 *
BofuT4_P010370.1	SS1G_03610.1	CE16	H	5.4 *	2.5
BofuT4_P091380.1	SS1G_09821.1	GH61	C	88.0 *	2.4
BofuT4_P103410.1	SS1G_12083.1	GH115	P	9.0	2.4 *
BofuT4_P025430.1	SS1G_14158.1	GH61	C	2.7	2.3 *
BofuT4_P086620.1	SS1G_07162.1	GH3	C or FCW[†]	12.0 *	2.2 *
BofuT4_P003870.1	SS1G_07656.1	GH61	C	19.0 *	2.2
BofuT4_P112910.1	SS1G_08837.1	GH5	C	57.0 *	2.2
BofuT4_P036980.1	SS1G_01572.1	GH35	H or HP[†]	6.0 *	2.2
BofuT4_P143540.1	SS1G_14289.1	CE16	H	12.4 *	2.1
BofuT4_P135380.1	SS1G_12191.1	GH10	H	13.9 *	2.0

CAZy genes overexpressed mainly in one fungus

**Gene ID *B.cinerea***	**Gene ID *S. sclerotiorum***	**CAZy family** [a]	**Substrate category** [b]	**Fold change infection *B. cinerea***	**Fold change infection *S. sclerotiorum***
BofuT4_P031660.1	SS1G_09251.1	GH61	C	**74.1** *	1.9
BofuT4_P109330.1	SS1G_01838.1	GH61	C	**44.0** *	1.1
BofuT4_P004830.1	SS1G_07847.1	GH3	C or H[†]	**39.5** *	1.5
BofuT4_P015800.1	SS1G_00040.1	PL1	P	**30.4** *	1.0
BofuT4_P128360.1	SS1G_12622.1	GH1	C or FCW[†]	**19.0** *	1.7
BofuT4_P043000.1	SS1G_05832.1	GH28	P	**9.9** *	-1.7
BofuT4_P103260.1	SS1G_12057.1	GH28	P	**7.3** *	1.5
BofuT4_P152970.1	SS1G_11057.1	GH28	P	**6.5** *	-1.2
BofuT4_P019100.1	SS1G_00321.1	GH81	FCW	**6.2** *	1.6
BofuT4_P122770.1	SS1G_01373.1	CBM1	PCW[†]	**4.3** *	-1.2
BofuT4_P132940.1	SS1G_08229.1	GH28	P	1.5	**24.4** *
BofuT4_P030090.1	SS1G_02618.1	GH43	HP	1.6	**9.2** *
BofuT4_P118760.1	SS1G_09857.1	GH105	P	-1.5	**6.7** *
BofuT4_P150610.1	SS1G_09129.1	GH1		1.7	**3.2** *
BofuT4_P113750.1	SS1G_05557.1	GT32		1.4	**2.6** *
BofuT4_P089070.1	SS1G_01005.1	GH31	E or FCW[†]	1.3	**2.2** *

CAZy genes underexpressed mainly in one fungus

**Gene ID *B.cinerea***	**Gene ID *S. sclerotiorum***	**CAZy family** [a]	**Substrate category** [b]	**Fold change infection *B. cinerea***	**Fold change infection *S. sclerotiorum***
BofuT4_P008230.1	SS1G_10847.1	GT2	FCW[†]	-39.5 *	-1.3
BofuT4_P060820.1	SS1G_14460.1	GH72	FCW	-16.7 *	-1.6
BofuT4_P116560.1	SS1G_01882.1	CE5	H	-7.3 *	-1.2
BofuT4_P008250.1	SS1G_10845.1	GH12	C	-4.5 *	-1.1
BofuT4_P116630.1	SS1G_01889.1	GT2	FCW[†]	-2.3 *	1.5
BofuT4_P063490.1	SS1G_05784.1	GH71	FCW	-1.44	-4.8 *
BofuT4_P034570.1	SS1G_08695.1	GH18	FCW	1.60	-4.4 *
BofuT4_P008430.1	SS1G_10867.1	GH76	FCW	1.54	-4.2 *
BofuT4_P046700.1	SS1G_04062.1	GT4		-1.14	-4.0 *
BofuT4_P116860.1	SS1G_01334.1	GH92		-1.19	-3.1 *
BofuT4_P062560.1	SS1G_13392.1	CBM32		-1.08	-2.9 *
BofuT4_P122270.1	SS1G_01422.1	GH16	FCW or H[†]	-1.83	-2.5 *
BofuT4_P030230.1	SS1G_02625.1	GT17		1.5	-2.0 *

CAZy genes differentially expressed and present in only one fungus

**Gene ID *B.cinerea***	**Gene ID *S. sclerotiorum***	**CAZy family** [a]	**Substrate category** [b]	**Fold change infection *B. cinerea***	**Fold change infection *S. sclerotiorum***
BofuT4_P061870.1		GH28	P	**11.59** *	
BofuT4_P059350.1		PL1	P	**6.22** *	
BofuT4_P041780.1		GT8		-18.25 *	
	SS1G_07331.1	GH30	E		-2.2 *
	SS1G_02128.1	GH18 + CBM18	FCW		-2.1 *

Red - Positive fold changes upon infection ; **Green** - Negative fold changes upon infection
* The differential expression is statistically significant.
[a] CAZy family annotations: GH - Glycoside Hydrolase; CE - Carbohydrate Esterase; CBM - Carbohydrate-Binding Module; GT – Glycosyltransferase; PL - Polysaccharide Lyase.
[b] CAZy substrate category: C - cellulose; H - hemicellulose; P - pectin; HP - hemicellulose/ pectin side chains; E - energy; FCW-fungal cell wall.
[†] For models from families with a wide substrate preference, a substrate prediction was made by comparison against the sequences of previously characterized proteins in CAZy.
