# Supplementary material for: Genomic Analysis of the Necrotrophic Fungal Pathogens Sclerotinia sclerotiorum and Botrytis cinerea
Source: PLoS Genet. 2011 Aug 18;7(8):e1002230. doi: 10.1371/journal.pgen.1002230 (PMC3158057; doi:10.1371/journal.pgen.1002230)
Supplement: Table S22 — Expression analysis of genes encoding secreted proteins. (PDF) [file pgen.1002230.s033.pdf]

Table S22

Expression analysis of genes encoding secreted proteins.

*Genes that are supported by microarray or EST expression data, or by OrthoMCL analysis, are shown in parentheses. Note that the number of genes on the array may be different from the number of annotated genes.

	Total on array	up-regulated *in planta*		down-regulated *in planta*	
	number of genes	number of genes	exact Fisher tests p-value	number of genes	exact Fisher tests p-value
***B. cinerea* total**	**20889**	**253**		**247**	
B. cinerea T4 CAZy	388	42	9.8xe-24	6	4.74xe-01
B. cinerea T4 secreted	872	24	5.5xe-04	37	6.3xe-10
B. cinerea T4 secreted <300aa	518 (325*)	16	1.1xe-03	15	2.2xe-03
B. cinerea T4 secreted <150aa	332 (162*)	4	1.0	7	1.3xe-01
***S. sclerotiorum* total**	**14801**	**192**		**173**	
S. sclerotiorum CAZy	358	40	7.5xe-22	10	3.0xe-02
S. sclerotiorum secreted	596	13	9.8xe-02	22	1.0xe-05
S. sclerotiorum secreted <300aa	363 (236*)	5	8.1xe-01	15	6.6xe-05
S. sclerotiorum secreted <150aa	193 (99*)	1	5.4xe-01	5	8.4xe-02
