# Supplementary material for: Genomic Analysis of the Necrotrophic Fungal Pathogens Sclerotinia sclerotiorum and Botrytis cinerea
Source: PLoS Genet. 2011 Aug 18;7(8):e1002230. doi: 10.1371/journal.pgen.1002230 (PMC3158057; doi:10.1371/journal.pgen.1002230)
Supplement: Table S23 — Genes encoding secondary metabolism key enzymes in S. sclerotiorum and B. cinerea genomes. (PDF) [file pgen.1002230.s034.pdf]

Table S23

Secondary metabolism key enzymes encoding genes in *S. sclerotiorum* and *B. cinerea* genomes.

Enzyme	*B. cinerea* specific			Common to *B. cinerea* and *S. sclerotinium*				*S. sclerotiorum* specific	
		**B05.10**	**T4**		**B05.10**	**T4**	**1980**		**1980**
**STC**	**BcSTC1**	BC1G_16381.1	bt4exctg_0277[1]						
	**BcSTC2**	BC1G_09560.1	BofuT4_P130680.1						
	**BcSTC3**	BC1G_06357.1	BofuT4_P062370.1						
	**BcSTC4**	BC1G_14308.1	BofuT4_P096230.1						
	**BcSTC5**	BC1G_10537.1	BofuT4_P162160.1						
	**BcSTC6**		BofuT4_P020710.1[2]						
**DTC**	**BcDTC1**	BC1G_13295.1	BofuT4_P044420.1					**SsFUS1**	SS1G_10711.1
	**BcDTC2**	BC1G_06148.1	BofuT4_P017060.1						
	**BcDTC3**	BC1G_06751.1	BofuT4_P132450-60.1						
**PAX**	**BcPAX1**	BC1G_01823.1	BoFuT4_P135790.1						
**PHS**				**PHS1**	BC1G_13908.1	BofuT4_P163490.1	SS1G_04341.1		
**PKS**[3]	**BcPKS11**	BC1G_09042.1	BofuT4_P059830.1	**PKS1**	BC1G_13366.1	BofuT4_P088990.1	SS1G_02356.1	**SsPKS3**	SS1G_01792.1
	**BcPKS14**	BC1G_08227.1	BofuT4_P034170-90.1	**PKS2**	BC1G_02586.1	BofuT4_P108860.1	SS1G_01807.1	**SsPKS4**	SS1G_05681.1
	**BcPKS15**	BC1G_01752.1	BofuT4_P135120.1	**PKS8**	BC1G_02704.1	BofuT4_P119030.1	SS1G_01997.1	**SsPKS5**	SS1G_06923.1
	**BcPKS16**	BC1G_10686-7	BofuT4_P152140.1	**PKS9**	BC1G_15837-9.1	bt4exctg_0246[1]	SS1G_09240.1	**SsPKS7**	SS1G_07098.1
	**BcPKS17**	BC1G_01953.1	BofuT4_P052990.1	**PKS10**	BC1G_04310.1	BofuT4_P125860.1	SS1G_02211.1	**SsPKS11**	SS1G_09446.1
	**BcPKS19**	BC1G_16074.1	BofuT4_P119530.1	**PKS12**	BC1G_06876.1	BofuT4_P088370.1	SS1G_11789.1	**SsPKS14**	SS1G_11404.1
	**BcPKS20**	BC1G_07029-30.1	BofuT4_P085340.1	**PKS13**	BC1G_14497.1	BofuT4_P077210.1	SS1G_13322.1		
				**PKS18**	BC1G_06884-5.1	BofuT4_P088290.1	SS1G_05787-8.1		
				**PKS21**	BC1G_13114.1	BofuT4_P103050.1	SS1G_03591.1		
**CHS**				**CHS1**[5]	BC1G_06032.1	BofuT4_P125060.1	SS1G_02338.1		
**PKS-NRPS**	**BcPKS3**	BC1G_00695.1	BofuT4_P020280	**PKS6**	BC1G_16086-7.1	bt4exctg_0166[1]	SS1G_09237.1	**SsPKS15**	SS1G_13641.1
	**BcPKS4**	BC1G_04786.1	BofuT4_P037780.1					**SsPKS16**	SS1G_04125.1
	**BcPKS5**	BC1G_15478-9.1	BofuT4_P085990.1						
	**BcPKS7**	BC1G_15702.1	BofuT4_P071060.1						
**NRPS**	**BcNRPS7**	BC1G_15493.1	bt4exctg_0150[1]	**NRPS1**	BC1G_07441-2.1	BofuT4_P002220.1	SS1G_08561.1		
	**BcNRPS8**	BC1G_04782.1	BofuT4_P037730.1	**NRPS2**[5]	BC1G_03511.1	BofuT4_P120010-20.1	SS1G_03693.1		
	**BcNRPS9**	BC1G_09040.1	BofuT4_P059820.1	**NRPS3**	BC1G_10927-8.1	BofuT4_P150070.1	SS1G_06185.1		
				**NRPS4**	BC1G_02495.1	BofuT4_P109840.1	SS1G_01265.1		
				**NRPS5**	BC1G_10622.1	BofuT4_P115630.1	SS1G_10563.1		
				**NRPS6**[4]	BC1G_10566-7.1	BofuT4_P162470.1	SS1G_04250.1		
**DMATS**				**DMATS1**	BC1G_08209.1	BofuT4_P033980.1	SS1G_10524.1		

Notes: Listed key enzymes are terpene synthases (STC, sesquiterpene cyclase ; DTC, diterpene cyclase; PAX, paxillin-like enzyme; FUS, fusiccocin-like enzyme ; PHS; Phytoene synthase) ; NRPS, non ribosomal peptide synthetase; PKS, polyketides synthase; CHS, chalcone synthase; DMATS, DiMethylAllyl Tryptophan Synthase.[1] These genes were localized in T4 excluded contigs and not automatically predicted. [2] T4-specific gene, [3] *B. cinerea* PKS1 to PKS20 were numbered as in Kroken *et al.* (2003), [4] NRPS6 was named by Lee *et al.* (2005). The genome of the Leotiomycete *Blumeria graminis* contains a smaller number of these key enzyme encoding genes *i.e.* 1 PKS, 1 NRPS (Spanu *et al.* 2010) and 1 CHS whose putative orthologs are indicated by [5].
