# Supplementary material for: Genomic Analysis of the Necrotrophic Fungal Pathogens Sclerotinia sclerotiorum and Botrytis cinerea
Source: PLoS Genet. 2011 Aug 18;7(8):e1002230. doi: 10.1371/journal.pgen.1002230 (PMC3158057; doi:10.1371/journal.pgen.1002230)
Supplement: Table S24 — Expression changes of genes encoding membrane transporters during mycelial and in planta growth of S. sclerotiorum and B. cinerea. (PDF) [file pgen.1002230.s035.pdf]

Table S24

Expression changes of genes encoding membrane transporters during mycelial and *in planta* growth of *S. sclerotiorum* and *B. cinerea*.

Expression levels were quantified from microarray hybridization.

'myc' or '48 hpi high expression': number of genes whose expression level in mycelium or at 48 h.p.i. is above 15 X 95th percentile of random probes.

'48hpi up': number of genes with >2-fold higher expression level in sunflower 48hpi compared to mycelium;

'48 hpi down': <2- fold lower expression level in sunflower 48hpi compared to mycelium; with asterisk: statistically significant; between parentheses: not significant.

Red shading indicates predominant enrichment of genes upregulated *in planta*.

Green shading indicates predominant enrichment of genes downregulated *in planta*.

			B. cinerea	*S. sclerotiorum*
MFS: Sugars	myc	high expression	34	30
	48hpi	high expression	42	36
		up	3* (26)	6* (14)
		down	1* (6)	3* (4)
		total on chips	68	51
MFS: MDR	myc	high expression	38	33
	48hpi	high expression	33	34
		up	2* (9)	1* (8)
		down	8* (28)	4* (11)
		total on chips	107	85
ABC	myc	high expression	21	16
	48hpi	high expression	19	18
		up	0* (2)	2* (4)
		down	2* (7)	2* (2)
		total on chips	43	33
P-type ATPases	myc	high expression	11	10
	48hpi	high expression	14	10
		up	2* (5)	0* (1)
		down	0* (0)	1* (1)
		total on chips	21	17

Mitochondrial	myc	high expression	26	24
	48hpi	high expression	25	28
		up	1* (3)	1* (2)
		down	1* (1)	0* (0)
		total on chips	37	37
Amino acids	myc	high expression	18	19
	48hpi	high expression	18	27
		up	0* (3)	1* (4)
		down	0* (3)	1* (1)
		total on chips	48	49
MATE	myc	high expression	1	1
	48hpi	high expression	1	1
		up	0* (0)	0* (0)
		down	0* (0)	0* (0)
		total on chips	2	2
Aquaporins	myc	high expression	2	0
	48hpi	high expression	2	1
		up	0* (1)	0* (1)
		down	0* (1)	0* (1)
		total on chips	11	10
