# Supplementary material for: Genomic Analysis of the Necrotrophic Fungal Pathogens Sclerotinia sclerotiorum and Botrytis cinerea
Source: PLoS Genet. 2011 Aug 18;7(8):e1002230. doi: 10.1371/journal.pgen.1002230 (PMC3158057; doi:10.1371/journal.pgen.1002230)
Supplement: Table S25 — Transcription Factor-encoding genes in S. sclerotiorum and B. cinerea, as compared to other ascomycetes. (PDF) [file pgen.1002230.s036.pdf]

Table S25

Transcription Factor-encoding genes in *S. sclerotiorum* and *B. cinerea,* as compared to other ascomycetes.

Abbreviation	Interpro domain	Description	*S. sclerotiorum*	Ssc-specific [a]	*B.cinerea* B05.10	*B. cinerea T4*	Bc-specific [b]	*G. zeae*	*M. oryzae*	*N. crassa*
Zn, GATA	IPR000679	Zn-finger, GATA type	9		8	8		7	9	6
C2H2	IPR007087 and/or IPR015880	Zn-finger, C2H2 type	95	2	109	106	4	92	88	93
C2H2/Cys6	IPR007087 and IPR001138	C6 and C2H2 transcription factor	3		4	4		4	2	3
C2H2/Homeo	IPR007087 and IPR001356	Homeobox and C2H2 transcription factor	1		1	1		6	1	1
negTR	IPR007396	Negative transcriptional regulator	1		2	1		2	1	0
bZIP	IPR004827	Basic-leucine zipper (bZIP) transcription factor	25	1	25	24	1	28	21	24
bHLH	IPR001092	Basic helix-loop-helix dimerization domain bHLH	9		9	9		16	9	15
CCAAT	IPR001289	CCAAT-binding transcription factor, subunit B	1		1	1		1	1	1
CBF/Mak21	IPR005612	CBF/Mak21 family	1		1	1		2	2	2
	IPR006629	LPS-induced tumor necrosis factor alpha	2		2	2		3	2	0
	no IPR	Zn cluster transcription factor Rds2	1		1	1		1	1	1
CBF/NF-Y/A; CBF/NF-Y	IPR003957 and IPR003958	Transcription factor CBF/NF-Y/archaeal histone, subunit A	1		1	1		1	1	1
CBF/NF-Y	IPR003958 only	Transcription factor CBF/NF-Y/archaeal histone	4		3	4		6	5	5
Fork head	IPR001766	Fork head transcription factor	4		4	3		4	3	4
Homeo	IPR001356	Homeobox	8		7	6	2	6	6	6
Utp21	IPR007319	Utp21 specific WD40 associated putative domain	1		1	1		1	1	1
WD40	IPR007582	WD40 associated region in TFIID subunit	1		1	1		1	1	1
HMG1/2	IPR000910	HMG1/2 (high mobility group) box	8		9	8		9	10	11
Cys6	IPR001138 only	Fungal transcriptional regulatory protein (C6)	86		129	125	8	149	81	58
Cys6/fung TF	IPR001138 and IPR007219	Fungal transcriptional regulatory protein (C6)	59		70	67		106	37	55
Fung TF	IPR007219 only	Fungal specific transcription factor	10		22	18		60	29	19
		**Total**	**330**	**3**	**410**	**392**	**15**	**505**	**311**	**307**

[a] specific for *S. sclerotiorum* ; [b] specific for *B. cinerea*
