# Supplementary material for: Genomic Analysis of the Necrotrophic Fungal Pathogens Sclerotinia sclerotiorum and Botrytis cinerea
Source: PLoS Genet. 2011 Aug 18;7(8):e1002230. doi: 10.1371/journal.pgen.1002230 (PMC3158057; doi:10.1371/journal.pgen.1002230)
Supplement: Table S26 — Fungal strains used for the construction of the phylogeny shown in Figure 2. (PDF) [file pgen.1002230.s037.pdf]

Table S26

Strains used for the construction of the phylogeny shown in Figure 2.

Taxon	Isolate code	Host	Origin
Botrytis aclada	MUCL8415	*Allium cepa*	Germany
Botryotinia calthae	MUCL1089	*Caltha palustris*	Belgium
Botryotinia fuckeliana (*Botrytis cinerea*)	B05.10	Unknown	Germany
Botryotinia fuckeliana (*Botrytis cinerea*)	T4	*Lycopersicon esculentum*	France
Botrytis fabae	MUCL98	*Vicia faba*	Spain
Botryotinia ficariarum	CBS176.63	*Ficaria verna*	Belgium
Botrytis hyacinthi	MUCL442	*Hyacinthus* sp.	The Netherlands
Botryotinia porri	MUCL3234	*Allium porrum*	-
Botrytis tulipae	BT9901	*Tulipa* sp.	The Netherlands
Blumeria graminis f.sp. *hordei*	DH14	*Hordeum vulgare*	England
Dumontinia tuberosa	LMK749	*Anemone nemorosa*	Norway
Lambertella langei	LMK399	*Andromeda polyfolia*	Norway
Lambertella subrenispora	LMK5	*Aster ageratoides*	Japan
Monilinia fructicola	LMK125	*Prunus persica*	California, USA
Myriosclerotinia scirpicola	LMK735	*Scirpus maritimus*	Norway
Sclerotinia homeocarpa	LMK10	*Agrostis palustris*	Pennsylvania, USA
Sclerotinia minor	FA2-1	*Arachis hypogaea*	North Carolina, USA
Sclerotinia sclerotiorum	1980	*Phaseolus vulgaris*	Nebraska, USA
Sclerotinia species 1	H02-V28	Unknown vegetable crop	Alaska, USA
Sclerotinia trifoliorum	LMK47	*Medicago sativa*	Virginia, USA
Sclerotium cepivorum	LMK71	*Allium cepa*	New Jersey, USA
