# Supplementary material for: Genomic Analysis of the Necrotrophic Fungal Pathogens Sclerotinia sclerotiorum and Botrytis cinerea
Source: PLoS Genet. 2011 Aug 18;7(8):e1002230. doi: 10.1371/journal.pgen.1002230 (PMC3158057; doi:10.1371/journal.pgen.1002230)
Supplement: Table S27 — Primer sets for amplification of loci in the Sclerotiniaceae. (PDF) [file pgen.1002230.s038.pdf]

Table S27

Primer sets for amplification of loci in the Sclerotiniaceae.

Locus [a]	Definition	Primer	Primer DNA sequence (5'-3')	Optimal annealing temperature (ºC)
ACT	Actin	ACT512F	ATGTGCAAGGCCGGTTTCGC	58
		ACT783R	TACGAGTCCTTCTGGCCCAT	
CAL	Calmodulin	CAL-228F	GAGTTCAAGGAGGCCTTCTCCC	55
		CAL-737R	CATCTTTCTGGCCATCATGG	
G3PDH	Glyceraldehyde-3-phosphate dehydrogenase	G3PDH-Fbis	GCTGTCAACGACCCTTTCAT	58
		G3PDH-Rbis	ACCAGGAAACCAACTTGACG	
HSP60	Heat shock protein 60	HSP60for-deg	CAACAATTGAGATTYGCCCAYAAG	53
		HSP60rev-deg	GATRGATCCAGTGGTACCGAGCAT	
ITS	Internal transcribed spacer region	ITS1	TCCGTAGGTGAACCTGCGG	58
		ITS4	TCCTCCGCTTATTGATATGC	

[a] primers for the ITS-1 region are from: White TJ, Bruns T, Lee S, Taylor JW. 1990. Amplification and direct sequencing of fungal ribosomal RNA genes for phylogenetics. In: Innis MA, Gelfand DH, Snisky JJ, White TJ, editors. PCR Protocols: A guide to methods and applications. San Diego: Academic Press Inc. 315-322p.
