# Supplementary material for: Genomic Analysis of the Necrotrophic Fungal Pathogens Sclerotinia sclerotiorum and Botrytis cinerea
Source: PLoS Genet. 2011 Aug 18;7(8):e1002230. doi: 10.1371/journal.pgen.1002230 (PMC3158057; doi:10.1371/journal.pgen.1002230)
Supplement: Table S28 — Amplification methods and parameters used for phylogenetic analyses of housekeeping loci. (PDF) [file pgen.1002230.s039.pdf]

Table S28

Amplification methods and parameters used for phylogenetic analyses of housekeeping loci. The models of DNA sequence evolution are presented with I – invariant sites, and/or G – gamma-distributed sites.

Locus	Sequence length (bp)	Number of parsimony-informative sites	Model of DNA sequence evolution	Base frequencies	Rate matrix	Shape parameter for gamma distribution	Proportion of invariant sites
ACT	322	97	HKY+G	0.2630, 0.2532, 0.1877, 0.2961	TRatio=2.1062	0.5552	0
CAL	524	265	HKY+I+G	0.2822, 0.2264, 0.2097, 0.2817	TRatio=2.3818	1.6836	0.2007
G3PDH	826	168	SYM+I+G	0.2500, 0.2500, 0.2500, 0.2500	1.1780, 2.3723, 1.6754, 0.6030, 8.9360, 1.0000	0.5687	0.3386
HSP60	938	274	GTR+I+G	0.2856, 0.2313, 0.2320, 0.2511	2.0650, 5.8677, 2.3804, 0.6903, 19.2423, 1.0000	1.0232	0.4618
ITS	512	99	TrNef+G	0.2500, 0.2500, 0.2500, 0.2500	1.0000, 2.5192, 1.0000, 1.0000, 4.6637, 1.0000	0.3849	0
