# Supplementary material for: Genomic Analysis of the Necrotrophic Fungal Pathogens Sclerotinia sclerotiorum and Botrytis cinerea
Source: PLoS Genet. 2011 Aug 18;7(8):e1002230. doi: 10.1371/journal.pgen.1002230 (PMC3158057; doi:10.1371/journal.pgen.1002230)
Supplement: Table S29 — Support values for each node in the phylogeny (Figure 2). (PDF) [file pgen.1002230.s040.pdf]

Table S29

Support values for each node in the phylogeny inferred from the combined sequence data of the internal transcribed spacer region, actin, calmodulin, glyceraldehyde-3-phosphate dehydrogenase, and heat shock protein 60 (Figure 2).
Maximum likelihood bootstrap (MLBS) percentages for 1000 pseudoreplicates of the dataset, and Bayesian posterior probabilities (BPP) are provided at the corresponding nodes.

Node in the phylogeny (Fig. 2)	Support values	
	BPP	MLBS
1	1.00	68
2	0.61	-
3	1.00	92
4	0.60	-
5	1.00	91
6	1.00	93
7	1.00	83
8	1.00	94
9	1.00	96
10	1.00	89
11	1.00	90
12	1.00	90
13	1.00	96
14	0.79	-
15	0.76	-
16	1.00	91
17	1.00	98
