# Supplementary material for: Genomic Analysis of the Necrotrophic Fungal Pathogens Sclerotinia sclerotiorum and Botrytis cinerea
Source: PLoS Genet. 2011 Aug 18;7(8):e1002230. doi: 10.1371/journal.pgen.1002230 (PMC3158057; doi:10.1371/journal.pgen.1002230)
Supplement: Table S30 — S. sclerotiorum EST sequences generated by this project. (PDF) [file pgen.1002230.s041.pdf]

Table S30

S. sclerotiorum EST sequences generated by this project

Source	Center project	Raw sequences	Filtered sequences
Developing sclerotia	G781	26,688	17,533
Developing apothecium_55hrs	G786	23,035	18,885
Mycelium pH7	G787	23,040	21,333
Infected *Brassica*	G865	10,752	5,107
Infection cushion	G866	6,912	1,532
Infected tomato	G2118	15,360	13,144
Oxidative stress	G2128	15,360	13,621
Infection cushion	G2197	13,056	5,545
	**Total**	134,203	96,700
