# Supplementary material for: Genomic Analysis of the Necrotrophic Fungal Pathogens Sclerotinia sclerotiorum and Botrytis cinerea
Source: PLoS Genet. 2011 Aug 18;7(8):e1002230. doi: 10.1371/journal.pgen.1002230 (PMC3158057; doi:10.1371/journal.pgen.1002230)
Supplement: Table S31 — B. cinerea EST sequences generated by this project. (PDF) [file pgen.1002230.s042.pdf]

Table S31

B. cinerea EST sequences generated by this project.

Unisequences were assembled from 71 238 sequences which mapped and clustered on the *B. cinerea* T4 genome.

Botrytis ESTs libraries		Nb seqs	Description	Sequencing
T4	AL*	6412	Mycelium, nitrate starvation	Publicly available
	PD0ABA*	18701	Mycelium, 2 days in rich medium	Genoscope
	PD0ACA*	7284	Mycelium, pH stress	
	PD0AEA*	2984	Appressoria on teflon	
	PD0AGA*	6584	Mycelium, different stresses	
	PD0AHA*	6846	Appressoria on teflon	
B05.10	PD0ADA*	4094	Mycelium, different carbon sources	
	B2BC*	1624	Spores, inoculated on bean	Bayer Crop Sciences
	B3BC*	2946	Spores, germinated on hydrophobic surfaces	
	B4BC*	3681	Spores, germinated on bean leaves	
	B5BC*	2823	*In planta*, different stages of infection on bean and tomato leaves	
ATCC 58025 (G3)	B1BC*	5156	Axenic culture for production of ABA	
A1	BcA1*	10532	Mycelium, 4 days in rich medium	Publicly available
SAS56 X SAS405	PD0AFA*	3450	Complete apothecia (stalks + caps)	Genoscope
Total ESTs	Bot_allest4	83117 ESTs		
Total EST contigs	Bot_allest4_clctg	9667 unisequences		
