# Supplementary material for: Genomic Analysis of the Necrotrophic Fungal Pathogens Sclerotinia sclerotiorum and Botrytis cinerea
Source: PLoS Genet. 2011 Aug 18;7(8):e1002230. doi: 10.1371/journal.pgen.1002230 (PMC3158057; doi:10.1371/journal.pgen.1002230)
Supplement: Text S1 — Botrytis cinerea genetic map. (PDF) [file pgen.1002230.s043.pdf]

Text S1: *Botrytis cinerea* genetic map.

The T4 strain sequence assembly was validated and completed by a genetic map. We used the progeny of 68 individuals from a cross obtained between strains T4 and 32 (isolated from *Vitis vinifera*) kindly provided by Caroline Kunz (Université Pierre et Marie Curie, Paris). Around 400 microsatellite markers were designed from the genomic sequences of the B05.10 and the T4 strains by using the “GRAMENE” software (Temnykh *et al*.,2001). Among them, 134 were confirmed to be polymorphic between the strains T4 and 32. In addition to these microsatellite markers, a set of 144 SNP (Single Nucleotide Polymorphism) markers were identified through the comparison of the two *B. cinerea* (B05.10 and T4) genome sequences and genotyped in the progeny using the SNPlex genotyping assay (Applied Biosystems). Of these 144 SNPs between strains B05.10 and T4, 62 detected a polymorphism between the parents of the mapping population (L Gout, personal communication). The segregation of the 196 markers among the progeny was analysed using the “MAPMAKER” software (Lander *et al*., 1987) set at min LOD3 and max Distance at 37. A total of 36 linkage groups was identified (Table S1). In almost all cases, markers from the same supercontig are in the same linkage group, thereby validating the assembly data. Only two supercontigs (bt4_SuperContig_29_1 and bt4_SupSuperContig_114_320_122_1) appeared to be in conflict with the genetic map, as they contain markers that appear to be in distinct linkage groups. The most likely explanation is that these supercontigs contain at least one misplaced contig. We observed genetic linkage between markers belonging to different supercontigs, which allowed to associate several supercontigs, and even, in one case to insert a supercontig (bt4_SuperContig_17_1) inside a gap of another supercontig (bt4_SuperContig_171_1). The total length of the supercontigs appearing in the genetic map is 31 760 765 bp, which represents 80% of the T4 genomic sequence.

References

Lander ES, Green P, Abrahamson P, Barlow A, Daly MJ, Lincoln SE, Newberg LA (1987) MAPMAKER: an interactive computer package for constructing primary genetic linkage maps of experimental and natural populations. Genomics 1: 174-181.

Temnykh S, DeClerck G, Lukashova A, Lipovich L, Cartinhour S, McCouch S (2001) Computational and experimental analysis of microsatellites in rice (*Oryza sativa* L.): frequency, length variation, transposon associations, and genetic marker potential. Genome Res 11: 1441-1452.
