# Supplementary material for: Genomic Analysis of the Necrotrophic Fungal Pathogens Sclerotinia sclerotiorum and Botrytis cinerea
Source: PLoS Genet. 2011 Aug 18;7(8):e1002230. doi: 10.1371/journal.pgen.1002230 (PMC3158057; doi:10.1371/journal.pgen.1002230)
Supplement: Text S2 — Dual mating in Botrytis cinerea (teleomorph Botryotinia fuckeliana). (PDF) [file pgen.1002230.s044.pdf]

Text S2: Dual mating in *Botrytis cinerea* (teleomorph *Botryotinia fuckeliana*).

B. cinerea is unusual among pezizomycete fungi in that some isolates are capable of ‘dual mating’. Whereas most isolates act in a standard heterothallic fashion, being of MAT1-1 or MAT-1-2 identity, occasionally isolates are detected that can mate with both MAT1-1 and MAT1-2 isolates (Faretra et al., 1988; van der Vlugt-Bergmans et al., 1993). Some of these ‘dual mater’ isolates can self-fertilize and are truly homothallic. Thus, *B. cinerea* provides a rare example in which isolates exhibiting heterothallic, homothallic and dual mating ability are found within the same species. For example, genetic analysis of progeny from a cross between heterothallic *B. cinerea* isolates showed that the MAT1-1 and MAT1-2 alleles segregated largely in a 1:1 manner. However a small proportion (approximately 5%) of the progeny appeared to have been converted into dual maters (Faretra & Pollastro, 1996; van Kan, unpublished). Ordered tetrad analysis suggested that this was due to uni-directional change of MAT1-2 ascospores to the dual-mating form (Faretra & Pollastro, 1996). In general mating-type identity (and associated breeding system) is stable among filamentous ascomycete fungi, with switching of mating type only reported from very few species including *Chromocrea spinulosa, Sclerotinia trifoliorum* and certain species of *Ceratocystis* (Mathieson, 1952; Uhm & Fuji, 1983; Witthuhn et al., 2000 ; Debuchy et al 2010). The phenomenon of mating-type switching has long puzzled those working on mating and sexual development in ascomycete fungi, and has led to speculation concerning the genetic basis of this transition (Coppin et al., 1997). The only explanation to date has come from studies of *Ceratocystis* species in which a uni-directional change from a homothallic to a heterothallic state was correlated with loss of *MAT1-2* gene sequence at the mating-type locus (Witthurn et al., 2000). However, changes leading to gain of dual-mating ability and homothallism, as observed in *B. cinerea*, remain unexplained.

The organisation of *MAT* loci in dual-mater strains of *B. cinerea* was investigated. One possible explanation was that dual mating and homothallism has arisen from a *MAT1-1-1* gene becoming incorporated into the genome of an individual already carrying a *MAT1-2-1* gene, with the subsequent presence of both alpha- and HMG-domain genes leading to ability to sexually cross to isolates of both mating type and ability to self-fertilitze (Coppin *et al.*, 1997; Paoletti *et al.*, 2007). Such gene incorporation might have occurred by aberrant cross over, translocation, gene conversion or swapping of a silent gene cassette (as in *S. cerevisiae*). The *MAT* locus and bordering region was therefore sequenced in five isolates that display dual-mater behaviour, comprised of two field isolates (Bc7 and Bc12; van der Vlugt-

Bergmans et al. 1993), and two single ascospore progeny (RS11 and RS26) from a cross between heterothallic tester strains SAS56 and SAS405. As a control, the *MAT* loci were sequenced of two progeny from the same cross that displayed classical *MAT1-2* behaviour. Surprisingly, no evidence was found for the joint presence of complete *MAT1-1-1* and *MAT1-2-1* genes in the genomes of the dual-mating strains. Instead, all four dual-mater isolates (Bc7, Bc12, RS11 and RS26) exhibited a *MAT1-2* like locus. Polymorphisms were detected between the test and control isolates analysed, but none of the SNPs was consistently shared by all dual-mater isolates. Furthermore, neither PCR analysis nor Southern hybridization revealed any sequence homologous to the opposite *MAT* gene consensus domain. This excluded the possibility that the dual-mater strains had acquired a functional copy of the opposite mating-type gene elsewhere in their genome, as observed in some *Cochliobolus* and *Aspergillus* species (Yun et al., 1999; Paoletti et al., 2007; Rydholm et al., 2007). We conclude that dual mating in *B. cinerea* is not due to modifications at the *MAT* locus, but instead this phenomenon arose due to genetic changes elsewhere in the genome. It will be necessary to undertake an extended genetic crossing program to determine whether this form of mating-type switching has a monogenic or polygenic basis. It is perhaps significant that one of the few other documented examples of mating-type switching is in the related species *S. trifoliorum*. This suggests that heterothallic *Sclerotiniaceae* might be particularly prone to this phenomenon due to possible their origins from a homothallic ancestor, in which aspects of sexual development had become independent of *MAT* gene control as seen in *A. nidulans* (Paoletti et al, 2007). In parallel to our findings, there have been previous reports in which self fertility is possible in filamentous ascomyctee despite the presence of only one mating-type gene ; namely a limited number of *Neurospora* species, very rare field isolates of *Cryphonectria parasitica*, a low percentage of *Stemphylium* species (Inderbitzin et al. 2005) and certain *Glomerella* species, (Coppin et al., 1997; McGuire et al., 2004; García-Serrano et al., 2008). Same-sex mating ability has also been reported as a rare occurrence in the yeast *Candida albicans* (Alby et al., 2009) and the basidiomycete *Cryptococcus neoformans* (Lin et al., 2005; Heitman, 2009).

References

Alby K, Schaefer D, Bennett RJ (2009) Homothallic and heterothallic mating in the opportunistic pathogen *Candida albicans.* Nature 460: 890-893.

Coppin E, Debuchy R, Arnaise S, Picard M (1997) Mating types and sexual development in filamentous ascomycetes. Microbiol Mol Biol Rev 61: 411-428.

Debuchy R, Berteaux-Lecellier V & Silar P (2010). Mating systems and sexual morphogenesis in ascomycetes. In *Cellular and Molecular Biology of Filamentous Fungi* (Borkovich KA & Ebbole DJ, eds), pp. 501-535. ASM Press, Washington.

Faretra F, Pollastro S (1996) Genetic studies of the pathogenic fungus *Botryotinia fuckeliana* (*Botrytis cinerea*) by analysis of ordered tetrads. Mycol Res 100: 620-624.

Faretra F, Antonacci E, Pollastro S (1988) Sexual behaviour and mating system of *Botryotinia fuckeliana*, teleomorph of *Botrytis cinerea*. J Gen Microbiol 134: 2543–2550.

García-Serrano M, Laguna EA, Rodríguez-Guerra R, Simpson J (2008) Analysis of the *MAT1-2-1* gene of *Colletotrichum lindemuthianum.* Mycoscience 49: 312-317.

Heitman J (2009) Love the one you're with. Nature 460: 807-808.

Inderbitzin P, Harkness J, Turgeon BG, Berbee ML (2005). Lateral transfer of mating system in *Stemphylium.* Proc Natl Acad Sci USA 102: 11390-11395.

Lin X, Hull CM, Heitman J (2005) Sexual reproduction between partners of the same mating type in *Cryptococcus neoformans*. Nature 434: 1017–1021.

Mathieson MJ (1952) Ascospore dimorphism and mating type in *Chromocrea spinulosa* (Fuckel) Petch n. comb. Annals Bot 16: 449-466.

McGuire IC, Marra RE, Milgroom MG (2004) Mating-type heterokaryosis and selfing in *Cryphonectria parasitica.* Fungal Gen Biology 41: 521-533.

Paoletti M, Seymour FA, Alcocer MJC, Kaur N, Calvo AM, Archer DB, Dyer PS (2007) Mating type and the genetic basis of self-fertility in the model fungus *Aspergillus nidulans.* Current Biology 17: 1384-1389.

Rydholm C, Dyer PS, Lutzoni F (2007) DNA sequence characterization and molecular evolution of *MAT1* and *MAT2* mating-type loci of the self-compatible ascomycete mold *Neosartorya fischeri*. Eukaryotic Cell 6: 868-874.

Uhm FJ, Fujii H (1983). Heterothallism and mating type mutation in *Sclerotinia trifolium.* Phytopathology 73: 567-572.

Van der Vlugt-Bergmans CJB, Brandwagt BF, Wagemakers CAM, van't Klooster JW & van Kan JAL (1993) Genetic variation and segregation of DNA polymorphisms in *Botrytis cinerea*. Mycol Res 97: 1193-1200.

Witthuhn RC, Harrington TC, Wingfield BD, Steimel JP, Wingfield MJ (2000). Deletion of the *MAT-2* mating-type gene during uni-directional mating-type switching in *Ceratocystis.* Current Genetics 38: 48-52.

Yun S.-H, Berbee ML, Yoder OC, Turgeon BG (1999). Evolution of the fungal self-fertile reproductive life style from self-sterile ancestors. Proc Natl Acad Sci USA 96: 5592-5597.
