# Supplementary material for: Genomic Analysis of the Necrotrophic Fungal Pathogens Sclerotinia sclerotiorum and Botrytis cinerea
Source: PLoS Genet. 2011 Aug 18;7(8):e1002230. doi: 10.1371/journal.pgen.1002230 (PMC3158057; doi:10.1371/journal.pgen.1002230)
Supplement: Text S3 — Secondary metabolism gene clusters. (PDF) [file pgen.1002230.s045.pdf]

Text S3: Secondary metabolism gene clusters.

The main classes of secondary metabolites in fungi are (i) the non ribosomal peptides and amino acid-derived compounds, (ii) polyketides and fatty acid-derived compounds and (iii) terpenes (Hoffmeister and Keller, 2007). The first step of their biosynthesis relies on “key enzymes” that are (i) Non Ribosomal Peptide Synthetases (NRPS), (ii) PolyKetide Synthases (PKS) and (iii) Terpene synthases (TS), respectively.

NRPS catalyze the biosynthesis of small peptides via a mechanism independent of ribosomes. These enzymes use a set of core domains, known as modules, to accomplish peptide synthesis. A minimal module consists of three core domains: an adenylation (A) domain which activates the substrate via adenylation with ATP, a thiolation (T) or peptidyl carrier protein (PCP) domain which binds the activated substrate to a 4’-phosphopantetheine (PP) cofactor via a thioester bond and transfers the substrate to a condensation (C) domain which catalyzes peptide bond formation between adjacent substrates on the megasynthase complex (Finking and Marahiel, 2004). NRPS may be monomodular, consisting of a single A-T-C module, or multimodular consisting of repeated A-T-C modules (Bushley and Turgeon, 2010).

Fungal PKS are mainly type I iterative PKS. They contain a minimum of 3 domains: ketoacyl synthase (KS), acyl transferase (AT) and phosphopantetheine attachment site (PP or ACP). Most of these monomodular enzymes use their active sites repeatedly to synthesize a polyketide adding a two-carbon molecule (i.e. a CoA ester) to the growing chain with each condensation (Kroken et al., 2003). In addition to the three essential domains, PKS can contain optional domains that allow reducing the produced polyketide (Ketoreductase (KR), Dehydratase (DH) and Enoyl Reductase (ER) domains). PKS can also contain some additional NRPS domains (PKS-NRPS hybrids).

Fungi also have type III PKS *i.e.* CHalcone Synthases (CHS). Chalcone is an aromatic ketone that forms the central core for a variety of biological compounds (Seshime et al, 2005). Fungal secondary metabolites of the terpenes class are synthesized by different TS. SesquiTerpene Cyclases (STC) and DiTerpene Cyclases (DTC) form complex cyclic terpenes through their ability to cyclize Farnesyl PyroPhosphate (FPP) and Geranyl Geranyl PyroPhosphate (GGPP) respectively. PHytoene Synthase (PHS) forms carotenoids by the fusion of two GGPP moieties. Finally, indole diterpenes are formed by the prenyl transferases like PaxC (Saikia et al, 2008).

Usually, all genes necessary for the synthesis and transport of a secondary metabolite are clustered in one genomic locus together with the key enzyme encoding genes (Keller et al., 2005; Osbourn, 2010). These key genes were searched in the genomes of *S. sclerotiorum* and *B. cinerea* by using protein domain prediction (Interpro) and protein similarities (BLAST). Then, the surrounding 5’ and 3’ genes were investigated using the Genome Browser. The list of key enzymes identified in both species is presented in Table S23. *B. cinerea* has 43 genes encoding key enzymes, while *S. sclerotiorum* has 28.

The two species share a total of 19 key enzymes encoding genes which correspond to 17 secondary metabolism gene clusters. In most cases, these cluster have a similar gene organisation in the two species but for a quarter of them show differences in gene content and genomic localisation (PKS6, PKS9, PKS12/PKS18, PKS21 clusters). A few differences could also be observed between the two *B. cinerea* strains: the STC6 cluster is present in strain T4 but not in B05.10 and the PKS7 cluster shows a different gene order. These differences suggest that gene clusters are subject to genomic rearrangements.

B. cinerea and *S. sclerotiorum* share six mono- or multimodular NRPS encoding genes (Table S23) and their corresponding clusters suggesting that they synthesize six common peptides. Recent phylogenetic analysis of fungal NRPS by Bushley and Turgeon (2010) indicates that NRPS6 is involved in the biosynthesis of coprogen siderophore while NRPS2 and NRPS3 belong to the intracellular siderophore synthetases subfamily.

Interestingly, a DiMethylAllyl Tryptophan Synthase (DMATS) encoding gene was detected in both species. DMATS is one of the enzymes required for ergot alkaloids biosynthesis in addition to two NRPS (LPS1 and LPS2; Tudzynski et al., 1999). In *B. cinerea* and *S. sclerotiorum*, the DMATS encoding gene is not clustered with any other biosynthesis enzymes encoding genes. Therefore, there is no evidence for alkaloid biosynthesis.

B. cinerea and *S. sclerotiorum* share nine PKS and one PKS-NRPS hybrid encoding genes. Phylogenetic studies pointed out seven reducing PKS and three non-reducing PKS in *B. cinerea* (Kroken et al., 2003). Among the non-reducing PKS, *BcPKS12* and *BcPKS13* are the best candidates for melanin biosynthesis. Two other genes encoding enzymes involved in DHN melanin synthesis (scytalone dehydratase BC1G_14488/BoFuT4_077300 and hydroxynaphthalene reductase BC1G_04230/BoFuT4_077290) are adjacent to *BcPKS13* gene suggesting that these three genes define the cluster corresponding to the DHN melanin biosynthesis pathway (Pihet et al., 2009). A cluster with similar organization was detected in *S. sclerotiorum* (SS1G_13315-22). The DHN melanin cluster organization is not conserved in

all melanin producing fungi (Keller et al., 1997). Compounds produced by other PKS shared between *B. cinerea* and *S. sclerotiorum* could not be predicted from phylogenetic data. However, recent functional analyses showed that BcPKS6 and BcPKS9 are the key enzymes for biosynthesis of the phytotoxin botcinic acid in *B. cinerea* (Dalmais et al., 2011). To our knowledge, no botcinic acid was ever detected in *S. sclerotiorum* cultures even though the presence of PKS6 and PKS9 orthologs suggest that it may produce a similar polyketide.
A cluster with a type III PKS gene encoding a CHS is also shared by *B. cinerea* and *S. sclerotiorum* suggesting that both species could produce a chalcone-like metabolite.

A carotenoid gene cluster with a gene encoding a phytoene synthase as key enzyme, is also shared by *B. cinerea and S. sclerotiorum*. It contains similar genes in both species encoding a putative oxidase and an opsin photoreceptor with a conserved retinal-binding domain. This cluster could be involved in retinal biosynthesis as described in *Fusarium fujikuroi* (Prado-Cabrero et al., 2007).

References

Bushley KE, Turgeon BG (2010) Phylogenomics reveals subfamilies of fungal nonribosomal peptide synthetases and their evolutionary relationships. BMC Evol Biol 10: 26.

Dalmais B, Schumacher J, Moraga J, Le Pêcheur P, Tudzynski B, Collado IG, Viaud M (2011) The *Botrytis cinerea* phytotoxin botcinic acid requires two polyketide synthases for production and has a redundant role in virulence with botrydial. Mol Plant Pathol: DOI: 10.1111/j.1364-3703.2010.00692.x.

Finking R, Marahiel MA (2004) Biosynthesis of nonribosomal peptides. Annual Review of Microbiology 58: 453-488.

Hoffmeister D, Keller N, P. (2007) Natural products of filamentous fungi: enzymes, genes, and their regulation. Natural Product Reports 24: 393–416.

Keller N P, Hohn T M. 1997 Metabolic pathway gene clusters in filamentous fungi. Fungal Genet Biol 21:17-29.

Keller NP, Turner G, Bennett JW (2005) Fungal secondary metabolism - from biochemistry to genomics. Nat Rev Microbiol 3: 937 - 947.

Kroken S, Glass N L, Taylor J W, Yoder O C, Turgeon B G (2003) Phylogenomic analysis of type I polyketide synthase genes in pathogenic and saprobic ascomycetes. Proc Natl Acad Sci USA 100: 15670-15675.

Osbourn A (2010) Secondary metabolic gene clusters: evolutionary toolkits for chemical innovation. Trends in Genetics 26: 449-457.

Pihet M, Vandeputte P, Tronchin G, Renier G, Saulnier P, et al. (2009) Melanin is an essential component for the integrity of the cell wall of *Aspergillus fumigatus* conidia. BMC Microbiol 9: 177.

Prado-Cabrero A, Scherzinger D, Avalos J, Al-Babili S. 2007. Retinal biosynthesis in fungi: characterization of the carotenoid oxygenase CarX from *Fusarium fujikuroi.* Eukaryot Cell 6:650-657.

Saikia S, Nicholson MJ, Young C, Parker EJ, Scott B (2008) The genetic basis for indole-diterpene chemical diversity in filamentous fungi. Mycological Research 112: 184-199.

Seshime Y, Juvvadi PR, Fujii I, Kitamoto K. 2005. Discovery of a novel superfamily of type III polyketide synthases in *Aspergillus oryzae.* Biochem Biophys Res Commun. 331:253-260.

Tudzynski P, Hölter K, Correia T, Arntz C, Grammel N, Keller U. 1999. Evidence for an ergot alkaloid gene cluster in *Claviceps purpurea*. Mol Gen Genet. 26:133-41.
